# Annotated type catalogue of the Chrysididae (Insecta, Hymenoptera) deposited in the collection of Radoszkowski in the Polish Academy of Sciences, Kraków

**DOI:** 10.3897/zookeys.486.8753

**Published:** 2015-03-12

**Authors:** Paolo Rosa, Bogdan Wiśniowski, Zai-fu Xu

**Affiliations:** 1 Via Belvedere 8/d, I-20881 Bernareggio (MB), Italy; 2 Ojców National Park, 32-047 Ojców 9, Poland; 3 Department of Entomology, South China Agricultural University, Guangzhou 510640, China

**Keywords:** Chrysididae, catalogue, lectotype designation, new synonym, new combination, status revived, new name

## Abstract

A critical and annotated catalogue of 183 types of Hymenoptera Chrysididae belonging to 124 taxa housed in the Radoszkowski collection is given. Radoszkowski type material from other institutes has also been checked. Six lectotypes are designated in Kraków (ISEA-PAN): *Chrysis acceptabilis* Radoszkowski, 1891; *Chrysis persica* Radoczkowsky, 1881; *Chrysis daphnis* Mocsáry, 1889; *Chrysis lagodechii* Radoszkowski, 1889; *Chrysis remota* Mocsáry, 1889 and *Chrysis vagans* Radoszkowski, 1877. The lectotype of *Brugmoia pellucida* Radoszkowski, 1877 is designated in Moscow (MMU). Four new combinations are proposed: *Philoctetes araraticus* (Radoszkowski, 1890), **comb. n.**; *Pseudomalus hypocrita* (du Buysson, 1893), **comb. n.**; *Chrysis eldari* (Radoszkowski, 1893), **comb. n.**; and *Chrysura mlokosewitzi* (Radoszkowski, 1889), **comb. n..** Ten new synonyms are given: *Chrysis auropunctata* Mocsáry, 1889, **syn. n.** of *Chrysis angolensis* Radoszkovsky, 1881; *Chrysis chrysochlora* Mocsáry, 1889, **syn. n.** and *Chrysis viridans* Radoszkowski, 1891, **syn. n.** of *Chrysis keriensis* Radoszkowski, 1887; Chrysis angustifrons var. ignicollis Trautmann, 1926, **syn. n.** of *Chrysis eldari* (Radoszkowski, 1893); Chrysis maracandensis var. simulatrix Radoszkowski, 1891, **syn. n.** of *Chrysis maracandensis* Radoszkowski, 1877; *Chrysis pulchra* Radoszkovsky, 1880, **syn. n.** of *Spinolia dallatorreana* (Mocsáry, 1896); *Chrysis rubricollis* du Buysson, 1900, **syn. n.** of *Chrysis eldari* (Radoszkowski, 1893); *Chrysis subcoerulea* Radoszkowski, 1891, **syn. n.** of *Chrysis chlorochrysa* Mocsáry, 1889; *Chrysis therates* Mocsáry, 1889, **syn. n.** of *Chrysis principalis* Smith, 1874; and *Notozus komarowi* Radoszkowski, 1893, **syn. n.** of *Elampus obesus* (Mocsáry, 1890). One species is revaluated: *Chrysis chalcochrysa* Mocsáry, 1887. *Chrysis kizilkumiana* Rosa is the **new name** for *Chrysis uljanini* Radoszkowski & Mocsáry, 1889 *nec* Radoszkowski, 1877. Pictures of seventy-seven type specimens are given.

## Introduction

Oktawiusz Wincenty Bourmeister-Radoszkowski was an expert in Hymenoptera. He was born on August 7, 1820 in Łomża (Poland), as the son of a lawyer. Thanks to his teacher, Prof. Antoni Waga, he became interested in natural history, and especially in entomology. In 1838 Radoszkowski moved to St. Petersburg (Russia) and joined the Artillery Officers School. Once graduated, he had the opportunity to visit various parts of the Russian Empire and collect insects. Later, while teaching at the Artillery Academy in St. Petersburg, Radoszkowski organised private entomological meetings with some of his colleagues, mainly officers and officials of the Russian army. Together they decided to create the Russian Entomological Society, which was founded in 1859. Radoszkowski was a very active member of the Society, collecting funds for scientific trips, publications, and the library and establishing contacts with other entomological societies in Europe. He was vice-chairman of the Society from 1861 to 1866, and then Chairman from 1867 to 1879. As a retired general in 1879 Radoszkowski settled in Warsaw (Poland), where he continued his scientific work until his death on May 13, 1895 (Dylewska et al. 1973). The Radoszkowski collection currently contains nearly 30,000 specimens, including some Hymenoptera from Eduard Friedrich Eversmann’s collection, mainly Ichneumonidae.

Radoszkowski studied Chrysididae, Mutillidae, and Apoidea. He received specimens collected across the Russian Empire by officers and members of the Russian Entomological Society. Radoszkowski also exchanged insects with other European specialists (e.g. Chrysididae with du Buysson, Gribodo and Mocsáry) (P. Rosa pers. comm.). During his lifetime he wrote 112 papers, seventy-four of which related to systematics and faunistics of Hymenoptera. Radoszkowski described hundreds of new species of Hymenoptera and assigned eighty specific names to Chrysididae. Most of these specimens were kept in his collection, with a small part of chrysidids, mainly collected by Fedtschenko in Turkestan (Radoszkowski 1877), conserved in Moscow (MMU). Nevertheless, some of Fedtschenko’s specimens and types are also housed in Kraków and are easily identified by the printed labels in Cyrillic. Other types, described in other publications, and various specimens are also deposited in Berlin (MNHU), Budapest (HNHM), Genova (MSNG) and Paris (MNHN) and have been checked.

In 1898, three years after his death, Radoszkowski’s wife donated his collection to the Poznań Society of Friends of Science. In 1899, some types (duplicates from the type series, but in some cases also primary types) were given in exchange to the Zoological Museum of the University of Berlin, including a few Chrysididae. In 1902 the rest of the collection was given in exchange to the Polish Academy of Arts and Sciences in Kraków, and now is housed in the Institute of Systematics and Evolution of Animals at the Polish Academy of Sciences (ISEA-PAN). Other Chrysididae types were sent by Radoszkowski to the most imporant authors of his time; this is the reason why many types are nowaday spread in other museums (du Buysson (MNHN), Mocsáry (HNHM), and Gribodo (MSNG)).

Within the Chrysididae family, three species names have been dedicated to Radoszkowski: *Primeuchroeus radoszkowskii* (Gribodo, 1879), *Cleptes radoszkowskii* Mocsáry, 1889, and *Hedychrum radoszkowskyi* du Buysson, 1893. Some additional taxa, and even some genera in different families, are also dedicated to him: *Radoszkowskius* (Mutillidae) and *Radoszkowskiana* (Megachilidae).

The Radoszkowski Chrysididae collection in Kraków is housed in four large entomological boxes and includes approximately 1,140 specimens. The collection includes 183 types of Chrysididae representing 124 taxa: seventy-one holotypes, eight lectotypes, sixty-five syntypes, and thirty-nine paralectotypes. The collection houses types described by Eversmann, du Buysson, Gribodo, Mocsáry, and, most of all, Radoszkowski himself. The specimens are arranged in the systematic order left by Radoszkowski, which follows that proposed by Mocsáry (1889).

Eversmann’s Chrysididae collection is merged in the Chrysididae Radoszkowski collection in ISEA-PAN. Eversmann (1857) published only one paper on Chrysididae (Fauna Hymenopterologica Volgo-Uralensis), in which he described seven Chrysididae species: *Chrysis amoena*, *Chrysis cylindrica*, *Elampus ambiguus*, *Elampus bidentatus*, *Elampus femoralis*, *Hedychrum flavipes* and *Parnopes popovi*. All the types of these species are preserved in ISEA-PAN and five were later redescribed by Radoszkowski (Radoszkovsky 1866).

According to the visitor diary and the registration handbook of the museum, nobody has examined the entire collection since Mocsáry (around 1889) and du Buysson (1896). Only Móczár (1997) and Bohart (in the 80s of last century) borrowed some specimens of the genus *Cleptes* and some African Chrysididae. All the Chrysididae in the Radoszkowski collection were examined by P. Rosa in June 2012, and by B. Wiśniowski from January to February 2013, with a focus on the type specimens. In order to facilitate their future identifications, all types were labelled in red with clear indications of their status.

The name “Radoszkowski” was written in his publications in different ways. Four different spellings of this name exist in published papers dealing with Chrysididae: Radoczkowsky (1881), Radoszkovsky (1866, 1872, 1877 (1876), 1880 (1879), 1881), Radoszkowsky (1877 (1876), 1884), and, the most common, Radoszkowski (1877, 1887, 1889, 1890 (1889–1890), 1891a, 1891b, 1893a, 1893b).

The aim of this article is to provide label information, bibliographic data, current status, remarks for the type material, and to resolve confusion regarding previous lectotype designations, incorrect combinations, synonymies, placement in species groups and repository of these types.

## Material and methods

Terminology and classification of the genera follow Kimsey and Bohart (1991), classification of species and species groups follow Fauna Europaea (Rosa and Soon 2012), Linsenmaier (1959, 1968, 1987, 1994, 1997, 1999), and Rosa (2006a). Abbreviations used in the text are as follows: F-I, F-II, F-III, etc. = flagellum I, flagellum II, flagellum III and so on; S-II = second metasomal sternum; S-III = third metasomal sternum; TFC = transverse frontal carina.

The handwritings of Radoszkowski, Eversmann, du Buysson, and Mocsáry are easily recognized (Rosa 2009) and are helpful in the identification of the type material; almost all of the labels are easily legible, even those in Cyrillic. In only one case the labels have to be interpreted: some taxa described by Radoszkowski in 1891 (*Chrysis ambigua*, *Chrysis murgrabi*, *Chrysis nova*, *Chrysis semenovi*, *Chrysis singula*, *Chrysis subcoerulea*, *Chrysis unica*) bear the same labels – “TR-CAP” [Trans-Caspia] or Saraks [in 2 cases], but not the locality included in the text (Ashkabad). The same inconsistency was observed in other museums with types by Radoszkowski (1891).

Fedtschenko’s codes: specimens collected by Fedtschenko and published by Radoszkowski (1877) bear recognizable printed locality labels in cyrillic. The dating labels have a complicated code: the day is written on a square coloured label; the collecting month is related to the colour (lilac = April; pink = May; blue-green = June; yellow = July; dark blue = August; orange = September); the year is given by different marks: no marks (1879), black line on lower side (1870), and red line on upper side (1871). This code is necessary in order to recognize the type material in collection. The detailed list of the localities visited by Fedtschenko during his expedition to Russian Turkestan and the Kokan Khanate is given by Baker (2004).

Some selected types are here illustrated, such as the newly designated lectotypes. Photographs of the types were taken with Nikon D80 connected to the stereomicroscope Togal SCZ and stacked with the software Combine ZP; the white calibration of the photocamera was applied to reduce the blue effect of the neon light of the Togal microscope.

Types and other specimens have been examined from the following institutions:

HNHM Hungarian Natural History Museum, Budapest, Hungary.

ISEA-PAN Institute of Systematics and Evolution of Animals’ collection at the Polish Academy of Sciences, Kraków, Poland.

LZM Lund Zoological Museum, University of Lund, Sweden.

MHNG Natural History Museum, Genève, Switzerland.

MMU Zoological Museum, Moscow Lomonosov State University, Moscow, Russia.

MNHN National Natural History Museum, Paris, France.

MNHU Natural History Museum of the Humboldt-University, Berlin, Germany.

MSNG Museum of Natural History”G. Doria”, Genoa, Italy.

NMLS Natur Museum, Luzern, Switzerland.

ZIN Zoological Institute, St. Petersburg, Russia.

ZMUC Zoological Museum, University of Copenhagen, Denmark.

## Results and discussion

### Types housed in the Radoszkowski collection

#### Brugmoia pellucida

Taxon classificationAnimaliaHymenopteraChrysididae

Radoszkowski, 1877

Plate 1

Brugmoia pellucida Radoszkowski 1877: 26.

##### Type locality.

“Habitat in desertis Kisil-kum”, “Обѣ формы пойманы 10 и 15 мая 1871 г. въ пескахъ Кизилъ-кумъ” [Both specimens collected on the 10^th^ and 15^th^ of May 1871 on the sand of Kisil-kum].

##### Paralectotype

1♂ [box 62]: golden rounded label // *Brugmoia pellucida* [handwritten by Radoszkowski] // Кизилъкумъ [printed] // 12. [pink label with red line] // 48 [printed].

##### Remarks.

Kimsey and Bohart (1991) listed the holotype male in MMU, but the species was described on a syntypic series based on females (“*long. 8-9 mm.*”). We examined the specimen housed in MMU, which it is truly a female bearing the following labels: 10. [printed on pink label with red line] / Кизилъкумъ [printed] / *Brugmoia pellucida* Rad. [handwritten red label] / 10.V.1869 [handwritten after Radoszkowski]. We designate it as the lectotype of *Brugmoia pellucida* since the specimen housed in the Radoszkowski collection in ISEA-PAN is a male, and not a female, and it was collected on a different day.

Anyway, we consider the specimen in ISEA-PAN as the second syntype and therefore as the paralectotype, even if two discrepancies are found. The different date (the 12^th^ and not the 15^th^ of May 1971) could be a case of *lapsus calami*, since the red line is somehow covering the day number. The different sex could be also a case of *lapsus calami*; indeed, the specimen is bearing the main features listed in the description and the sexual dimorphic characteristics are not so obvious as in other *Euchroeus* species; Bohart himself (Kimsey and Bohart 1991) confused the sex of the specimen housed in MMU. Evidence that the specimen in ISEA-PAN is the second syntypes are: it was identified by Radoszkowski as *Brugmoia pellucida* and not as *Euchroeus quadratus*, the second *Euchroeus* collected by Fedtschenko in his journeys (Radoszkowski 1877); it was collected in the same locality, month and year (*Euchroeus quadratus* was collected on Mt. Karak on the 7^th^ of May); no other specimen of *Brugmoia pellucida* identified by Radoszkowski or collected by Fedtschenko was found in MMU, HNHM, MNHN, MNHU and MSNG. In Kimsey and Bohart (1991: 296), it is listed under the name *Brugmoia pellucida* Radoszkowski. The generic name *Euchroeus* Latreille was conserved by the International Commission on Zoological Nomenclature (ICZN, Opinion 1906).

**Plate 1.**
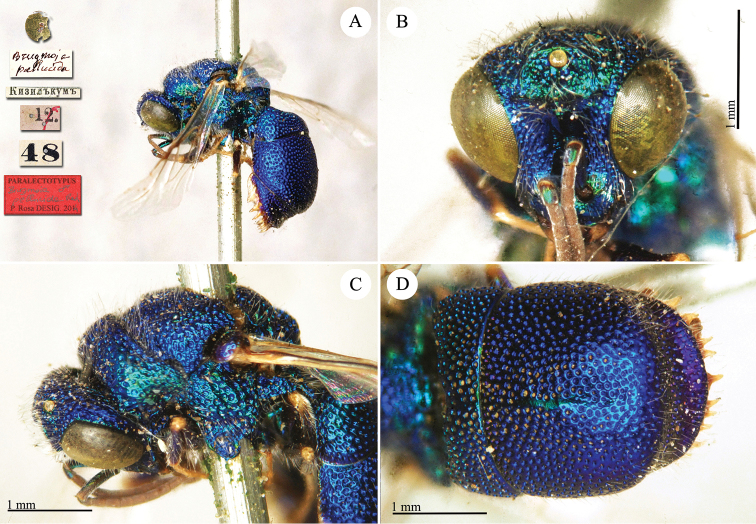
*Brugmoia pellucida* Radoszkowski, 1877, paralectotype. **A** Habitus, lateral view **B** head, frontal view **C** head and mesosoma, lateral view **D** metasoma, dorsal view.

##### Current status.

*Euchroeus pellucidus* (Radoszkowski, 1877) (transferred by du Buysson (in André) 1892: 255).

#### Chrysis abyssinica

Taxon classificationAnimaliaHymenopteraChrysididae

Radoszkowsky, 1877

Chrysis Abyssinica Radoszkowsky 1877 (1876): 148.

##### Type locality.

“Apporté par M. Raffray d’Abyssinie”.

**Holotype** [sex unknown] [box 62]: golden rounded label // label with metasoma [lost] // Abyss. Raffray [printed] [light blue label] // *abyssinica* [handwritten by Radoszkowski] // 59 [printed].

##### Remarks.

The type is seriously damaged, it lacks the metasoma.

##### Current status.

*Praestochrysis spina* (Brullé, 1846) (synonymised by Kimsey and Bohart 1991: 534).

#### Chrysis acceptabilis

Taxon classificationAnimaliaHymenopteraChrysididae

Radoszkowski, 1891

Plate 2

Chrysis acceptabilis Radoszkowski 1891a: 197.

##### Type locality.

“Saraks”.

##### Lectotype

♂ (here designated) [box 61]: golden rounded label // Tr-Cap Saraks // *acceptabilis* [handwritten by Radoszkowski].

##### Paralectotypes

2♂♂ and 1♀ [box 61]: golden rounded label // Tr-Cap Saraks.

##### Remarks.

In collection, five specimens under the name *Chrysis acceptabilis* R. bear the same collecting label. We have excluded one of them from the type series, because it belongs to another species (*Chrysis chlorochrysa*, in the *Chrysis viridissima* group) and does not match the original description.

**Plate 2.**
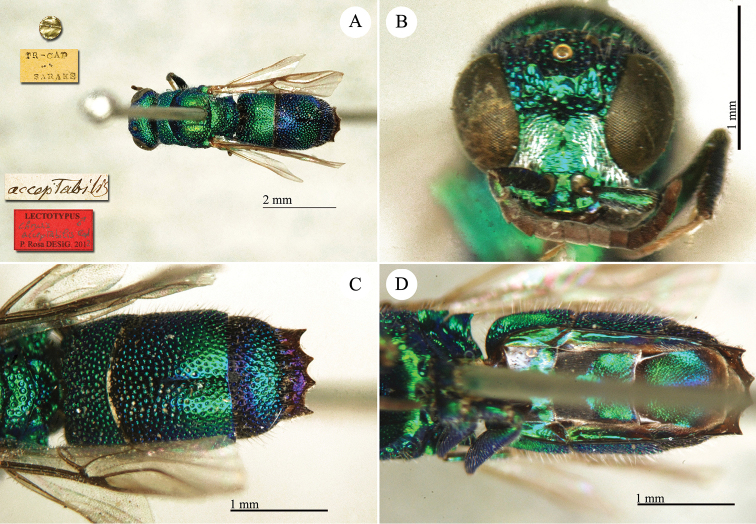
*Chrysis acceptabilis* Radoszkowski, 1891, lectotype. **A** Habitus, dorsal view **B** head, frontal view **C** metasoma, dorsal view **D** metasoma, ventral view.

Kimsey and Bohart (1991: 428) synonymised *Chrysis acceptabilis* Radoszkowski with *Chrysis kokandica* Radoszkowski and placed it in the *Chrysis splendidula* group. However, the specimen labelled by Radoszkowki is consistent with the interpretation of *Chrysis acceptabilis* provided by Linsenmaier (1968: 113). Based on its very short flagellomeres (F-I and F-II), Linsenmaier placed *Chrysis acceptabilis* in the *Chrysis cerastes* group. For this reason, Rosa et al. (2013: 15) consider *Chrysis acceptabilis* and *Chrysis kokandica* as two valid species. The examination of the type in MMU confirmed that *Chrysis kokandica* belongs to the *Chrysis splendidula* group and it is a different species, not conspecific with *Chrysis acceptabilis*. To avoid future misidentifications we designate the male specimen labelled by Radoszkowski (Plate 2) as the lectotype of *Chrysis acceptabilis*.

##### Current status.

*Chrysis acceptabilis* Radoszkowski, 1891.

#### Chrysis ambigua

Taxon classificationAnimaliaHymenopteraChrysididae

Radoszkowski, 1891

Plate 3

Chrysis ambigua Radoszkowski 1891a: 188.

##### Type locality.

“Ashabad”.

##### Syntype

1♀ [box 61]: golden rounded label // Trans-Caspia [printed] [yellow label] // *ambigua* [handwritten by Radoszkowski].

##### Remarks.

The type is damaged. It lacks the left forewing; the metasoma and two legs are glued to the locality label. Another female specimen considered as syntype was found in HNHM bearing the labels: Trans-caspia / *anceps* n. sp. *ambigua* Rad. Ashabad <handwritten by both Radoszkowski and Mocsáry> / Chrysis mutabilis v. ambigua Rad. det. Mocsáry / id nr. 115650 HNHM Hym. coll. Another syntype is housed in MNHU.

Linsenmaier (1959: 175; 1968: 112) and Rosa et al. (2013: 15) placed it in the *Chrysis cerastes* group, but Kimsey and Bohart (1991: 381) placed it in the *Chrysis taczanovskii* group.

**Plate 3.**
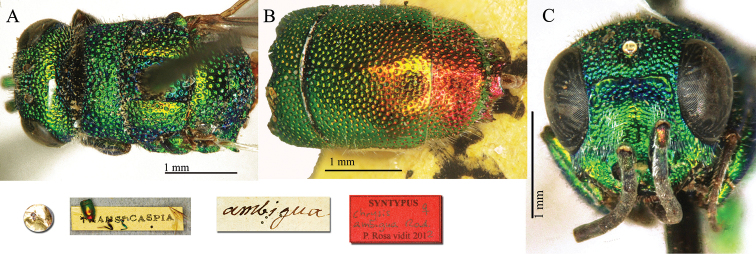
*Chrysis ambigua* Radoszkowski, 1891, syntype. **A** Head and mesosoma, dorsal view **B** metasoma, dorsal view **C** head, frontal view.

##### Current status.

*Chrysis ambigua* Radoszkowski, 1891.

#### Chrysis amoena

Taxon classificationAnimaliaHymenopteraChrysididae

Eversmann, 1857

Figure 1

Chrysis amoena Eversmann 1857: 562.

##### Type locality.

“Hab. in campis transuralensibus”.

##### Holotype

♀ [box 62]: golden rounded label // *Chrysis amoena* Evm. [handwritten by Eversmann] // brown rounded label // *Omsk* Ust K - V. [handwritten].

**Figure 1.**
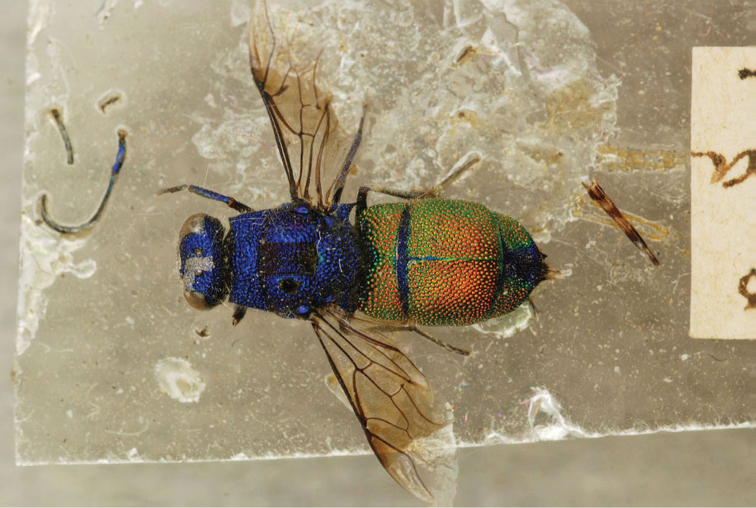
*Chrysis amoena* Eversmann, 1857, holotype, habitus, dorsal view.

##### Current status.

*Pentachrysis amoena* (Eversmann, 1857) (transferred by Kimsey and Bohart 1991: 521).

#### Chrysis analis var. incerta

Taxon classificationAnimaliaHymenopteraChrysididae

Radoszkovsky, 1880

Chrysis analis var. δ incerta Radoszkovsky 1880 (1879): 145 *nec* Dahlbom, 1854.

##### Type locality.

“Caucase” [written in the introduction].

##### Holotype

♀ [box 61]: golden rounded label // Erivan [handwritten] // 72 [printed] // *incerta* [handwritten by Radoszkowski] // *distincta* Mocs. [handwritten by Mocsáry] // *incerta* Rad *Distincta* Mocs [handwritten by Radoszkowski].

##### Remarks.

The holotype lacks fore-legs, as well as the mid- and left hind-legs. It belongs to the *Chrysis cerastes* group.

##### Current status.

*Chrysis distincta* (Mocsáry, 1887), replacement name for Chrysis analis var. incerta Radoszkovsky, 1880.

#### Chrysis analis var. perrisi

Taxon classificationAnimaliaHymenopteraChrysididae

Radoszkovsky, 1880

Plate 4

Chrysis analis var. β Perrisi Radoszkovsky 1880 (1879): 144.

##### Type locality.

“Caucasus” [written in the introduction].

##### Syntype

1♀ [box 62]: Caucasus [printed].

##### Remarks.

The name is dedicated to Abeille de Perrin and the name *perrisi* is an incorrect original spelling. Radoszkowski (1889: 25) emended the name *perrisi* to *perrini* (“*faute d’imprimerie*”). The name *perrini* was later accepted by Mocsáry (1889: 454; *Perrisi* “*e mando typographico secundum auctorem pro Perrini*”), Dalla Torre (1892: 43, sub *Chrysis perrinii*), du Buysson (1896: 17), Bischoff (1913: 47), Trautmann (1927: 171), Linsenmaier (1987: 151), but was considered as an invalid emendation by Kimsey and Bohart (1991: 382). Kimsey & Bohart placed *Chrysis perrini* in synonymy of *Chrysis analis* Spinola. We follow the interpretation given by Linsenmaier (1987), who considered *Chrysis perrini* as a valid species. The second male syntype is housed in MNHU. It belongs to the *Chrysis comparata* group.

**Plate 4.**
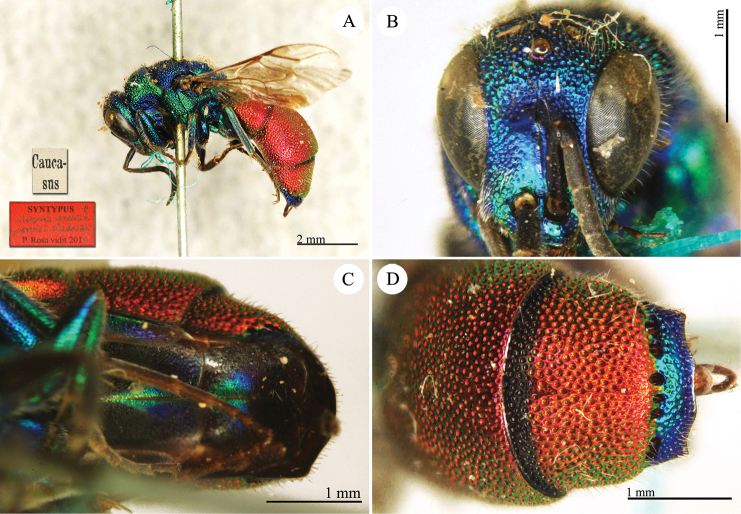
Chrysis analis var. perrisi Radoszkovsky, 1880, syntype. **A** Habitus, lateral view **B** head, frontal view **C** metasoma, ventral view **D** third metasomal tergite, dorsal view.

##### Current status.

*Chrysis perrini* Radoszkovsky, 1880 (emended by Radoszkowski 1889).

#### Chrysis analis var. rubescens

Taxon classificationAnimaliaHymenopteraChrysididae

Radoszkovsky, 1880

Plate 5

Chrysis analis var. γ rubescens Radoszkovsky 1880 (1879): 144.

##### Type locality.

“Caucase” [written in the introduction].

##### Holotype

♀ [box 61]: golden rounded label // Nikolajewka [handwritten] // Erivan [handwritten] // 68 [printed] // *rubescens* [handwritten by Radoszkowski]

##### Remarks.

The type is damaged, without both right wings and right mid-leg.

Chrysis analis var. rubescens was synonymised by Trautmann (1927: 188) with *Chrysis analis* Spinola, 1808 and his interpretation was followed by Kimsey and Bohart (1991: 382). But the type of Chrysis analis var. rubescens shows some differences with the typical European specimens of *Chrysis analis*, in the shape of head, different sculpture and black spots on S-II. The *Chrysis analis* subgroup needs revision.

**Plate 5.**
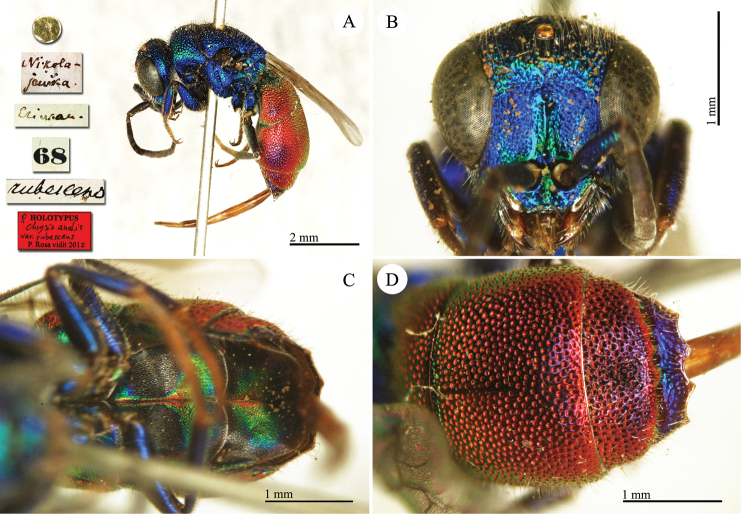
Chrysis analis var. rubescens Radoszkovsky, 1880, holotype. **A** Habitus, lateral view **B** head, frontal view **C** metasoma, ventral view **D** second and third metasomal tergites, dorsal view.

##### Current status.

*Chrysis analis* Spinola, 1808 (synonymised by Trautmann 1927: 188).

#### Chrysis annamensis

Taxon classificationAnimaliaHymenopteraChrysididae

Mocsáry, 1889

Plate 6

Chrysis (Tetrachrysis) Annamensis Mocsáry 1889: 377.

##### Type locality.

“Patria: Cochinchina (Annam, Coll. Rad.)”.

##### Holotype

♀ [box 61]: Anam Cochin [printed, sic!] [orange label] // 285 [handwritten by Mocsáry] // *annamensis* [handwritten by Radoszkowski].

##### Remarks.

*Chrysis annamensis* belongs to the *Chrysis ignita* group.

**Plate 6.**
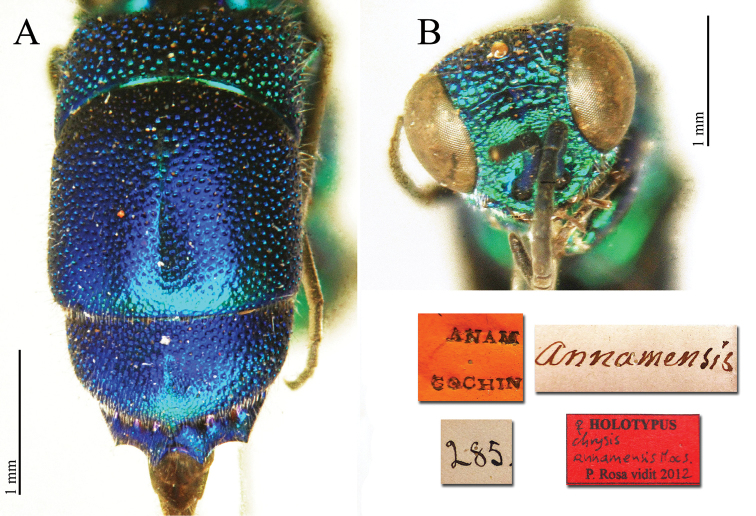
*Chrysis annamensis* Mocsáry, 1889, holotype. **A** Metasoma, dorsal view **B** head, frontal view.

##### Current status.

*Chrysis annamensis* Mocsáry, 1889.

#### Chrysis apicalis

Taxon classificationAnimaliaHymenopteraChrysididae

Radoszkovsky, 1880

Plate 7

Chrysis apicalis Radoszkovsky 1880 (1879): 146.

##### Type locality.

“Caucase” [written in the introduction].

##### Holotype

♀ [box 61]: label with metasoma glued on it // golden rounded label // Cauca Mlokos [printed] // *apicalis* [handwritten by Radoszkowski] // 58 [printed].

##### Remarks.

The type is damaged: the metasoma is glued on a separate label, the right antenna lacks the flagellum, and the left antenna lacks five flagellomeres. It belongs to the *Chrysis succincta* group.

**Plate 7.**
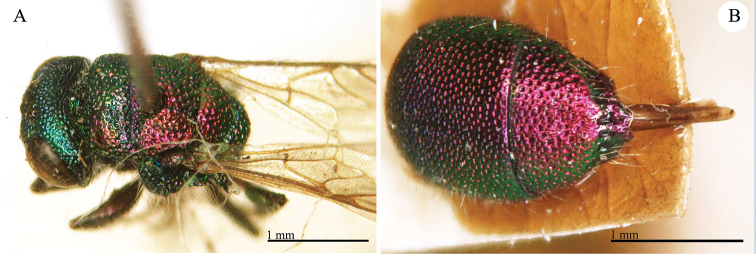
*Chrysis apicalis* Radoszkovsky, 1880, holotype. **A** Head and mesosoma, dorso-lateral view **B** second and third metasomal tergites, dorsal view.

##### Current status.

*Chrysis apicalis* Radoszkovsky, 1880.

#### Chrysis araratica

Taxon classificationAnimaliaHymenopteraChrysididae

Radoszkowski, 1890

Plate 8

Chrysis araratica Radoszkowski 1890 (1889): 509.

##### Type locality.

“Ararat, entre Sardar-Abadu et Sarabandy (13,000’)” [given in the introduction].

##### Holotype

♂ [box 61]: golden rounded label // Ararat [printed] [yellow label] // araratica R [handwritten by Radoszkowski] // Mus. PAN Kraków [handwritten by Dylewska].

##### Remarks.

Kimsey and Bohart (1991: 385) placed it in the Chrysis comparata-scutellaris group.

**Plate 8.**
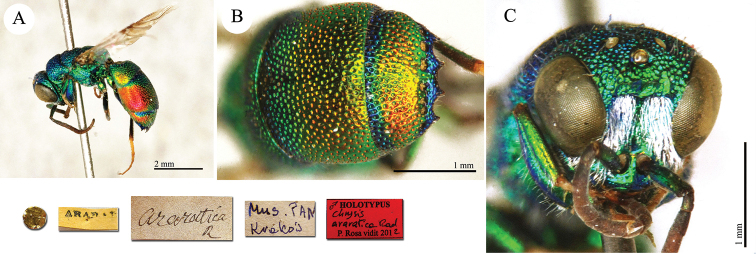
*Chrysis araratica* Radoszkowski, 1890, holotype. **A** Habitus, lateral view **B** second and third metasomal tergites, dorsal view **C** head, frontal view.

##### Current status.

*Chrysis araratica* Radoszkowski, 1890.

#### Chrysis ariadne

Taxon classificationAnimaliaHymenopteraChrysididae

Mocsáry, 1889

Chrysis (Tetrachrysis) Ariadne Mocsáry 1889: 416.

##### Type locality.

“Patria: Graecia (Morea, Mus. Caes. Vindob.! et Mus. Hung.); Caucasus (Daghestan, Coll. Rad.); territorium Transcaspicum (Coll. Rad.!)”.

##### Paralectotype

1♂ [box 61]: Trans-Caspia [printed] [yellow label] // Chrysis n.sp. Ariadne Mocs. [handwritten by Mocsáry].

##### Paralectotype

1♂ [box 61]: golden rounded label // Daghestan [printed pink label darkened with a pencil] // *ariadne* Mocs. [handwritten by Radoszkowski] // 196 [printed].

##### Paralectotype

1♂ [box 61]: Daghesta [printed pink label darkened with a pencil].

##### Remarks.

Lectotype designated by Moczár (1965: 172), preserved in HNHM. It belongs to the *Chrysis comparata-scutellaris* group.

##### Current status.

*Chrysis soror* Dahlbom, 1854 (synonymised by Linsenmaier 1959: 125).

#### Chrysis ashabadensis

Taxon classificationAnimaliaHymenopteraChrysididae

Radoszkowski, 1891

Plate 9

Chrysis ashabadensis Radoszkowski 1891a: 183.

##### Type locality.

“Ashabad”.

##### Holotype

♂ [box 60]: label with tergal segment // Trans-Caspia [printed] [yellow label] // *ashabadensi* [handwritten by Radoszkowski sic!].

##### Remarks.

The type is partly damaged: both hind-legs are missing and both antennae are broken (the left antenna lacks five flagellomeres, the right antenna lacks six); the genital capsule glued on the label is also missing; a few metasomal sternites and tergites are still glued on the label.

It belongs to the *Chrysis succincta* group and not to the *Chrysis elegans* group, as supposed by Linsenmaier (1968) and Kimsey and Bohart (1991).

**Plate 9.**
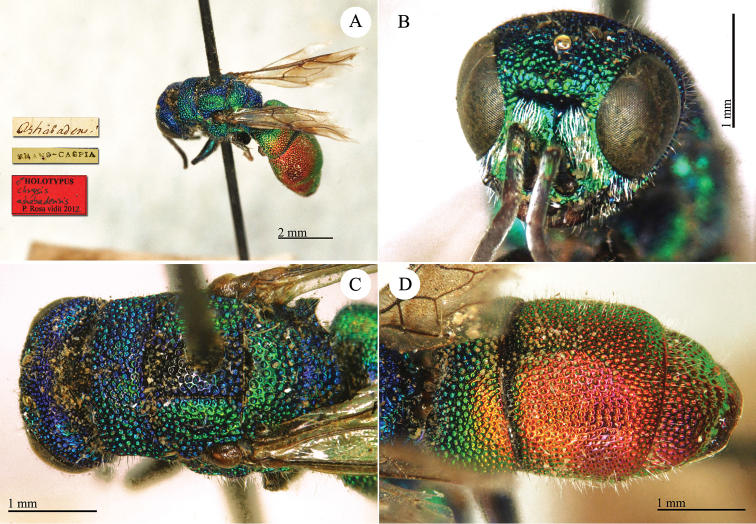
*Chrysis ashabadensis* Radoszkowski, 1891, holotype. **A** Habitus, dorso-lateral view **B** head, frontal view **C** mesosoma, dorsal view **D** metasoma, dorso-lateral view.

##### Current status.

*Chrysis ashabadensis* Radoszkowski, 1891.

#### Chrysis asiatica

Taxon classificationAnimaliaHymenopteraChrysididae

Radoszkowski, 1889

Chrysis (Tetrachrysis) asiatica Radoszkowski 1889: 26.

##### Type locality.

“Tachkent; vallée de Zarafchan”.

##### Holotype

(?) ♂ [box 61]: golden rounded label // label with genitalia // Ϲтепь м. Ϲ. д. и Т. [printed] // 19 [printed] [pink label] // *asiaticus* [handwritten by Radoszkowski] // 251 [printed].

##### Remarks.

The type is damaged: it is missing its mid- and left hind-legs; its tibia and tarsi.

Radoszkowski (1877: 21) firstly identified this species as *Chrysis analis* Spinola. Radoszkowski (1889) illustrated the genitalia of this specimen. It belongs to the *Chrysis comparata* group.

##### Current status.

*Chrysis asiatica* Radoszkowski, 1889.

#### Chrysis auropunctata

Taxon classificationAnimaliaHymenopteraChrysididae

Mocsáry, 1889

Chrysis (Tetrachrysis) auropunctata Mocsáry 1889: 474.

##### Type locality.

“Patria: Annam in Cochinchina (Coll. Rad.)”.

##### Holotype

♀ [box 61]: golden rounded label // Anam Cochin [printed] [orange label] // *auropunctata* Moc [handwritten by Radoszkowski] // 139 [printed].

##### Remarks.

The specimen represents the light green variation of *Chrysis angolensis* Radoszkovsky, 1881. Here we propose the new synonym: Chrysis (Tetrachrysis) auropunctata Mocsáry, 1889 = *Chrysis angolensis* Radoszkovsky, 1881. It belongs to the *Chrysis angolensis* group.

##### Current status.

*Chrysis angolensis* Radoszkovsky, 1881.

#### Chrysis barrei

Taxon classificationAnimaliaHymenopteraChrysididae

Radoszkowski, 1891

Chrysis Barrei Radoszkowski 1891a: 194.

##### Type locality.

“Saraks”.

##### Paralectotype

1♂ [box 61]: golden rounded label // Tr-Cap Saraks [printed] [yellow label] // *Barrei* [handwritten by Radoszkowski].

##### Remarks.

Kimsey and Bohart (1991: 479) designated the lectotype by inference of "holotype" (ICZN art. 74.6). It belongs to the *Chrysis comparata* group.

##### Current status.

*Chrysis xanthocera* Klug, 1845 (synonymised by du Buysson (in André) 1895: 523).

#### Chrysis branicki

Taxon classificationAnimaliaHymenopteraChrysididae

Radoszkovsky, 1877

Chrysis Branicki Radoszkovsky 1877 (1876): 107.

##### Type locality.

“apportée d’Egypte pendant le voyage du comte Branicki”.

##### Syntype

1♀ [box 60]: Eldar Caucas [printed].

##### Syntype

1♀ [box 60]: Caucas [printed].

##### Remarks.

The type locality is probably misinterpreted: Radoszkowski gave “Egypt” as the type locality, but the true type locality should be Caucasus. In fact, the original description is provided in a paper discussing the Russian Hymenoptera (*Matériaux pour servir à une faune hyménoptèròlogique de la Russie*) in which all of the other species described were collected in Caucasus. In the same journal, Radoszkowsky listed the material collected in Egypt by Count Branicki, the Polish nobleman who financed many scientific trips to Egypt and who sponsored Professor Waga, Radoszkowski’s teacher (*Comte-rendu des Hyménoptères recueillis en Egypte et Abyssinie en 1873*). Radoszkowski dedicated this chrysidid to Branicki, and most likely confused the localities. One syntype is also deposited in MNHU. It belongs to the *Chrysis bihamata* group.

##### Current status.

*Chrysis branickii* Radoszkovsky, 1877 (emended by Radoszkovsky 1877: 146).

#### Chrysis caucasica

Taxon classificationAnimaliaHymenopteraChrysididae

Radoszkovsky, 1877

Chrysis Caucasica Radoszkovsky 1877 (1876): 108.

##### Type locality.

“Envoyé du Caucase par Mr. Mlokosiewitz”.

##### Syntype

1♀ [box 62]: golden rounded label // Caucasus [printed] // 30 [printed] // *caucasica* [handwritten by Radoszkowski] // sexdentata Chr caucasica R. [handwritten by Radoszkowski].

##### Syntype

1♀ [box 62]: Caucasus [printed].

##### Remarks.

Mocsáry (1889: 537) synonymised it with *Chrysis sexdentata* Christ, 1791. Kimsey and Bohart (1991: 475) placed *Chrysis caucasica* and *Chrysis sexdentata* in synonymy with *Chrysis variegata* Olivier, 1791. All the authors before Kimsey and Bohart (1991) (e.g. Mocsáry 1889: 597; Dalla Torre 1892: 87; Bischoff 1913: 29; Trautmann 1927: 86) and after (e.g. Linsenmaier 1997: 286; Rosa 2005: 90, Strumia 1995), with the only exception of Mingo (1994), considered *Chrysis variegata* as a synonym of *Euchroeus purpuratus* (Fabricius, 1787). For detailed considerations see Linsenmaier (1997) and Rosa (2005). It belongs to the *Chrysis smaragdula* group *sensu* Kimsey and Bohart (1991).

##### Current status.

*Chrysis sexdentata* Christ, 1791 (synonymised by Mocsáry 1889).

#### Chrysis chalcophana

Taxon classificationAnimaliaHymenopteraChrysididae

Mocsáry, 1889

Plate 10

Chrysis (Olochrysis) chalcophana Mocsáry 1889: 213.

##### Type locality.

“Caucaus (Coll. Rad.)”.

##### Holotype

♂ [box 60]: golden rounded label // ♂ sp. // Cauca Mlokos [printed] // 116 [printed] // *chalcophana* Mocs. [handwritten by Radoszkowski].

##### Remarks.

The type is badly damaged missing the head, pronotum, fore-legs, and some tarsi of the hind-legs. It is closely related to *Chrysis tenella* Mocsáry, 1889; the main difference is the shape of the pits in the pit-row of the third tergite. Mocsáry (1889) described the two species mainly based on the colouration. Since the body colouration and the pits in the pit-row may be variable, *Chrysis chalcophana* could be synonym of *Chrysis tenella*. It belongs to the *Chrysis millenaris* group.

**Plate 10.**
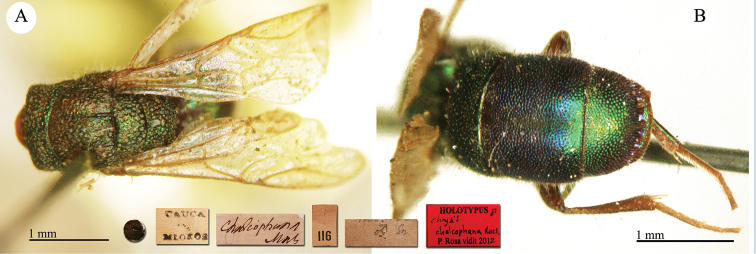
*Chrysis chalcophana* Mocsáry, 1889, holotype. **A** Mesosoma, dorsal view **B** metasoma, dorsal view.

##### Current status.

*Chrysis chalcophana* Mocsáry, 1889.

#### Chrysis chevrieri var. orientalis

Taxon classificationAnimaliaHymenopteraChrysididae

Mocsáry, 1889

Chrysis (Tetrachrysis) Chevrieri var. orientalis Mocsáry 1889: 480, *nec* Guérin-Méneville, 1842.

##### Type locality.

“Patria: Græcia (Parnassus, Coll. Schmiedeknecthi! Ephesus, Mus. Turicense!) et Caucasus (Coll. Rad.! Mus. Hung. et Vindob.! et Coll. Fairmaieri!)”.

##### Paralectotype

1♂ [box 61]: Caucas Mlok [printed] // 263 [handwritten by Mocsáry] // var. *orientalis* Mocs [handwritten by Radoszkowski].

##### Remarks.

Twelve other specimens bearing the same locality labels, but without handwritten identification labels by Radoszkowski, could be considered as paralectotypes. The lectotype was designated by Moczár (1965: 174) at HNHM. Linsenmaier (1959: 149) replaced the name *orientalis* Mocsáry 1889 with *orientica* (comparata ssp. orientica) and considered it as the greenish oriental subspecies of *Chrysis comparata* Lepeletier, 1806. It belongs to the *Chrysis comparata* group.

##### Current status.

*Chrysis comparata orientica* Lepeletier, 1959.

#### Chrysis chlorochrysa

Taxon classificationAnimaliaHymenopteraChrysididae

Mocsáry, 1889

Plate 11

Chrysis (Tetrachrysis) chlorochrysa Mocsáry (Inédite) (in Radoszkowski) 1889 [*nec* 1883]: 23.

##### Type locality.

“Askhabad”.

##### Syntype

1♂ [box 61]: golden rounded label // Ashabad [printed] [yellow label] // *chlorochrysa* Mocs. [handwritten by Radoszkowski] // Rad. [handwritten by Mocsáry] // 39 [handwritten] // 127 [printed] // label with genitalia.

##### Remarks.

Du Buysson (in André) (1895: 500) considered *Chrysis subcoerula* as the female of *chlorochrysa*, but without synonymizing it (*Obs. - Le female décrit par M. le général O. Radoszkowsky appartient à la* Chrysis chlorochrysa *Mocs., d’après le spécimen que l’auteur a eu l’amabilité de m’envoyer.*). One female from Saraks, probably not a type, is housed in MNHN. It belongs to the *Chrysis viridissima* group *sensu* Linsenmaier.

**Plate 11.**
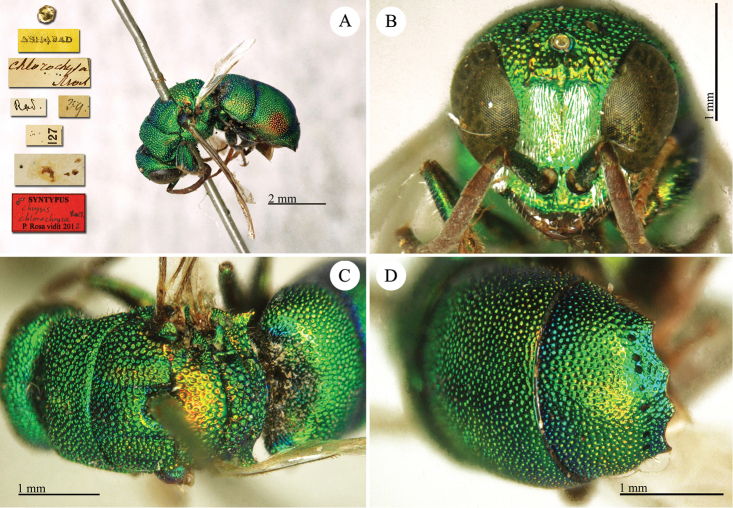
*Chrysis chlorochrysa* Mocsáry, 1889, syntype. **A** Habitus, dorso-lateral view **B** head, frontal view **C** mesosoma, dorsal view **D** third metasomal tergite, dorsal view.

##### Current status.

*Chrysis chlorochrysa* Mocsáry, 1889.

#### Chrysis chrysochlora

Taxon classificationAnimaliaHymenopteraChrysididae

Mocsáry, 1889

Plate 12

Chrysis (Tetrachrysis) chrysochlora Mocsáry 1889: 515.

##### Type locality.

“Patria: Turkestania (Taschkend, Coll. Rad.! et Mus. Hung.)”.

##### Paralectotypes

6♀♀ [box 61]: all specimens bear label Tachkend [printed]; two specimens bear a golden rounded label, one of them bears also other two labels: “*chrysochlora* Mocs” [handwritten by Radoszkowski], “5.” and “126” [printed]; other two specimens bear unreadable label [handwritten]; one specimen bears a label Kapaxymь [handwritten].

##### Remarks.

Bohart (in Kimsey and Bohart 1991: 396) designated a female collected at Tashkent in HNHM as the lectotype. After type examination, we found that *Chrysis chrysochlora* is the female of *Chrysis keriensis* Radoszkowski, 1887.

The name *Chrysis chrysochlora* is commonly found in collections because Linsenmaier (1959: 161) included Chrysis chrysochlora and the subspecies korbiana Mocsáry, 1912 in his revision of the European species. In recent years only Tarbinsky (2000: 193) used *Chrysis chrysochlora* as a valid name in the key of the *Chrysis ignita* group of Tian-Shan. Nevertheless, there is no reason to ask for the reversal of precedence (Art. 23.9 of the Code) and we propose the new synonym *Chrysis chrysochlora* Mocsáry, 1889 = *Chrysis keriensis* Radoszkowski, 1887. It belongs to the *Chrysis ignita* group.

**Plate 12.**
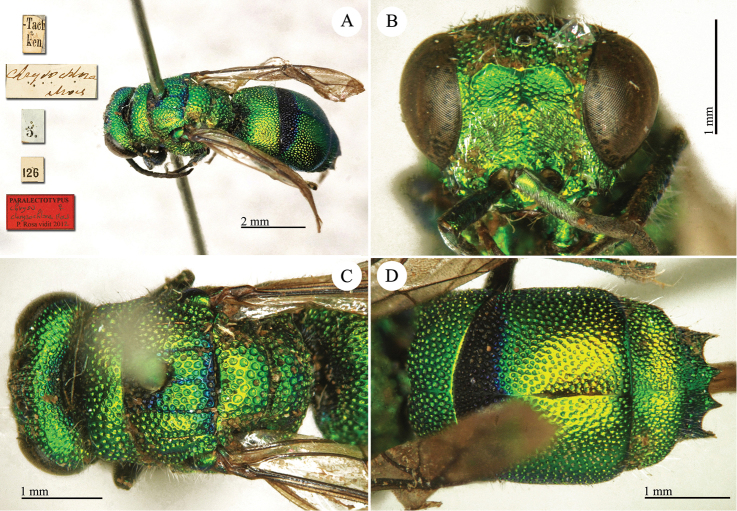
*Chrysis chrysochlora* Mocsáry, 1889, paralectotype. **A** Habitus, dorso-lateral view **B** head, frontal view **C** mesosoma, dorsal view **D** metasoma, dorsal view.

##### Current status.

*Chrysis keriensis* Radoszkowski, 1887.

#### Chrysis circe

Taxon classificationAnimaliaHymenopteraChrysididae

Mocsáry, 1889

Chrysis (Olochrysis) Circe Mocsáry 1889: 230.

##### Type locality.

“Patria: Caucasus (Coll. Rad.)”.

##### Syntype

1♀ [box 60]: label with glued metasoma // Caucas Mlok [printed] // *Phryne* ab. [handwritten by Radoszkowski] // 216 [printed] // *circe* Moc. [handwritten by Radoszkowski].

##### Syntype

1♀ [box 60]: Caucas Nlokos [printed sic!] // *candens* [handwritten by du Buysson] [light blue label] // dark blue rounded label // 103 [printed] // *Chrysis Circe* Mocs. [handwritten by Mocsáry].

##### Remarks.

*Chrysis circe* belongs to the *Chrysis phryne* group.

##### Current status.

*Chrysis circe* Mocsáry, 1889.

#### Chrysis consobrina

Taxon classificationAnimaliaHymenopteraChrysididae

Mocsáry, 1889

Chrysis (Tetrachrysis) consobrina Mocsáry 1889: 458.

##### Type locality.

“Patria: territorium Transcaspicum (Coll. Rad.!) et Persia (Demalen (sic) et Ashabad, Coll. Rad.! et Mus. Hung.)”.

##### Paralectotype

1♀ [box 61]: golden rounded label // Pers Mlok [printed] [orange label] // Demabend [handwritten by Radoszkowski] // 120 [printed] // *consobrina* Mocs. (*prodima* Mocs. i.l. *nec* Cam.) [handwritten by Mocsáry].

##### Paralectotype

1♀ [box 61]: Trans-Caspia [printed] [yellow label] // *consobrina* Mocs. [handwritten by Mocsáry].

##### Paralectotype

1♂ [box 61]: Trans-Caspia [printed] [yellow label] // label with genitalia // *rubescens* ♂ [handwritten by Radoszkowski] // *consobrina* Mocs. [handwritten by Mocsáry].

##### Remarks.

Bohart (in Bohart and French 1986: 341) designated a female collected in Transcaspia and housed at HNHM as the lectotype, and it was later placed in the *Chrysis scutellaris* group in synonymy with *Chrysis soror* (Kimsey and Bohart 1991: 464). Bohart’s lectotype belongs to another species group: the *Chrysis maculicornis* group *sensu* Kimsey and Bohart (1991) or *Chrysis cerastes* group *sensu* Linsenmaier (1959, 1968), being similar to *Chrysis annulata* du Buysson and related species. Rosa et al. (2014) revalidated the species. A revision of this group is needed to clarify the position of various taxa, included *Chrysis consobrina*.

##### Current status.

*Chrysis consobrina* Mocsáry, 1889.

#### Chrysis consobrina var. nova

Taxon classificationAnimaliaHymenopteraChrysididae

Radoszkowski, 1891

Chrysis consobrina var. nova Radoszkowski 1891a: 185.

##### Type locality.

“Ashabad”.

##### Syntypes

1♂1♀ [box 61]: Trans-Caspia.

##### Remarks.

As in other cases of taxa described in 1891 (e.g. *Chrysis simulatrix* and *Chrysis unica*), the specimens considered as syntypes bear the generic locality label “Trans-Caspia” and not “Ashabad”. A female syntype is housed in HNHM and bears the following labels: Trans-Caspia / consobrina var. nova <handwritten by Radoszkowski> / Chrysis scutellaris v. nova Rad. det. Mocsáry / id nr. 115649 HNHM Hym. coll. It was described as a variation of *Chrysis consobrina*, and it matches with the paralectotypes of *Chrysis consobrina* in the Radoszkowski collection. It belongs to the *Chrysis scutellaris* group and it is closely related to *Chrysis soror* Dahlbom, 1854.

##### Current status.

*Chrysis maracandensis* Radoszkowski, 1877 (synonymised by Kimsey and Bohart 1991: 436).

#### Chrysis cylindrica

Taxon classificationAnimaliaHymenopteraChrysididae

Eversmann, 1857

Figure 2

Chrysis cylindrica Eversmann 1857: 554.

##### Type locality.

“in provincia Casanensi” [given in the introduction].

##### Holotype

♀ [box 61]: golden rounded label // Saratow [handwritten] // Chrysis n. sp. cylindrica Evm. [handwritten by Eversmann] // 12 [printed] // *viridula* [handwritten by Radoszkowski].

##### Remarks.

The type is seriously damaged: it lacks metasoma, tibia and tarsi of the fore- and the hind-legs, the right mid-leg, and tarsi of the left mid-leg. It matches Linsenmaier’s interpretation of the species (1968: 81) and it is not a synonym of *Chrysis viridula* Linnaeus, 1761 as stated by Mocsáry (1887: 14). It belongs to the *Chrysis viridula* group.

**Figure 2.**
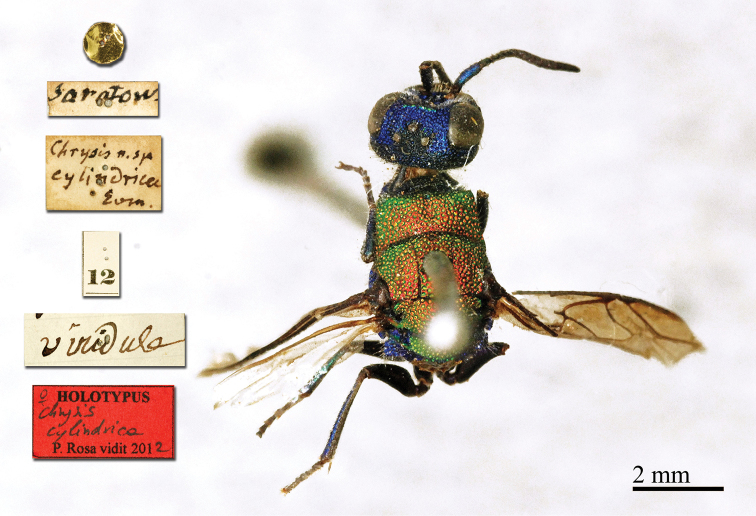
*Chrysis cylindrica* Eversmann, 1857, holotype, head and mesosoma, dorsal view.

##### Current status.

*Chrysis cylindrica* Eversmann, 1857.

#### Chrysis daphnis

Taxon classificationAnimaliaHymenopteraChrysididae

Mocsáry, 1889

Plate 13

Chrysis (Gonochrysis) Daphnis Mocsáry (Inédite) (in Radoszkowski) 1889: 17.

##### Type locality.

“Sicile”.

##### Lectotype

♂ (here designated) [box 60]: golden rounded label // label with genital capsula // Favorita [Palermo] 5-82 [handwritten].

##### Paralectotype

1♂ [box 60]: golden rounded label // I. Sicilia. [printed] // 154 [printed] // *Daphnis* Mocs [handwritten by Radoszkowski].

Kimsey and Bohart (1991: 401) considered *Chrysis daphnis* as a synonym of *Chrysis cylindrica* Eversmann, 1857, while Linsenmaier (1959, 1968, 1997) interpreted *Chrysis daphnis* as a valid species, providing keys and descriptions. Linsenmaier’s interpretation was correct and *Chrysis daphnis* is a valid species strictly related to *Chrysis consanguinea* Mocsáry. Mocsáry (1889) described *Chrysis consanguinea* based on two females (not male and female) from Sicily and Algeria. The two syntypes, examined and housed in MHNG, belong to two different species: *Chrysis daphnis* and *Chrysis consanguinea*. Therefore two lectotype designations are needed to place order in this group. We here designate the lectotype based on the specimen selected by Radoszkowski in his revision of the genital capsulae (1889). The paralectotype is damaged: the head lacks the antennae (except the left scapus) and it is glued on the mesosoma; it lacks the right metatibia and tarsi. The lectotype designation of *Chrysis consanguinea* will be given in a subsequent paper. It belongs to the *Chrysis viridula* group.

**Plate 13.**
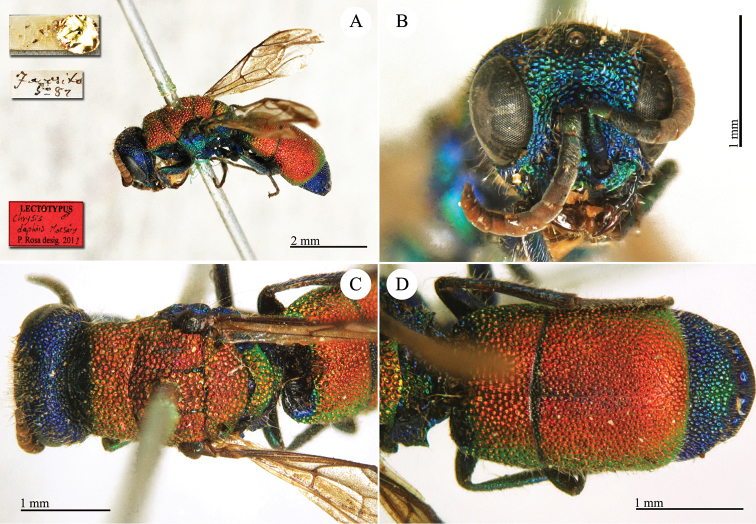
*Chrysis daphnis* Mocsáry, 1889, lectotype. **A** Habitus, dorso-lateral view **B** head, frontal view **C** mesosoma, dorsal view **D** metasoma, dorsal view.

##### Current status.

*Chrysis daphnis* Mocsáry, 1889.

#### Chrysis demavendae

Taxon classificationAnimaliaHymenopteraChrysididae

Radoczkowsky, 1881

Plate 14

Chrysis Demavendae Radoczkowsky 1881: v.

##### Type locality.

“Persia, mons Demavend”.

##### Holotype

♂ [box 62]: golden rounded label // Pers Mlok [printed] [orange label] // label with genitalia // Demabend [handwritten] // 67 [handwritten].

##### Remarks.

Radoszkowski (1889: 33) emended the species name to *Chrysis demabendae* from the name of Mt. Demabend. *Chrysis demabendae* must be considered as an invalid emendation for *Chrysis demavendae* Radoczkowsky, 1881 according to the Art. 32.5.1 of the Code. The species is closely related to *Chrysis sexdentata* Christ, 1791. It belongs to the *Chrysis smaragdula* group *sensu* Kimsey and Bohart (1991).

**Plate 14.**
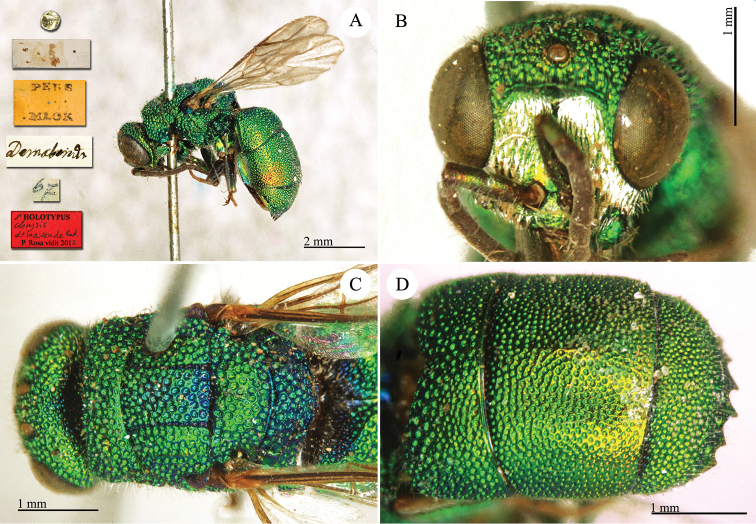
*Chrysis demavendae* Radoczkowsky, 1881, holotype. **A** Habitus, lateral view **B** head, frontal view **C** mesosoma, dorsal view **D** metasoma, dorso-lateral view.

##### Current status.

*Chrysis demavendae* Radoczkowsky, 1881.

#### Chrysis dentipes

Taxon classificationAnimaliaHymenopteraChrysididae

Radoszkowski, 1877

Plate 15

Chrysis dentipes Radoszkowski 1877: 15.

##### Type locality.

“Habitat in valle Sarafshan”, “Пойманъ 8 и 10 мая 1869 г. въ Катты-курганѣ и Заравшанской долинѣ” [collected on 8^th^ and 10^th^ of May 1869 in Katty-Kurgan and in the Zaravshan Valley]. The locality Katty-Kurgan [= Kattakurgan] is in Uzbekistan.

##### Paralectotype

1♀ [box 61]: golden rounded label // Верхн. Заравш. [printed] // 8. [printed] [pink label] // *dentipes* [handwritten by Radoszkowski] // 43 [printed] // *Chrysis dentipes* Rad. [handwritten by Mocsáry].

##### Remarks.

Bohart (in Kimsey and Bohart 1991: 403) designated the lectotype on a female collected at Zaravshan and housed in MMU. It belongs to the *Chrysis taczanovskii* group.

**Plate 15.**
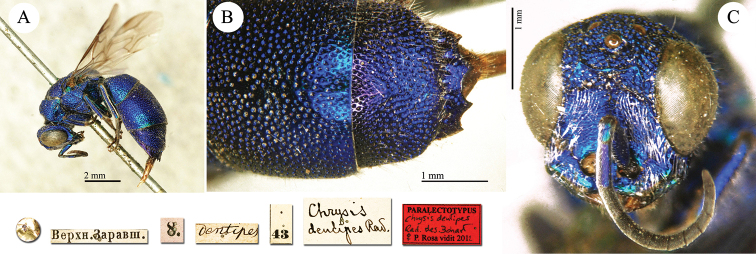
*Chrysis dentipes* Radoszkowski, 1877, paralectotype. **A** Habitus, lateral view **B** metasoma, dorsal view **C** head, frontal view.

##### Current status.

*Chrysis dentipes* Radoszkowski, 1877.

#### Chrysis diademata

Taxon classificationAnimaliaHymenopteraChrysididae

Mocsáry, 1889

Chrysis (Tetrachrysis) diademata Mocsáry 1889: 414.

##### Type locality.

“Patria: Insulæ Philipinæ (Coll. Rad.)”.

##### Holotype

♂ [box 61]: golden rounded label, Mindanao [handwritten] // Brasilia. [printed] [green label] // *diademata* Mocs [handwritten by Radoszkowski] // 108 [printed].

##### Remarks.

One of the labels bears the locality Brasilia. Mocsáry himself noted that the locality Mindanao should be related to the Philippine Islands and not to a Brazilian locality.

The particular red colour of the head is quite typical for species distributed in the islands of the Oriental Region. It belongs to the *Chrysis angolensis* group.

##### Current status.

*Chrysis diademata* Mocsáry, 1889.

#### Chrysis dournovii

Taxon classificationAnimaliaHymenopteraChrysididae

Radoszkovsky, 1866

Plate 16

Chrysis Dournovii Radoszkovsky 1866: 303.

##### Type locality.

“Caucase”.

##### Holotype

♀ [box 60]: golden rounded label // Daghest. [printed] // *Dournovy* [handwritten by du Buysson] // 51 [printed] // Durnovy [handwritten by Radoszkowski].

##### Remarks.

The name *dournovii* was often incorrectly written in different papers and monographs. Some examples: *dournovi* (du Buysson (in André) 1893: 246 sub *Spinolia*; Kimsey and Bohart 1991: 551, sub *Spinolia*); *dournowii* (Dalla Torre 1892: 57 sub *Chrysis*); *durnovi* (Mocsáry 1889: 285 sub Chrysis (Olochrysis); Semenov 1892: 491 sub *Pseudochrysis*; Trautmann 1927: 88 sub *Spinolia*; Linsenmaier 1959: 69 sub Euchroeus (Spinolia)).

**Plate 16.**
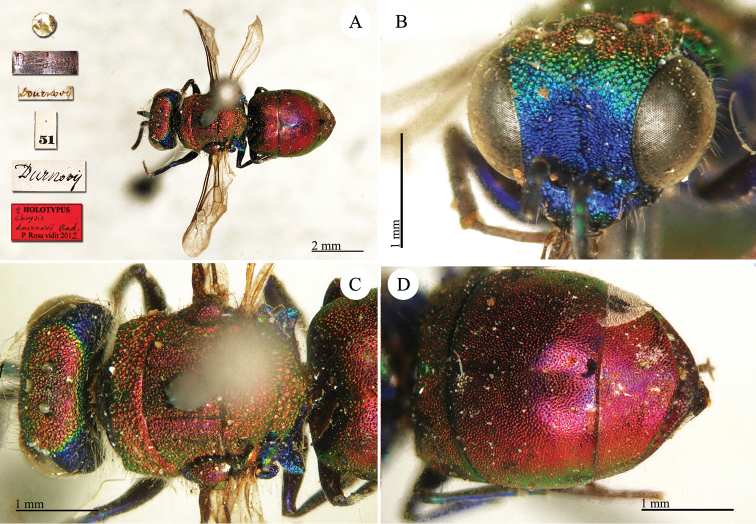
*Chrysis dournovii* Radoszkovsky, 1866, holotype. **A** Habitus, dorsal view **B** head, frontal view **C** mesosoma, dorsal view **D** metasoma, dorsal view.

##### Current status.

*Spinolia dournovii* (Radoszkovsky, 1866) (transferred by du Buysson (in André) 1891: 246).

#### Chrysis dubia

Taxon classificationAnimaliaHymenopteraChrysididae

Radoszkowsky, 1877

Plate 17

Chrysis dubia Radoszkowsky 1877 (1876): 148 *nec* Rossi, 1790.

##### Type locality.

“Apporté par M. Raffray d’Abyssinie”.

##### Holotype

♀ [box 61]: golden rounded label // Abyss. Raffray [printed] [light blue label] // 60 [printed] // *dubia* [handwritten by Radoszkowski] // *Chrysis aethiopica* mihi (*dubia* Rad. *nec* Cress.) [handwritten by Mocsáry].

##### Remarks.

It lacks eight flagellomeres on the left antenna and three flagellomeres on the right one. It belongs to the *Chrysis ignita* group.

**Plate 17.**
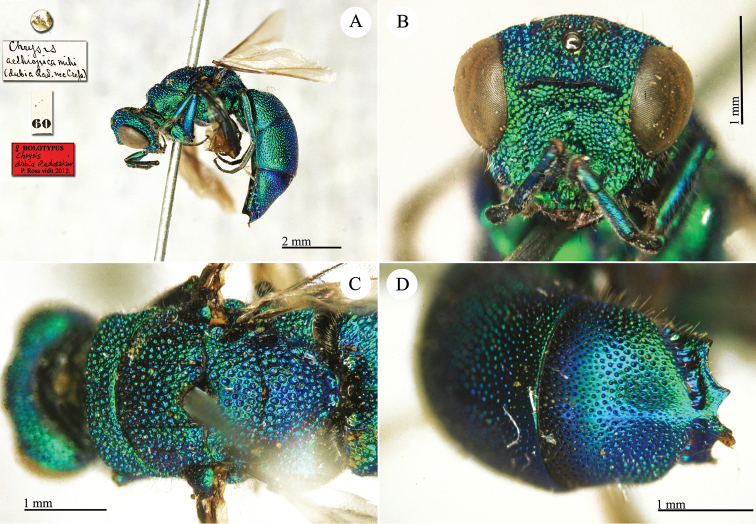
*Chrysis dubia* Radoszkowsky, 1877, holotype. **A** Habitus, lateral view **B** head, frontal view **C** mesosoma, dorsal view **D** third metasomal tergite, dorso-lateral view.

##### Current status.

*Chrysis aethiopica* Mocsáry, 1889, replacement name for *Chrysis dubia* Radoszkovsky, 1877.

#### Chrysis erigone

Taxon classificationAnimaliaHymenopteraChrysididae

Mocsáry, 1889

Chrysis (Olochrysis) Erigone Mocsáry 1889: 239.

##### Type locality.

“Caucasus (Coll. Rad.! et Mus. Hung.)”.

##### Paralectotype

1♀ [box 60]: Caucas Nlokos [printed, sic] // 270 [handwritten by Mocsáry] // *Chrysis urrainensis* Rad [?] [handwritten by Mocsáry].

##### Remarks.

Bohart (in Kimsey and Bohart 1991: 489) designated the lectotype in HNHM. It belongs to the *Chrysis radians* group.

##### Current status.

*Chrysura erigone* (Mocsáry, 1889) (transferred by Kimsey and Bohart 1991).

#### Chrysis erivanensis

Taxon classificationAnimaliaHymenopteraChrysididae

Radoszkovsky, 1880

Plate 18

Chrysis Erivanensis Radoszkovsky 1880 (1879): 146.

##### Type locality.

“Caucase” [written in the introduction].

##### Syntype

1♂ [box 62]: label with genitalia // Erivan [handwritten by Radoszkowski] // 89 [handwritten].

##### Syntype

1♂ [box 62]: golden rounded label // *erivanensi* [handwritten by Radoszkowski] // Erivan [handwritten by Radoszkowski] // 45.

##### Possible Syntype

1♂ [box 62]: *erivanensis* [handwritten by Radoszkowski] // Kasbek [handwritten by Radoszkowski].

##### Remarks.

The two syntypes are badly damaged. Kimsey and Bohart (1991: 408), without type examination, placed it in the *Chrysis smaragdula* group because Radoszkowski described *Chrysis erivanensis* in the section: “*Ano sex-dentatae*”. Radoszkowski described the anal margin of *Chrysis erivanensis* as follows: “*troisième segment finement variolo-chagriné, sa base bleuâtre; points de la serie profonds, inégales; les quatre dents interieures egales élancées; les dents latérales éloignées, remontant vers la base du segment, très peu accentué*”. The anal margin of *Chrysis erivanensis* has four teeth and two lateral rounded swellings, which cannot be considered as true teeth. Even if the the apical margin of the third tergite is unusual, this species can be included in the *Chrysis ignita* group for all the other characteristics.

**Plate 18.**
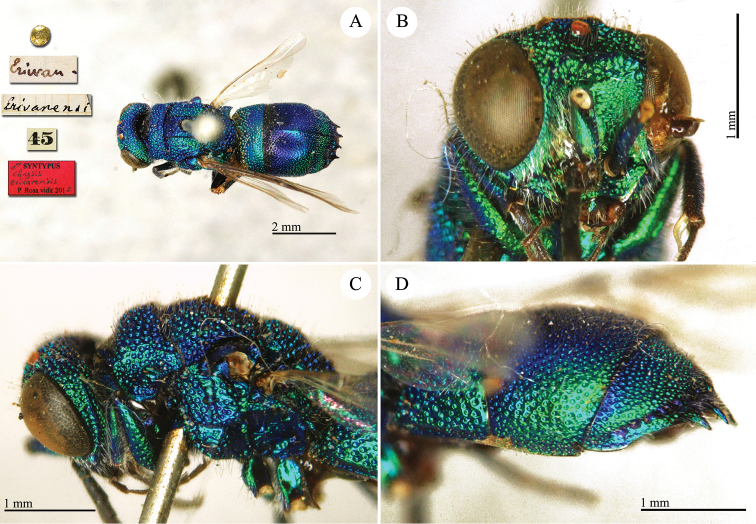
*Chrysis erivanensis* Radoszkovsky, 1880, syntype. **A** Habitus, dorsal view **B** head, frontal view **C** mesosoma, lateral view **D** metasoma, lateral view.

##### Current status.

*Chrysis erivanensis* Radoszkovsky, 1880.

#### Chrysis excisa

Taxon classificationAnimaliaHymenopteraChrysididae

Mocsáry, 1889

Chrysis (Tetrachrysis) excisa Mocsáry (in Radoszkowski) 1889: 25.

##### Type locality.

“France”.

##### Holotype

(?) ♂ [box 61]: 311 20 [handwritten] // 69 [printed] // label with genitalia.

##### Remarks.

We consider the name *Chrysis excisa* as a replacement name for *Chrysis chevrieri* Abeille, *nec* Mocsáry. Nevertheless, many authors, from Dalla Torre (1892: 59) to Kimsey and Bohart (1991: 409), considered *Chrysis excisa* as a new species and not a replacement name. If the second interpretation is correct, the male bearing the dissected genitalia could be considered as the holotype, because Radoszkowski drew and described only the male genitalia. Two females without locality labels, but with handwritten name by Radoszkowski, could be considered as part of the type series, but they were not mentioned in the description. They bear the following labels: first specimen: 267 2 [handwritten] // *excisa* Moc *Chevrieri* Ab. [handwritten by Radoszkowski]; second specimen: 267 7 [handwritten].

Radoszkowski (1889: figs 52, 53, 55) in his collection dissected three specimens with similar colour and habitus, belonging to the *comparata* group: one from France (identified as *Chrysis excisa*), one from Orenbourg (*Chrysis analis*), and one from Caucasus (*Chrysis perrinii*). He did not consider that *Chrysis analis* was described on specimen collected in Liguria (bordering France) and not from specimens collected in central Russia (Orenbourg on the Ural River). Consequently, Radoszkowski mistakenly identified the Russian specimens as *Chrysis analis*, and therefore the French specimen as different species based on the very different genital capsula. Mocsáry described this species based only on Radoszkowski’s drawings. However, the shape of the genital capsula of *Chrysis excisa* Mocsáry is clearly the same of *Chrysis analis* Spinola, and the examination of the types confirm this synonym. Trautmann (1927: 171) and Linsenmaier (1951: 105) already considered *Chrysis excisa* as synonym of *Chrysis analis*, while Kimsey and Bohart (1991: 405) listed *Chrysis excisa* as a valid name. We here confirm the synonym *Chrysis excisa* Mocsáry, 1889 = *Chrysis analis* Spinola, 1808. It belongs to the *Chrysis comparata* group.

##### Current status.

*Chrysis analis* Spinola, 1808 (synonymised by Trautmann 1927: 171).

#### Chrysis exigua

Taxon classificationAnimaliaHymenopteraChrysididae

Mocsáry, 1889

Chrysis (Tetrachrysis) exigua Mocsáry 1889: 414.

##### Type locality.

“Patria: Turkestania (Taschkend, Coll. Rad.)”.

##### Holotype

♀ [box 61]: golden rounded label // Tachkend [printed] // *exigua* Moc [handwritten by Radoszkowski] // 22 [printed].

##### Remarks.

*Chrysis exigua* belongs to the *Chrysis cerastes* group.

##### Current status.

*Chrysis distincta* Mocsáry, 1887 (synonymised by Linsenmaier 1968: 109).

#### Chrysis foveata

Taxon classificationAnimaliaHymenopteraChrysididae

Radoszkowski, 1877

Chrysis foveata Radoszkowski 1877: 13 *nec* Dahlbom, 1845.

##### Type locality.

“Habitat in valle Sarafschan et ad Maracanda [=Samarkand]”, “Bидъ египетскій; пойманъ 12 мая въ Заравшанской долинѣ и 17 іюня 1869 г. въ Самаркандѣ” [Egyptian species; it was collected on the 12^th^ of May in the Zaravshan Valley, and on the 17^th^ of June 1869 at Samarkand].

##### Syntype

1♀ [box 60]: golden rounded label // Урмитанъ [printed] [Urmitan, along the Zarafshan river] // 12 [pink label] // 113 [printed] // *foveata* [handwritten by Radoszkowski] // *foveata* Rad *genalis* Moc [handwritten by Radoszkowski].

##### Remarks.

Radoszkowski (1877: 13) described *Chrysis foveata* (*nec* *foveata* Dahlbom, 1845) based on some syntypes (at least 1 ♂ and 1 ♀ collected at Maracand [currently Samarkand] and in the Zaravshan valley). Later Mocsáry (in Radoszkowski 1889) gave the replacement name *Chrysis genalis*. In the same paper, Radoszkowski (1889: 18; figs 35a, 35b) drew some precise line-drawings of the genital capsule of the male housed in his collection. We do not consider this male as the male syntype, because collected at Tashkent on the 1^st^ of May, day and locality not included in the original description; it bears the labels: golden rounded label // Tachkend [printed] // label with genital capsule // Taшк 1 Maя [handwritten]. Figures of the type and discussions are published in Rosa and Hosseinali (2013). The specimen housed in MMU and considered as holotype by Kimsey and Bohart (1991: 490 sub *Chrysis genalis*) cannot be considered as lectotype by inference according to ICZN (art. 74.5); it bears the labels: Искандеръ [Iskander] / 17 [printed on blue-green label]. It belongs to the *Chrysis radians* group (Rosa and Hosseinali 2013).

##### Current status.

*Chrysura genalis* (Mocsáry, 1887), replacement name for *Chrysis foveata* Radoszkowski, 1877 (transferred by Kimsey and Bohart 1991).

#### Chrysis fulvicornis

Taxon classificationAnimaliaHymenopteraChrysididae

Mocsáry, 1889

Plate 19

Chrysis (Tetrachrysis) fulvicornis Mocsáry 1889: 373.

##### Type locality.

“Patria: Turkestania (Taschkend, Coll. Rad.)”.

##### Holotype

♂ [box 61]: Ϲыръ-Дарья [printed] // golden rounded label // *fulvicornis* Moc [handwritten by Radoszkowski] // 135 [printed] // Chrysis n.sp. fulvicornis Mocs. [handwritten by Mocsáry].

##### Remarks.

The specimen matches the original description. Probably the discrepancy between the locality given in the text [Taschkend] and the one on the label [Syr Daria] is a case of *lapsus calami*. It belongs to the *Chrysis maculicornis* group.

**Plate 19.**
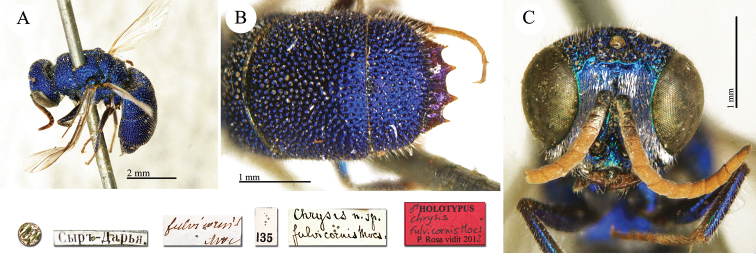
*Chrysis fulvicornis* Mocsáry, 1889, holotype. **A** Habitus, dorso-lateral view **B** second and third metasomal tergites, dorsal view **C** head, frontal view.

##### Current status.

*Chrysis fulvicornis* Mocsáry, 1889.

#### Chrysis gabonensis

Taxon classificationAnimaliaHymenopteraChrysididae

Mocsáry, 1889

Plate 20

Chrysis (Hexachrysis) gabonensis Mocsáry 1889: 384.

##### Type locality.

“Gabon Africæ occidentalis (Coll. Rad.)”.

##### Holotype

♀ [box 62]: golden rounded label // Gabon [handwritten] [green label] // *gabonensis* Moc. [handwritten by Radoszkowski] // 136 [printed].

##### Remarks.

*Chrysis gabonensis* belongs to the *Chrysis smaragdula* group.

**Plate 20.**
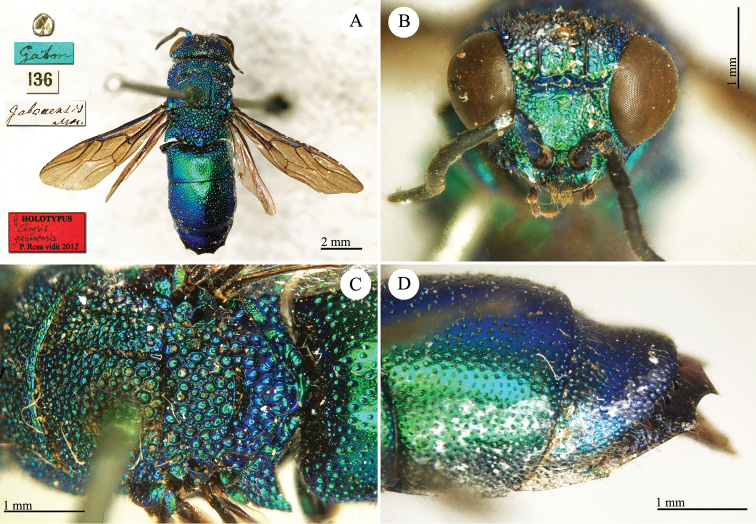
*Chrysis gabonensis* Mocsáry, 1889, holotype. **A** Habitus, dorsal view **B** head, frontal view **C** mesosoma, dorsal view **D** second and third metasomal tergite, lateral view.

##### Current status.

*Chrysis canaliculata* (Brullé, 1846) (synonymised by Kimsey and Bohart 1991).

#### Chrysis gertabi

Taxon classificationAnimaliaHymenopteraChrysididae

Radoszkowski, 1891

Chrysis Gertabi Radoszkowski 1891a: 189.

##### Type locality.

“Ashabad”.

##### Syntype

1♂ [box 61]: golden rounded label // label with genitalia // Trans-Caspia [printed] [yellow label] // *gertabi* ♂ [handwritten by Radoszkowski] // Mus PAN Kraków [handwritten by Dylewska].

##### Remarks.

A syntype male is housed in HNHM and bears the following labels: Ashabad *Gertabi* Rad. n. sp. <handwritten by Radoszkowski and Mocsáry> / Transcapia / Chrysis mutabilis v. Germari (!) Rad det. Mocsáry / id nr. 115619 HNHM Hym. coll. Another syntype is housed in MNHU. It belongs to the *Chrysis cerastes* group.

##### Current status.

*Chrysis mutabilis* du Buysson, 1887 (synonymised by Kimsey and Bohart 1991: 441).

#### Chrysis himalayensis

Taxon classificationAnimaliaHymenopteraChrysididae

Mocsáry, 1889

Plate 21

Chrysis (Pentachrysis) himalayensis Mocsáry (in Radoszkowski) 1889: 31.

##### Type locality.

“Himalaya”.

##### Holotype

♂ [box 62]: golden rounded label // Hymaj [printed] [yellow label] // symbol // label with genitalia.

##### Remarks.

Kimsey and Bohart (1991: 534) synonymised *Chrysis himalayensis* with *Praestochrysis shanghaiensis*. The affinity was already noticed by Radoszkowski (1889: 31). However, the type shows apparent differences in comparison with the male of *Praestochrysis shanghaiensis*. In particular the double TFC, the relative length of antennomeres, the distance between the posterior ocelli, the shape of the metanotal projection, etc. These characteristics confirm that this species could be a valid species.

**Plate 21.**
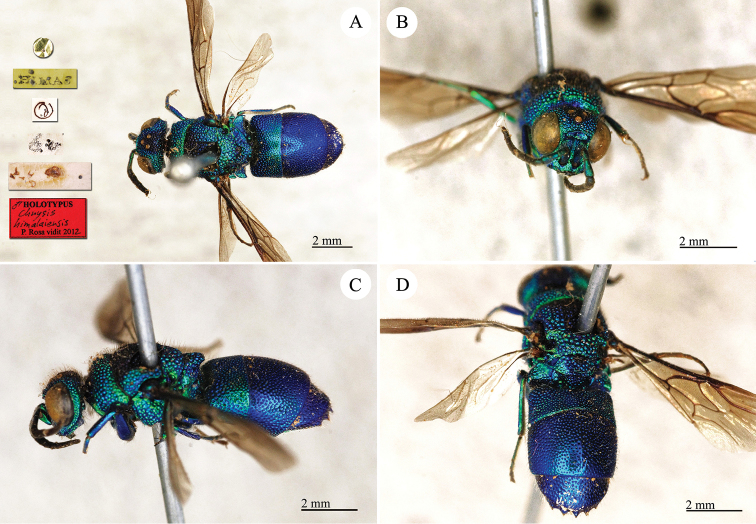
*Chrysis himalayensis* Mocsáry, 1889, holotype. **A** Habitus, dorsal view **B** head, frontal view **C** habitus, lateral view **D** metasoma, dorsal view.

##### Current status.

*Praestochrysis shanghaiensis* (Smith, 1874) (synonymised and transferred by Kimsey and Bohart 1991).

#### Chrysis indigotea

Taxon classificationAnimaliaHymenopteraChrysididae

Dufour & Perris, 1840

Chrysis indigotea Dufour and Perris 1840: 38.

##### Type locality.

France.

**Possible**

##### syntype

1♀ [box 61]: golden rounded label // *indigotea* [handwritten by Radoszkowski] // *typ Dufour* [handwritten by Dufour] // AM [blue label].

##### Remarks.

Syntypes were found in MNHN and other possible syntypes were found in MSNG (Coll. Gribodo) and LZM (Coll. Dahlbom). It belongs to the *Chrysis ignita* group.

##### Current status.

*Chrysis indigotea indigotea* Dufour & Perris, 1840.

#### Chrysis indigotea var. daghestanica

Taxon classificationAnimaliaHymenopteraChrysididae

Mocsáry, 1889

Plate 22

Chrysis (Tetrachrysis) indigotea var. daghestanica Mocsáry 1889: 437.

##### Type locality.

“Patria: Caucasus (Daghestan, Coll. Rad.)”.

##### Holotype

♀ [box 61]: Daghest. [printed] [pink label] // 266 [handwritten by Mocsáry] // Chrysis indigotea var. daghestanica Mocs. [handwritten by Mocsáry].

##### Remarks.

Linsenmaier (1959: 162) considered *Chrysis indigotea daghestanica* as the central Asiatic subspecies of *Chrysis indigotea*. It belongs to the *Chrysis ignita* group.

**Plate 22.**
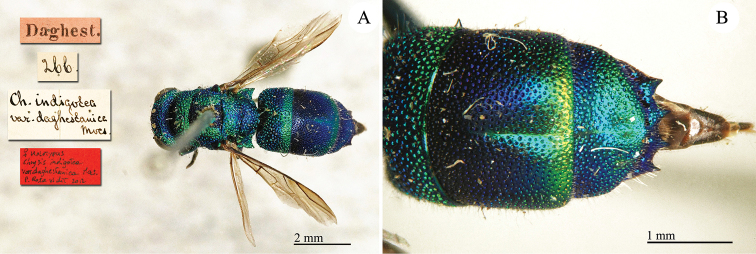
Chrysis indigotea var. daghestanica Mocsáry, 1889, holotype. **A** Habitus, dorsal view **B** metasoma, dorsal view.

##### Current status.

*Chrysis indigotea daghestanica* Mocsáry, 1889 (Linsenmaier 1959).

#### Chrysis jelisyni

Taxon classificationAnimaliaHymenopteraChrysididae

Radoszkowski, 1891

Plate 23

Chrysis Jelisyni Radoszkowski 1891a: 186.

##### Type locality.

“Récoltée par M. Potanin, en Mongolie, (Kansu, Jelissyn-Kuce)”.

##### Syntype

♀ [box 61]: golden rounded label // Kansu Jelisyn-Kuce 20/VII [handwritten] // *Jelisyni* [handwritten by Radoszkowski].

##### Remarks.

Another syntype is preserved in MNHU. It belongs to the *Chrysis comparata* group.

**Plate 23.**
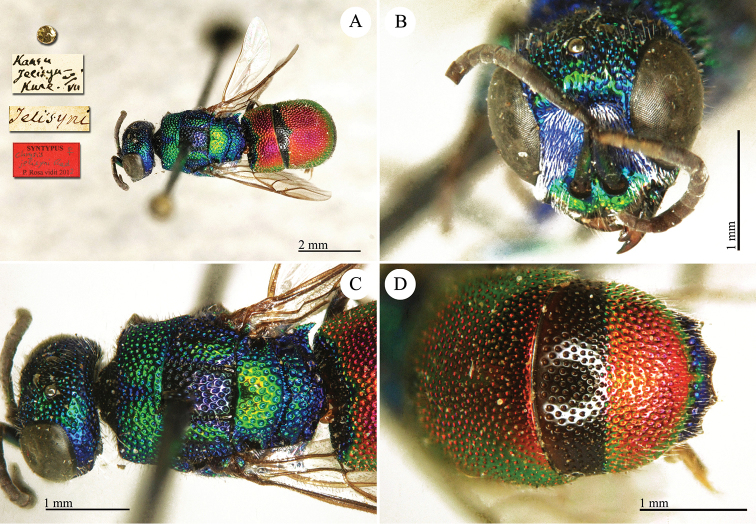
*Chrysis jelisyni* Radoszkowski, 1891, syntype. **A** Habitus, dorsal view **B** head, frontal view **C** mesosoma, dorsal view **D** third metasomal tergite, dorsal view.

##### Current status.

*Chrysis jelisyni* Radoszkowski, 1891.

#### Chrysis keriensis

Taxon classificationAnimaliaHymenopteraChrysididae

Radoszkowski, 1887

Plate 24

Chrysis (Tetrachrysis) keriensis Radoszkowski 1887: 47.

##### Type locality.

“Keria-Daria”.

##### Holotype

♂ [not ♀] [box 61]: golden rounded label // Keria Daria Przewal [printed] [yellow label] // *Kerij* Rad [handwritten by Radoszkowski] // 192 [printed] // *Chrysis Keriensis* M.S.GR T XXI [underlined] p. 47 [handwritten by Radoszkowski].

##### Remarks.

*Chrysis keriensis* Radoszkowski, 1887 is the male of *Chrysis chrysochlora* Mocsáry, 1889. It was treated only by Mocsáry (1889: 516), and listed in checklists by Dalla Torre (1892: 73), Bischoff (1913: 54), Kimsey and Bohart (1991: 427) and Kurzenko and Lelej (2007: 1005). Mocsáry (1889) redescribed the male type of *Chrysis keriensis* immediately after the description of the female of *Chrysis chrysochlora*. The differences observed by Mocsáry (1889) between *Chrysis keriensis* and *Chrysis chrysochlora* are dimorphic sexual dissimilarities.

**Plate 24.**
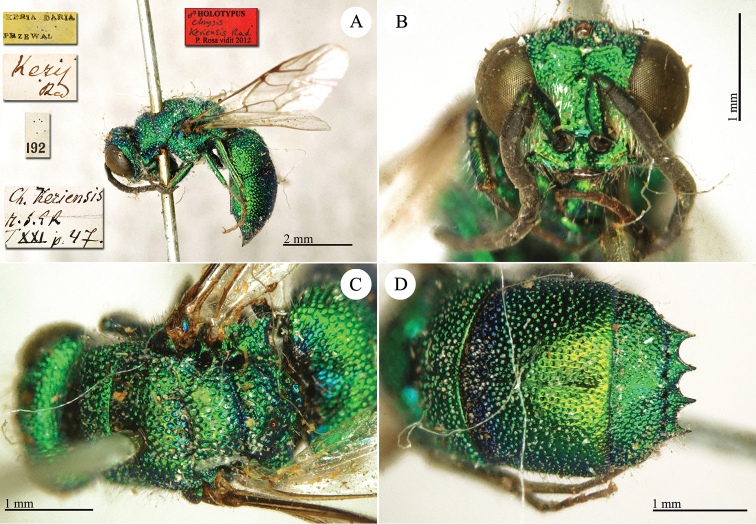
*Chrysis keriensis* Radoszkowski, 1887, holotype. **A** Habitus, lateral view **B** head, frontal view **C** mesosoma, dorsal view **D** third metasomal tergite, dorsal view.

##### Current status.

*Chrysis keriensis* Radoszkowski, 1887.

#### Chrysis komarowi

Taxon classificationAnimaliaHymenopteraChrysididae

Radoszkowski, 1891

Chrysis Komarowi Radoszkowski 1891a: 190.

##### Type locality.

“Ashabad; envoyé par le général Komarow”.

##### Syntype

1♀ [box 61]: golden rounded label // Frans-Caspi G. Turcmenien E. König [sic! Printed] // [small square pink label without any note] // Komarovy [handwritten by Radoszkowski].

##### Syntype

1♂ [box 61]: golden rounded label // Frans-Caspi G. Turcmenien E. König [sic! Printed].

##### Remarks.

There are one male and one female in the collection bearing the same locality label: Frans-Caspi [sic] G. Turcmenien E. König. Both syntypes were collected by König and sent to Radoszkowski by Komarow. Another specimen with the same locality label is deposited in MNHN (general collection box 41). The female is badly damaged. It belongs to the *Chrysis succincta* group.

##### Current status.

*Chrysis komarowi* Radoszkowski, 1891.

#### Chrysis kriechbaumeri

Taxon classificationAnimaliaHymenopteraChrysididae

Gribodo, 1879

Chrysis kriechbaumeri Gribodo 1879: 358.

##### Type locality.

“Hab. in Nova-Hollandia”.

##### Possible Paralectotype

1♀ [box 60]: golden rounded label // *Kriechbaum* [handwritten by Radoszkowski] // Nov. Holl. [printed] // 254 [printed] // label with the metasoma.

##### Possible Paralectotype

1♀ [box 60]: golden rounded label // Nov. Holl. [printed].

##### Remarks.

The specimens are part of the type series described by Gribodo. Bohart (in Kimsey and Bohart 1991: 542) designated the lectotype in Drewsen’s collection in ZMUC. Another paralectotype is housed in MSNG (Rosa 2009: 239). It belongs to the *Primeuchroeus faustus* group.

##### Current status.

*Primeuchroeus kriechbaumeri* (Gribodo, 1879) (transferred by Bohart 1988: 24).

#### Chrysis lagodechii

Taxon classificationAnimaliaHymenopteraChrysididae

Radoszkowski, 1889

Plate 25

Chrysis (Olochrysis) Lagodechii Radoszkowski 1889: 15.

##### Type locality.

“Caucase (Lagodekhi)”.

##### Lectotype

♂ [here designated] [box 60]: Cauca Mlokos [printed] // label with genital capsula // *Lagodechii* [handwritten by Radoszkowski] // 284 [handwritten by Mocsáry] // *angustifrons* [handwritten by Radoszkowski].

##### Paralectotype

1♂ [box 60]: Caucas [printed].

##### Remarks.

Two males and one female collected in Caucasus were found under the name *Chrysis lagodechii* Rad. We consider the two males as types, and we exclude the female bearing the label “Eldar Caucas” [printed], because Radoszkowski did not mention any female in his description. This female specimen belongs to the genus *Chrysura*. Since various species are present under the same name, we here designate the lectotype based on one male of the type series. It belongs to the *Chrysis elegans* group.

**Plate 25.**
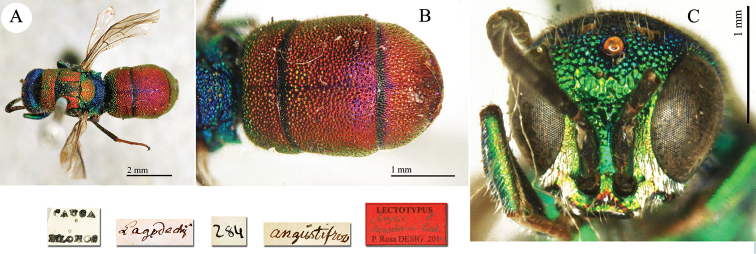
*Chrysis lagodechii* Radoszkowski, 1889, lectotype. **A** Habitus, dorsal view **B** metasoma, dorsal view **C** head, frontal view.

##### Current status.

*Chrysis angustifrons* Abeille de Perrin, 1878 (synonymised by Mocsáry 1889: 274).

#### Chrysis lepida

Taxon classificationAnimaliaHymenopteraChrysididae

Mocsáry, 1889

Figure 3

Chrysis (Olochrysis) lepida Mocsáry 1889: 278.

##### Type locality.

“Patria: Caucasus (Coll. Rad.!, Erivan, Mus. Hung.)”.

##### Paralectotype

1♀ [box 60]: golden rounded label // Caucas Port [printed] [light blue label] // Erevan [?] [handwritten by Radoszkowski] // *Lepida* Mocs [handwritten by Radoszkowski] // 114 [printed].

##### Remarks.

The specimen is partly damaged, and the metasoma is glued to the mesosoma. Mocsáry (1889) described *Chrysis lepida* based on at least two specimens collected at Erivan and preserved in the Radoszkowski collection and in HNHM. Bohart (in Bohart and French 1986: 342) designated the lectotype in HNHM. The lectotype housed in HNHM bears the labels: Kaukasus Erivan / *lepida* Mocs. typ. det. Mocsáry / red label / Holotypus *Chrysis lepida* ♀ Mocs. RM Bohart / id nr. 135152 HNHM Hym. coll. It belongs to the *Chrysis elegans* group.

**Figure 3.**
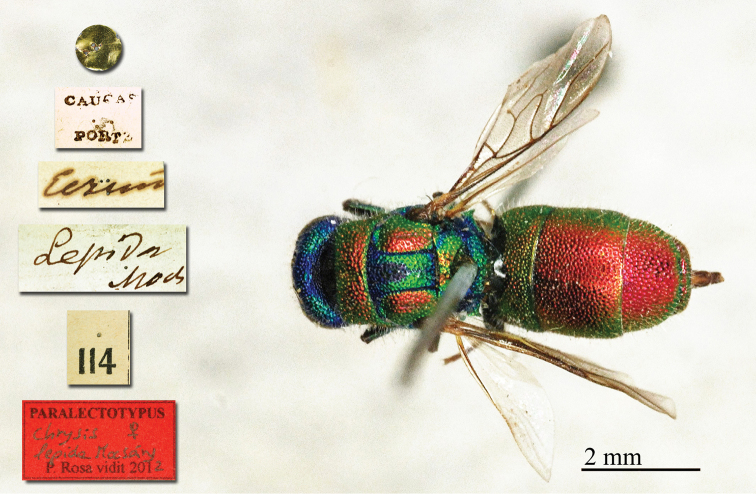
*Chrysis lepida* Mocsáry, 1889, paralectotype, habitus, dorsal view.

##### Current status.

*Chrysis lepida* Mocsáry, 1889.

#### Chrysis luzonica

Taxon classificationAnimaliaHymenopteraChrysididae

Mocsáry, 1889

Chrysis (Trichrysis) luzonica Mocsáry 1889: 328.

##### Type locality.

“Lucon in insulis Philippinis (Coll. Rad.)”.

##### Holotype

♀ [box 61]: Lucon [handwritten] [yellow label] // 275 [handwritten by Mocsáry] // *luzonica* Moc [handwritten by Radoszkowski].

##### Current status.

*Trichrysis luzonica* (Mocsáry, 1889) (transferred by Kimsey and Bohart 1991: 572).

#### Chrysis maracandensis

Taxon classificationAnimaliaHymenopteraChrysididae

Radoszkowski, 1877

Plate 26

Chrysis maracandensis Radoszkowski 1877: 14.

##### Type locality.

“Habitat in valle Sarafschan et in desertis prope Taschkent”, “Пойманъ 2 іюня въ Заравшанской долинѣ, 9 іюня 1869 г. въ Самаркандѣ и 28 мая 1871 г. въ степи между Сыръ-дарьей и Ташкентомъ” [collected on the 2^nd^ of June in the Zaravshan Valley, 9^th^ June 1869 at Samarkand and the 28^th^ of May 1871 in steppe between Syr-Darya and Tashkent].

##### Paralectotype

1♂ [box 61]: golden rounded label // Ташкентъ [printed] 28. [printed] [pink label with red line] // Marakand [handwritten by Radoszkowski] // 14 [handwritten] // label with genitalia.

##### Paralectotype

1♂ [box 61]: golden rounded label // 9. [printed] [blue label] // Ϲамаркандъ // 47 [printed].

##### Remarks.

Lectotype designated by Bohart (in Kimsey and Bohart 1991: 436) based on a male housed in MMU. All the specimens are males and not females as written in the original description. In the Radoszkowski collection there is another specimen collected at Taschkent, also with the golden rounded label, which is not considered as a paralectotype because the locality label is printed in Latin [Tachkend], while all the specimens described in 1877 bear labels printed in Cyrillic. It belongs to the *Chrysis scutellaris* group.

**Plate 26.**
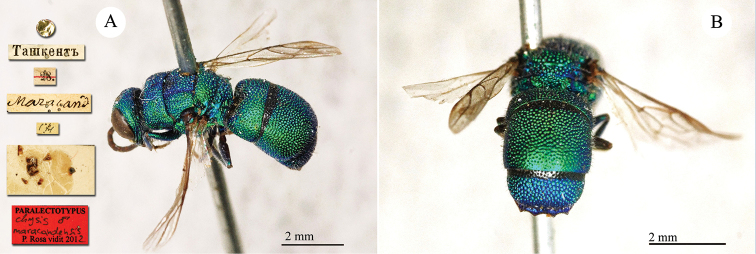
*Chrysis maracandensis* Radoszkowski, 1877, paralectotype. **A** Habitus, dorso-lateral view **B** habitus, dorsal view.

##### Current status.

*Chrysis maracandensis* Radoszkowski, 1877.

#### Chrysis maracandensis var. simulatrix

Taxon classificationAnimaliaHymenopteraChrysididae

Radoszkowski, 1891

Chrysis maracandensis var. simulatrix Radoszkowski 1891a: 185.

##### Type locality.

“Ashabad”.

##### Syntype

1♂: Trans-Caspia [printed] [yellow label] // var. *simulatilis* [handwritten by Radoszkowski].

##### Remarks.

Radoszkowski described Chrysis maracandensis var. simulatrix based on a syntype series. In his collection there are five specimens under the label *simulatilis* (sic). Four of them were collected at Sarakhs, while the fifth was collected in “Trans-Caspia” and bears the label handwritten by Radoszkowski “var. *simulatilis*”. The latter can be considered as a syntype, in the same way of other species described in 1891 and bearing the same locality label. All the specimens belong to *Chrysis maracandensis* Radoszkowski. Therefore the synonym: Chrysis maracandensis var. simulatrix Radoszkowski, 1891 = *Chrysis maracandensis* Radoszkowski, 1877, is here proposed. Another possible syntype is housed in MNHN (general collection box 41). It belongs to the *Chrysis scutellaris* group.

##### Current status.

*Chrysis maracandensis* Radoszkowski, 1877.

#### Chrysis marginata

Taxon classificationAnimaliaHymenopteraChrysididae

Mocsáry, 1889

Plate 27

Chrysis (Tetrachrysis) marginata Mocsáry 1889: 451.

##### Type locality.

“Turkestania (Coll. Rad.)”.

##### Holotype

♀ [box 61]: Верхн. Эаравш. // 8. [printed] [pink label] // *marginata* [handwritten by Radoszkowski] Mocs. [handwritten by Mocsáry] // 252 [printed].

##### Remarks.

The type is partly damaged: it lacks the left antenna, nine flagellomeres of the right one, and the left fore-leg after the coxa. It belongs to the *Chrysis comparata* group.

**Plate 27.**
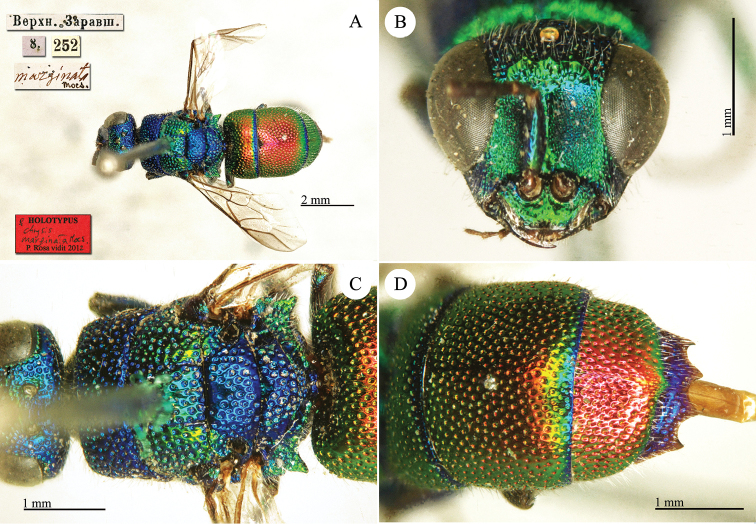
*Chrysis marginata* Mocsáry, 1889, holotype. **A** Habitus, dorsal view **B** head, frontal view **C** mesosoma, dorsal view **D** second and third metasomal tergites, dorsal view.

##### Current status.

*Chrysis marginata* Mocsáry, 1889.

#### Chrysis minutissima

Taxon classificationAnimaliaHymenopteraChrysididae

Radoszkowsky, 1877

Chrysis minutissima Radoszkowsky 1877 (1876): 147.

##### Type locality.

“Egypte et Abyssinie” [written in the introduction].

##### Holotype

♂ [not ♀] [box 61]: golden rounded label // Egypt C. Bra [Comte Branicki] [printed] [blue label] // *minutissim* [handwritten by Radoszkowski] // 54 [printed].

##### Remarks.

The correct locality is Egypt, even if in the original description the locality is not clearly indicated. It belongs to the *Chrysis succincta* group.

##### Current status.

*Chrysis minutissima* Radoszkowsky, 1877.

#### Chrysis mirabilis

Taxon classificationAnimaliaHymenopteraChrysididae

Radoszkovsky, 1877

Chrysis mirabilis Radoszkovsky 1877 (1876): 106.

##### Type locality.

“Envoyée du Caucase par Mr. Mlokosiewitz”.

##### Holotype

♂ [box 61]: golden rounded label // Cauca Mlokos [printed] // 55 [handwritten] // label with genitalia // 50 [printed] // *mirabilis* Rad. [handwritten by Radoszkowski].

##### Remarks.

The type is in bad condition, it lacks antennae, the left forewing, as well as part of the left hind-leg. A possible syntype is housed in MNHU. It belongs to the *Chrysis facialis* group.

##### Current status.

*Chrysis mirabilis* Radoszkovsky, 1877.

#### Chrysis mlokosewitzi

Taxon classificationAnimaliaHymenopteraChrysididae

Radoszkowski, 1889

Plate 28

Chrysis (Olochrysis) Mlokosewitzi Radoszkowski 1889: 13.

##### Type locality.

“Caucase”.

##### Holotype

♂ [not ♀] [box 60]: golden rounded label // Caucasus [printed] // *mlokosewitz* [handwritten by Radoszkowski] [light blue label] // 115 [printed].

##### Remarks.

The holotype is a rufescent specimen belonging to the genus *Chrysura* and the *Chrysis dichroa* group.

**Plate 28.**
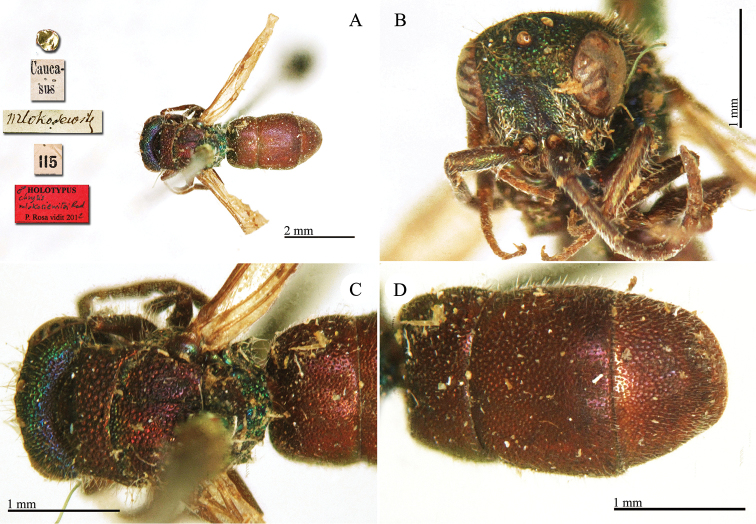
*Chrysis mlokosewitzi* Radoszkowski, 1889, holotype. **A** Habitus, dorsal view **B** head, frontal view **C** mesosoma, dorsal view **D** metasoma, dorso-lateral view.

##### Current status.

*Chrysura mlokosewitzi* (Radoszkowski, 1889), **comb. n.**

#### Chrysis mocsaryi

Taxon classificationAnimaliaHymenopteraChrysididae

Radoszkowski, 1889

Figure 4

Chrysis (Tetrachrysis) Mocsaryi Radoszkowski 1889: 29.

##### Type locality.

“apportée par Mr. Potanin de Mongolie (Kobden [currently Kobdo])”.

##### Holotype

♀ [box 61]: label with right flagellum and metasoma // golden rounded label // Kansu Kobden-Owatu 12/VIII [handwritten] // *Mocsáry* [handwritten by Radoszkowski] // *Chrysis Mocsaryi* Rad. (tres interep.) [?] [handwritten by Mocsáry] // Mus. Pan Krakow [hadwritten by Dylewska].

##### Remarks.

The type is damaged: the right flagellum and the metasoma are glued on a label. It belongs to the *Chrysis comparata* group.

**Figure 4.**
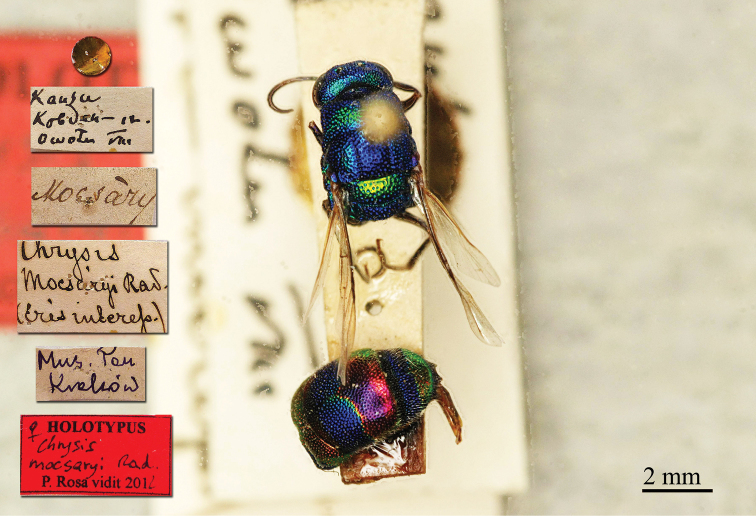
*Chrysis mocsaryi* Radoszkowski, 1889, holotype, head and mesosoma + metasoma, dorsal view.

##### Current status.

*Chrysis mocsaryi* Radoszkowski, 1889.

#### Chrysis murgrabi

Taxon classificationAnimaliaHymenopteraChrysididae

Radoszkowski, 1891

Chrysis Murgrabi Radoszkowski 1891a: 196.

##### Type locality.

“Murgrab”.

##### Syntype

(?) 1♂ [box 61]: golden rounded label // Tr-Cap Saraks // *Murgrabi* [handwritten by Radoszkowski] // Museum PAN Krakow [handwritten by Dylewska].

##### Remarks.

According to Kimsey and Bohart (1991: 435) the syntypes of *Chrysis murgrabi* are preserved in the Radoszkowski collection. The description of *Chrysis murgrabi* was based on one male and one female, but now there is only one male specimen left in the collection. It is badly damaged, the right forewing is missing, and the metasoma was found in the box and glued on a separate label. The locality given on the label (Transcaspia) is not accurate, compared with the locality given in the text (Murgrab = Murgab, currently in Tajikistan), however it cannot be excluded from the syntypes based only the locality, because the specimens described by Radoszkowski in 1891 bear not precise locality labels. Radoszkowski (1893b: 81) emended the name to *Chrysis murgabi*, but this name must be considered as an unjustified emendation according to the Art. 32.5.1 of the Code. It belongs to the *Chrysis maculicornis* group.

##### Current status.

*Chrysis maculicornis* Klug, 1845 (synonymised by Kimsey and Bohart 1991).

#### Chrysis obscura

Taxon classificationAnimaliaHymenopteraChrysididae

Radoszkovsky, 1877

Plate 29

Chrysis obscura Radoszkovsky 1877 (1876): 106.

##### Type locality.

“Envoyée du Caucase par Mr. Mlokosiewitz”.

##### Holotype

♂ [box 61]: golden rounded label // Cauca Mlokos [printed] // 130 [printed] // *obscura* Rad *chalcochrysa* Mocs [handwritten by Radoszkowski] // *undulata* Rad. [handwritten by Mocsáry].

##### Remarks.

Kimsey and Bohart (1991: 461) placed *Chrysis chalcochrysa* in synonymy with *Chrysis scutellaris* Fabricius, without type examination. *Chrysis obscura* belongs to the *Chrysis succincta* group and not to the *Chrysis scutellaris* group. *Chrysis chalcochrysa* is a valid taxon related to the *Chrysis grohmanni* subgroup, with a unique colouration of the mesosoma.

**Plate 29.**
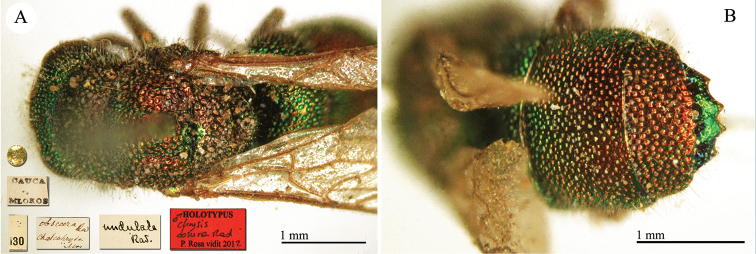
*Chrysis obscura* Radoszkovsky, 1877, holotype. **A** Mesosoma, dorsal view **B** third metasomal tergite, dorsal view.

##### Current status.

*Chrysis chalcochrysa* Mocsáry, 1887, replacement name for *obscura* Radoszkovsky *nec* Smith, 1859, **status revived**.

#### Chrysis octavii

Taxon classificationAnimaliaHymenopteraChrysididae

du Buysson, 1895

Plate 30

Chrysis octavii du Buysson (in André) 1895: 476.

##### Type locality.

“Égypte (Radoszkowsky); Sicile”.

##### Syntype

1♀ [box 61]: Taczano [printed] // Egyptus. [printed] [blue label] // *episcopalis* [handwritten by Rad.] // 92 [printed] // Chrysis (Pyria) Octavii Buyss. n.sp. [handwritten by du Buysson] [orange label].

##### Remarks.

The type is badly damaged, missing the compound eyes, some legs and the ventral surface (including the internal segments). The second syntype is housed in MNHN. It belongs to the *Chrysis taczanovskii* group.

**Plate 30.**
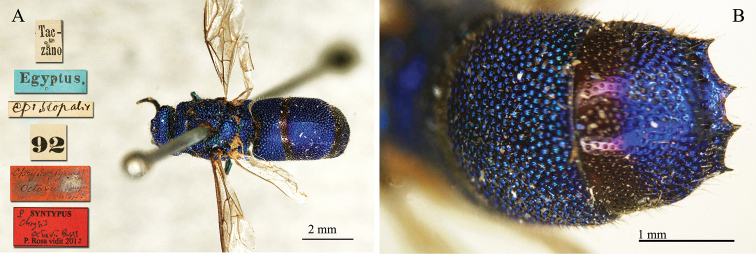
*Chrysis octavii* du Buysson, 1895, syntype. **A** Habitus, dorsal view **B** third metasomal tergite, dorsal view.

##### Current status.

*Chrysis chlorospila* Klug, 1845 (synonymised by Bischoff 1913: 49).

#### Chrysis oraniensis var. portentosae

Taxon classificationAnimaliaHymenopteraChrysididae

Radoszkowski, 1891

Chrysis oraniensis var. portentosae Radoszkowski 1891a: 184.

##### Type locality.

“Je possède un exemplaire provenant d’Amasis”.

##### Holotype

♀ [box 60]: Amasis [handwritten] // Algeria [printed] [blue label] // orian var. portentosa [handwritten by Radoszkowski].

##### Remarks.

Under the locality label ‘Amasis’ there is a second locality label: Algeria. The species was described based on one specimen collected at Amasis (not “Atrek”, in Kimsey and Bohart 1991: 493). Algeria is the locality of the nominal species. It belongs to the *Chrysis cuprea* group.

##### Current status.

*Chrysura oraniensis* (Lucas, 1849) (synonymised by Trautmann 1927: 117; transferred by Kimsey and Bohart 1991: 493).

#### Chrysis patriarchalis

Taxon classificationAnimaliaHymenopteraChrysididae

Radoszkovsky, 1880

Chrysis patriarchalis Radoszkovsky 1880 (1879): 142.

##### Type locality.

“Etschmiadzine”.

##### Syntype

1♂ [box 60]: golden rounded label // Erivan [handwritten] // 10 [printed] // *Patriarchalis* [handwritten].

##### Remarks.

The description of *Chrysis patriarchalis* was based on syntypes. The type locality Etschmiadzine (= Etchmiadzin), currently Vagharshapat, Armenia, is close to Erivan.

##### Current status.

*Spintharina versicolor* (Spinola, 1808) (synonymised by Mocsáry 1887: 15; transferred by Kimsey and Bohart 1991: 558).

#### Chrysis persica

Taxon classificationAnimaliaHymenopteraChrysididae

Radoczkowsky, 1881

Plate 31

Chrysis persica Radoczkowsky 1881: v.

##### Type locality.

“Persia, mons Demavend”.

##### Lectotype

♀ (here designed) [box 60]: golden rounded label // Pers Mlok [printed] [orange label] // Demabend [handwritten by Radoszkowski] // *Chrysogona pumila* Klug (*assimilis* Dhlb.) [handwritten by Mocsáry] // *Chrysis Persica* exempl [...]gate typique, on a decrib d’apres cet exemplars [handwritten, partly unreadable].

##### Paralectotype

1♀ [box 60]: golden rounded label // Pers Mlok [printed] [orange label] // Demabend [handwritten by Radoszkowski] // 53 [printed] // *persica* [handwritten by Radoszkowski] // *Chrysogona pumila* Klug (*assimilis* Spin.) [handwritten by Mocsary].

##### Remarks.

After Mocsáry’s monograph (1889: 183), *Chrysis persica* was always considered as a synonym of *Chrysogona pumila* Klug. Linsenmaier (1959: 171) revalidated the taxon at first, but after a few years he changed his interpretation and placed *Chrysis persica* again in synonymy with *Chrysogona pumila* (Linsenmaier 1987: 155). Since one of the two syntypes is seriously damaged, we here designate the lectotype of *Chrysis persica* and confirm that it is synonym of *Chrysogona pumila* Klug, 1845 (= *Chrysogona pumila* *sensu* Linsenmaier (1987)); the case is discussed in detail in Rosa and Xu (2015). The lectotype lacks some flagellomeres (3–11) from the left antenna, some tarsi on the left fore-leg; head and propleurae are partially separated from the rest of the body.

**Plate 31.**
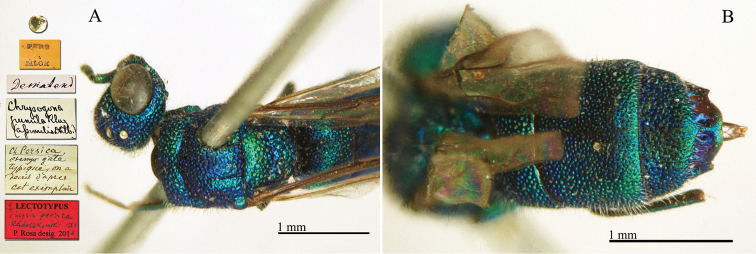
*Chrysis persica* Radoczkowsky, 1881, lectotype. **A** Mesosoma, dorsal view **B** second and third metasomal tergites, dorsal view.

##### Current status.

*Chrysidea pumila* (Klug, 1845) (synonymised by Linsenmaier 1987; transferred by Kimsey and Bohart 1991).

#### Chrysis poecilochroa

Taxon classificationAnimaliaHymenopteraChrysididae

Mocsáry, 1889

Plate 32

Chrysis (Tetrachrysis) poecilochroa Mocsáry (Inedité) (in Radoszkowski) 1889: 27.

##### Type locality.

“Algérie”.

##### Holotype

♂ [box 61]: golden rounded label // Algeria [printed] [blue label] // *poecilochroa* Mocs [handwritten by Radoszkowski] // 123 [printed] // label with genitalia.

##### Remarks.

Linsenmaier (1968: 110) considered *Chrysis poecilochroa* the northern African subspecies of *Chrysis distincta* Mocsáry. Kimsey and Bohart (1991: 405) considered *Chrysis poecilochroa* as a synonym of *Chrysis distincta* Mocsáry, 1887. It belongs to the *Chrysis cerastes* group.

**Plate 32.**
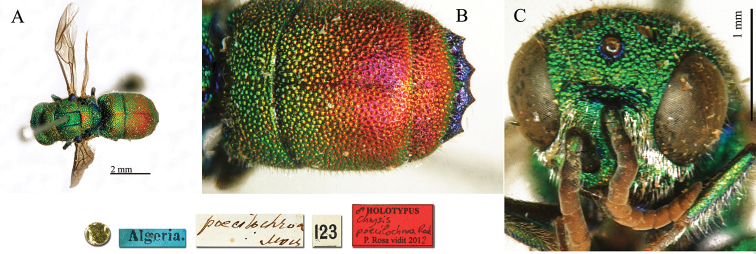
*Chrysis poecilochroa* Mocsáry, 1889, holotype. **A** Habitus, dorsal view **B** metasoma, dorsal view **C** head, frontal view.

##### Current status.

*Chrysis distincta* Mocsáry, 1887 (synonymised by Kimsey and Bohart 1991).

#### Chrysis pomerantzovi

Taxon classificationAnimaliaHymenopteraChrysididae

Radoszkowski, 1891

Plate 33

Chrysis Pomerantzovi Radoszkowski 1891a: 184.

##### Type locality.

“Atrek”.

##### Holotype

♀ [box 60]: Atrek [handwritten] [yellow label] // *Pomerantzovi* [handwritten by Radoszkowski].

##### Remarks.

The type lacks the left flagellum. It belongs to the *Chrysis aestiva* group.

**Plate 33.**
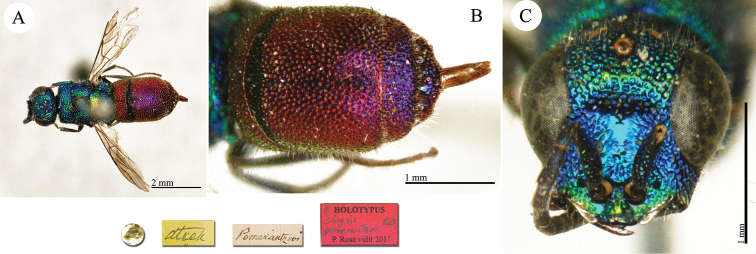
*Chrysis pomerantzovi* Radoszkowski, 1891, holotype. **A** Habitus, dorsal view **B** metasoma, dorsal view **C** Head, frontal view.

##### Current status.

*Chrysis pomerantzovi* Radoszkowski, 1891.

#### Chrysis potanini

Taxon classificationAnimaliaHymenopteraChrysididae

Radoszkowski, 1891

Figure 5

Chrysis Potanini Radoszkowski 1891a: 186.

##### Type locality.

“Récoltée par M. Potanin en Mongolie (Tufyn)”.

##### Holotype

♂ [box 61]: golden rounded label // *potanini* [handwritten by Radoszkowski] // Mongol. mer. Tufyn 11/VII [handwritten] // label with genitalia // Mus. PAN Krakow [handwritten by Dylewska].

##### Remarks.

The type lacks the right flagellum and some tarsi on the left hind-leg. It belongs to the *Chrysis comparata* group.

**Figure 5.**
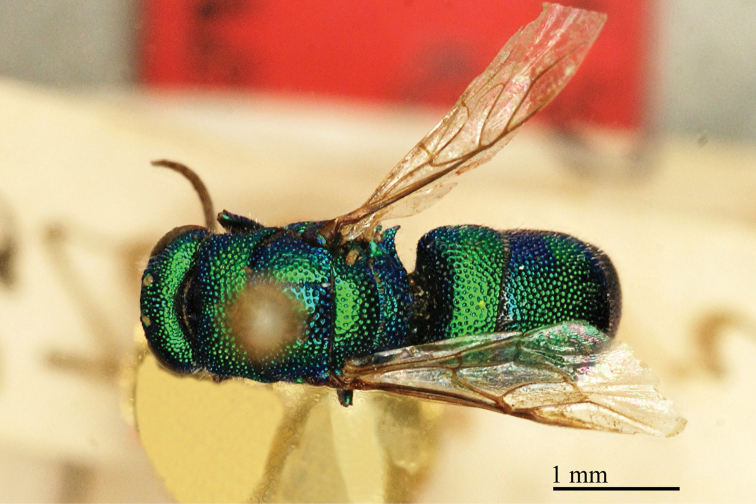
*Chrysis potanini* Radoszkowski, 1891, holotype, habitus, dorsal view.

##### Current status.

*Chrysis potanini* Radoszkowski, 1891.

#### Chrysis przewalskii

Taxon classificationAnimaliaHymenopteraChrysididae

Radoszkowski, 1887

Plate 34

Chrysis (Tetrachrysis) Przewalskii Radoszkowski 1887: 46.

##### Type locality.

“Zaïdam, les chaines des montagnes Keria (9000’)”.

##### Holotype

♂ [box 61]: label with the metasoma // golden rounded label // Caidom Przewal [printed] [yellow label] // n.sp *Przewalski* [handwritten by Radoszkowski] // 191 [printed].

##### Remarks.

The type is damaged, missing of the right hind-leg and some tarsi from the left mid- and hind-legs; the metasoma is glued on a label. It is included in the *Chrysis pulchella* group (Kimsey and Bohart 1991: 452).

**Plate 34.**
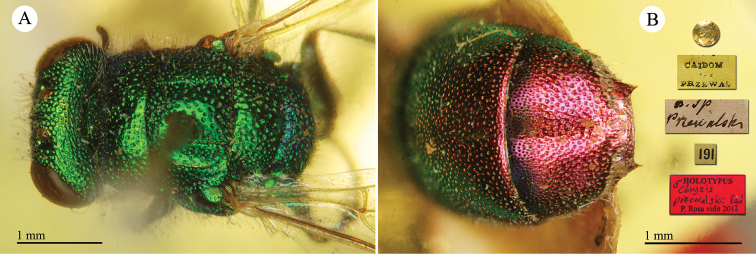
*Chrysis przewalskii* Radoszkowski, 1887, holotype. **A** Head and mesosoma, dorsal view **B** third metasomal tergite, dorsal view.

##### Current status.

*Chrysis przewalskii* Radoszkowski, 1887.

#### Chrysis pulchra

Taxon classificationAnimaliaHymenopteraChrysididae

Radoszkovsky, 1880

Figure 6

Chrysis pulchra Radoszkovsky 1880 (1879): 143.

##### Type locality.

“Caucase” [written in the introduction].

##### Holotype

♀ (not ♂) [box 62]: golden rounded label // Cauca Mlokos [printed] // 9 [printed] // *pulchra* [handwritten by Radoszkowski] // Spinolia magnifica Dah pulchra Rad [handwritten by Radoszkowski].

##### Remarks.

*Chrysis pulchra* Radoszkovsky, 1880 and *Chrysis sulcata* Radoszkovsky, 1866 *nec* Dahlbom, 1845 were synonymised with *Chrysis lamprosoma* Förster, 1853 [currently *Spinolia*] by Mocsáry (1887: 16). Few years later Mocsáry (1896: 2) described Chrysis (Spinolia) dallatorreana based on the specimens housed in HNHM.

*Spinolia dallatorreana* is now found to be a synonym of *Spinolia pulchra* Radoszkovsky, 1880. However, *Spinolia dallatorreana* is currently in use after Mocsáry’s monograph (1889), even if Kimsey and Bohart (1991: 551) placed it in synonymy with *Spinolia insignis* (Lucas, 1849). Only two authors accepted the synonym: Mingo (1994: 225) and Tyrner (2007: 49; (in Macek et al.) 2010: 66). Kimsey (1986: 106) designated the lectotype of *Spinolia dallatorreana* in MNHN, but Móczár (1964b: 448) already designated the lectotype, which is housed in HNHM and was checked.

In order to preserve the nomenclatural stability, we propose the reversal of precedence (Art. 23.9 of the Code) and we consider *Chrysis pulchra* as **nomen oblitum** and *Chrysis dallatorreana* as **nomen protectum.** According to Code, the reversal of precedence can be applied only when the two following conditions are both met: when the senior synonym has not been used as a valid name after 1899 (Art. 23.9.1.1) and when the the junior synonym has been used in at least 25 works, published by at least 10 authors in the immediately preceding 50 years and encompassing a span of not less than 10 years (23.9.1.2).

In this case, *Spinolia pulchra* was never used again as a valid species name after 1887. Unfortunately, only 16 works citing *Spinolia dallatorreana* were published in the last 50 years (excluding other three papers dated from 1954 to 1959); on the other hand, at least 10 authors considered *dallatorreana* as a valid name: Kimsey (1983: 145; 1986: 106); Linsenmaier (1968: 41; 1969: 354; 1987: 144; 1997: 261; 1999: 96; sub Euchroeus (Spinolia)); Mingo (1975: 135 sub Euchroeus (Spinolia)); Móczár (1964b: 448; 1967: 62); Negru (1965: 198); Rosa (2005: 36; 2006b: 92); Schmidt (1977: 107); Strumia and Yildirim (2009: 85); Wiśniowski and Strumia (2007: 81). Other three authors listed and described *Spinolia dallatorreana*, but after the period of 50 years: Haupt (1956: 121), Linsenmaier (1959: 69) and Zimmermann (1954: 5). Since the conditions are not met, we apply to the Art. 23.9.3. of the Code: if the conditions of 23.9.1 are not met but nevertheless an author considers that the use of the older synonym or homonym would threaten stability or universality or cause confusion, and so wishes to maintain use of the junior synonym, he must refer the matter to the Commission for a ruling under the plenary power [Art. 81]. While the case is under consideration use of the junior name is to be maintained [Art. 82]. A paper with all the cases found in other museums will be soon forwarded to the ICZN. Meanwhile the name *Spinolia dallatorreana* must be maintained.

**Figure 6.**
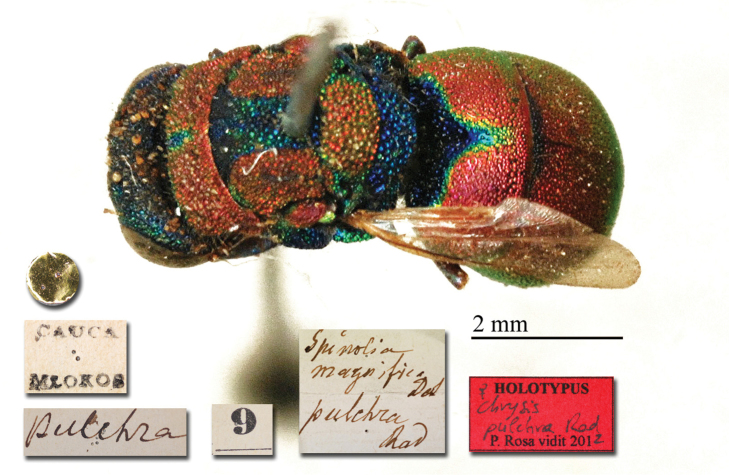
*Chrysis pulchra* Radoszkovsky, 1880, holotype, habitus, dorsal view.

##### Current status.

*Spinolia dallatorreana* (Mocsáry, 1896), **nomen protectum**.

#### Chrysis remota

Taxon classificationAnimaliaHymenopteraChrysididae

Mocsáry, 1889

Plate 35

Chrysis (Tetrachrysis) remota Mocsáry (in Radoszkowski) 1889: 21.

##### Type locality.

“Patria: Demabend (in Persia) et Caucasus (a Domino Mlokosewitz detecta)”.

##### Lectotype

♂ (here designed) [box 61]: golden rounded label // Pers Mlok [printed] [orange label] // Demabend [handwritten] // *Remota* Mocs [handwritten by Radoszkowski] // 129 [printed].

##### Paralectotype

1♂ [box 61]; Caucas Mlokos [printed] // label with genitalia.

##### Remarks.

According to interpretation of the species by Linsenmaier (1968) and Kimsey and Bohart (1991), *Chrysis remota* belongs to the *Chrysis graelsii* group. The lectotype designation is necessary because the syntypes belong to two different species. The specimen selected as lectotype belongs to the *Chrysis graelsii* group, while the paralectotype to the *Chrysis maculicornis* group.

**Plate 35.**
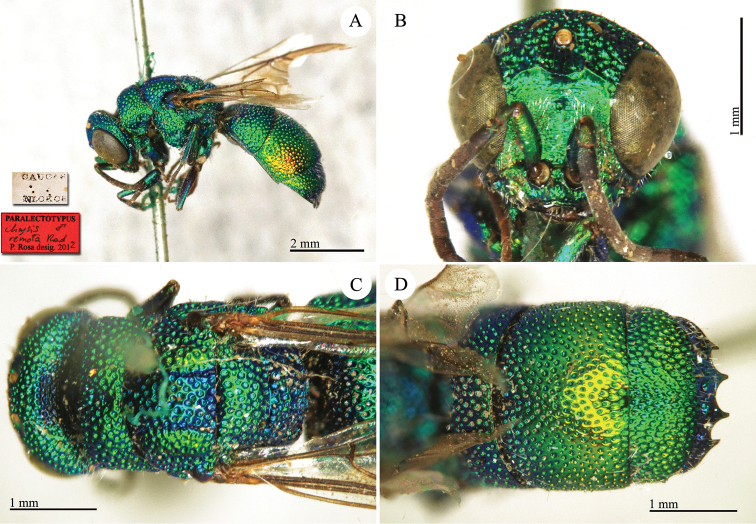
*Chrysis remota* Mocsáry, 1889, paralectotype. **A** Habitus, lateral view **B** head, frontal view **C** mesosoma, dorsal view **D** metasoma, dorsal view.

##### Current status.

*Chrysis remota* Mocsáry, 1889.

#### Chrysis rutilans var. asiatica

Taxon classificationAnimaliaHymenopteraChrysididae

Mocsáry, 1889

Chrysis (Tetrachrysis) rutilans var. Asiatica Mocsáry 1889: 448 *nec* Radoszkowski, 1889.

##### Type locality.

“Turkestania, Taschkend (Coll. Rad.)”.

##### Syntype

1♀ [box 61]: Tachkend [printed] // 214 [printed] // var. *asiatica* Mocs. [handwritten by Radoszkowski] // *splendidula* Dlb [handwritten by Radoszkowski].

##### Syntype

1♀ [box 61]: label with two legs and the metasoma // Ashabad [printed] [yellow label] // 244 [printed] // var. *asiati*. [handwritten by Radoszkowski].

##### Remarks.

Semenov-Tian-Shanskij (1912) replaced the name in Chrysis insperata ssp. mesasiatica. Another possible syntype is housed in MNHN (general collection box 48). It belongs to the *Chrysis splendidula* group.

##### Current status.

*Chrysis decora* Mocsáry, 1889 (replacement name for *Chrysis superba* Radoszkowski, 1877) (synonymised by Kimsey and Bohart 1991: 402).

#### Chrysis sabulosa

Taxon classificationAnimaliaHymenopteraChrysididae

Radoszkowski, 1877

Chrysis sabulosa Radoszkowski 1877: 24.

##### Type locality.

“Habitat in monte Karak”, “Три ♂ этого вида пойманы 7 мая 1871 г. нa горѣ Каракъ” [Three males of this species were collected on the 7^th^ of May 1871 on the Karak mountain].

##### Syntype

1♀ [not a male!] [box 62]: golden rounded label // *sabulosa* [handwritten by Radoszkowski] // 7. [printed] [pink label with red line] // Каракь [printed] // 118 [printed] // label with metasoma.

##### Remarks.

The type is seriously damaged: it lacks the left antenna and the right flagellum, the mid- and hind-legs; the face is partially covered by glue; the prothorax is glued to the mesothorax; the metasoma is glued on a separate label.

In the description, Radoszkowski listed only three males, but the picture of the species (table II, picture 11) undoubtedly shows a female with an exserted ovipositor. Another syntype is found in MMU and it was considered as the holotype by Kimsey and Bohart (1991), it bears the following labels, 7. [pink label with red line] / Каракъ [printed] / *Chrysis sabulosa* Rad. <handwritten red label>. According to Kimsey and Bohart (1991) it belongs to the *Chrysis sabulosa* group.

##### Current status.

*Chrysis sabulosa* Radoszkowski, 1877.

#### Chrysis sarafschana

Taxon classificationAnimaliaHymenopteraChrysididae

Mocsáry, 1889

Plate 36

Chrysis (Tetrachrysis) sarafschana Mocsáry 1889: 437.

##### Type locality.

“Turkestania (vallis Sarafschan, Coll. Rad.)”.

##### Holotype

♀ [box 61]: golden rounded label // Верхн. Заравш. // *ulianini* [handwritten by Radoszkowski] // 27. [printed] [pink label] // 52 [printed] // *sarafschana* Mocs. [handwritten by Mocsáry].

##### Remarks.

The type is seriously damaged, without metasoma and some flagellomeres of antennae. This specimen is also the second syntype of *Chrysis uljanini* Radoszkowski, 1877.

Mocsáry (1889) described *Chrysis sarafschana* based on the female syntype of *Chrysis uljanini* received by Radoszkowski. In his diagnosis, Mocsáry explained the reasons why *Chrysis sarafschana* [belonging to the *Chrysis ignita* group] cannot be the female of *Chrysis uljanini* [belonging to the *Chrysis cerastes* group]. This interpretation was later followed by other authors: Radoszkowki (1889: tab. 51, the drawing of the genital capsule is not related to the species belonging to the *Chrysis ignita* group), Dalla Torre (1892: 92, 104), du Buysson (in André) (1895: 506, 512), Bischoff (1910: 58, 61), Tsuneki (1953: 27) and Linsenmaier (1959: 159). For further remarks see under *Chrysis uljanini* Radoszkowski and *Chrysis uljanini* Radoszkowski & Mocsáry.

**Plate 36.**
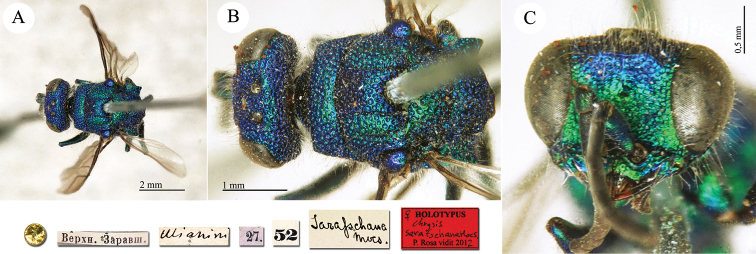
*Chrysis sarafschana* Mocsáry, 1889, holotype. **A** Head and mesosoma, dorsal view **B** head and mesosoma, dorsal view **C** head, frontal view.

##### Current status.

*Chrysis uljanini* Radoszkowski, 1877 (synonymized by Kimsey and Bohart 1991).

#### Chrysis saraksensis

Taxon classificationAnimaliaHymenopteraChrysididae

Radoszkowski, 1891

Chrysis saraksensis Radoszkowski, 1891a: 195.

##### Type locality.

“Saraks”.

##### Holotype

♂ [box 61]: golden rounded label // Tr-Cap Saraks [printed] [yellow label with metasoma glued to it] // *Saraksensis* [handwritten by Radoszkowski].

##### Remarks.

The type is in bad condition: it lacks the left flagellum, both fore wings, as well as mid- and hind-legs. The metasoma was found on the bottom of the box and glued on the locality label.

Radoszkowski emended the name *Chrysis saraksensis* to *Chrysis seraxensis* (Radoszkowski 1893b: 81), without any comment. The name *Chrysis seraxensis* was later used by du Buysson ((in Andrè) 1896: 728), Bischoff (1913: 59) and Semenov-Tian-Shanskij and Nikol’skaya (1954: 128). The emendation is unjustified according to the Art. 32.5.1 of the Code: incorrect transliteration or latinization, or use of an inappropriate connecting vowel, are not to be considered inadvertent errors. The name *Chrysis saraksensis* is the incorrect transliteration of a locality name written in Arabic. Kimsey and Bohart (1991: 428) placed *Chrysis saraksensis* in synonymy with *Chrysis kokandica* Radoszkowski in the *Chrysis splendidula* group. Linsenmaier (1994: 197) revalidated *Chrysis sarakensis* and placed it in the *Chrysis cerastes* group. *Chrysis saraxensis* belongs to the *Chrysis cerastes* group and cannot be a synonym of *Chrysis kokandica* Radoszkowski because it belongs to the *Chrysis splendidula* group.

##### Current status.

*Chrysis saraksensis* Radoszkowski, 1891.

#### Chrysis sardarica

Taxon classificationAnimaliaHymenopteraChrysididae

Radoszkowski, 1890

Chrysis sardarica Radoszkowski 1890: 509.

##### Type locality.

“Ararat, entre Sardar-Abadu et Sarabandy (13,000’)” [given in the introduction].

##### Holotype

♂ [box 61]: golden rounded label // Ararat [printed] [yellow label] // *sardarica* R. [handwritten by Radoszkowski].

##### Remarks.

The type is seriously damaged: it lacks the metasoma. Moreover, dermestid damage caused the loss of compound eyes, part of the occiput, left antenna, right flagellum, and both fore-legs. The specimen is pinned, and the pin has broken the mesothorax. It belongs to the *Chrysis aestiva* group.

##### Current status.

*Chrysis sardarica* Radoszkowski, 1890.

#### Chrysis semenovi

Taxon classificationAnimaliaHymenopteraChrysididae

Radoszkowski, 1891

Plate 37

Chrysis Semenovi Radoszkowski 1891a: 193.

##### Type locality.

“Saraks”.

##### Lectotype

♀ [box 61]: golden rounded label // Tr-Cap Saraks [printed] [yellow label] // Semenovyi [handwritten by Radoszkowski] // Mus-PAN Krakow [handwritten by Dylewska] // **Lectotype** ♀ *Chrysis semenovi* Rad. R.M. Bohart [handwritten on red label].

##### Paralectotypes

2♀♀ [box 61]: Tr-Cap Saraks [printed] [yellow label] // Mus. PAN Krakow semenovi [handwritten by Dylewska] // **Paralectotype** ♀ *Chrysis semenovi* Rad. R.M. Bohart [handwritten on red label].

##### Remarks.

Bohart (in Kimsey and Bohart 1991: 461) designated the lectotype and placed *Chrysis semenovi* in the *Chrysis comparata-scutellaris* group. The three specimens labelled by Bohart were found under the label *Chrysis semenovi* R. and belong to two different species. However, the two specimens considered as paralectotypes have not been labelled by Radoszkowski and do not match the original description. On the anterior surface, the colour of the first tergite is blue contrasting with the red colour of the remaining part of the segment. According to the original description “*Abdomen régulièrment scrobiculé feu-doré; premier segment tirant au jaune-doré, les deuxième et troisième plus rouges* [...]”.

The specimen selected as lectotype belongs to the *cerastes* group and not to the *comparata-scutellaris* group. Radoszkowski himself added in his diagnosis: “Voisine de *Chrysis incerta* Rad.”. *Chrysis semenovi* is very close to *Chrysis annulata* Abeille de Perrin (in du Buysson), 1887, and the main characteristics which allows separation of the two species is the shape of the transversal frontal carina. In *Chrysis annulata* there are two branches directed backwards on the vertex, while in *Chrysis semenovi* the transversal frontal carina is simple, without branches on the vertex.

**Plate 37.**
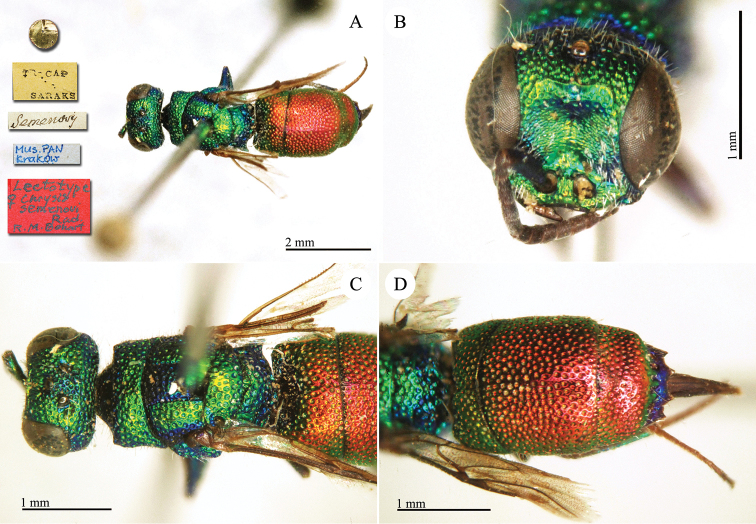
*Chrysis semenovi* Radoszkowski, 1891, lectotype. **A** Habitus, dorsal view **B** head, frontal view **C** head and mesosoma, dorsal view **D** metasoma, dorsal view.

##### Current status.

*Chrysis semenovi* Radoszkowski, 1891.

#### Chrysis separanda

Taxon classificationAnimaliaHymenopteraChrysididae

Mocsáry, 1889

Chrysis (Olochrysis) separanda Mocsáry (Inédite) (in Radoszkowski) 1889: 14.

##### Type locality.

“Syra”.

##### Syntype

1♂ [box 60]: golden rounded label // *separanda* Mocs [handwritten by Radoszkowski] // 51 [handwritten] // Syra [handwritten] // label with genital capsula.

##### Syntype

1♀ [box 60]: golden rounded label // Syra [handwritten] // *separanda* Mocs [handwritten by Radoszkowski] // *Chrysis varicornis* Spin ♀ Syra.

##### Syntypes

2♀♀ [box 60]: Syra [handwritten].

##### Remarks.

*Chrysis separanda* belongs to the *Chrysis radians* group.

##### Current status.

*Chrysura varicornis* (Spinola, 1838) (synonymised and transferred by Kimsey and Bohart 1991: 497).

#### Chrysis serena

Taxon classificationAnimaliaHymenopteraChrysididae

Radoszkowski, 1891

Plate 38

Chrysis serena Radoszkowski 1891a: 194.

##### Type locality.

“Saraks”.

##### Holotype

♂ [box 61]: golden rounded label // Tr-Cap Saraks [printed] [yellow label] // *serena* [handwritten by Radoszkowski].

##### Remarks.

*Chrysis serena* belongs to the *Chrysis viridula* group. The type lacks the right hind-leg, tarsi of the right mid-leg, as well as part of the right flagellum; fore wings are partially ripped.

**Plate 38.**
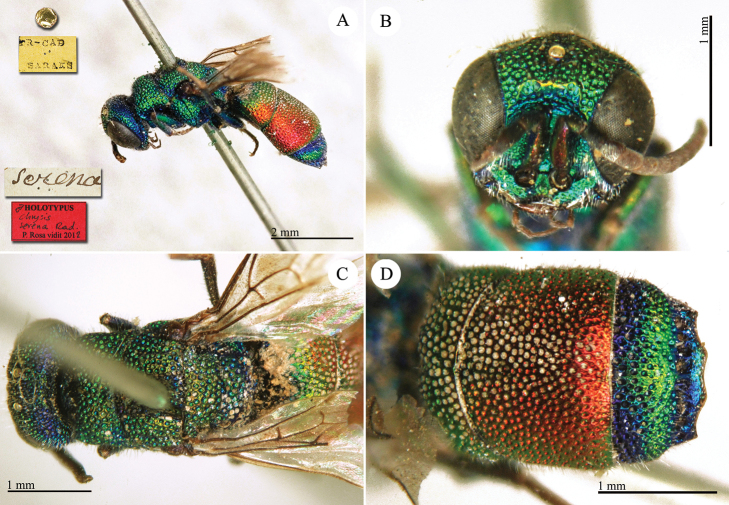
*Chrysis serena* Radoszkowski, 1891, holotype. **A** Habitus, lateral view **B** head, frontal view **C** mesosoma, dorsal view **D** metasoma, dorsal view.

##### Current status.

*Chrysis serena* Radoszkowski, 1891.

#### Chrysis singula

Taxon classificationAnimaliaHymenopteraChrysididae

Radoszkowski, 1891

Plate 39

Chrysis singula Radoszkowski 1891a: 187.

##### Type locality.

“Ashabad”.

##### Syntype

1♀ [box 61]: golden rounded label // yellow rounded label // Trans-Caspia [printed] [yellow label] // *singula* [handwritten by Radoszkowski].

##### Syntypes

2♀♀ [box 61]: yellow rounded label.

##### Remarks.

Radoszkowski described this species based on a syntype series (“7-8 1/3 mill.”). Nowadays in the collection there is only one specimen bearing the locality label and the handwritten label “*singula*” by Radoszkowski. In HNHM there is another female syntype labelled: Astrabad *singula* Rad. n.sp. <handwritten by both Radoszkowski and Mocsáry> / Chrysis grohmanni v. singula Rad. det. Mocsáry / id nr. 115604 HNHM Hym. coll. Another syntype is housed in MNHU and other two possible syntypes are deposited in MNHN (general collection box 54).

Linsenmaier (1959: 109; 1968: 62) used the name Chrysis grohmanni ssp. bolivari Mercet, 1902 (erroneously written *bolivieri*) for the specimens belonging to *Chrysis singula* Radoszkowski; Linsenmaier clearly wrote that he did not know *Chrysis singula* Radoszkowski. Kimsey and Bohart (1991: 416) included *Chrysis singula* in the synonymic list of *Chrysis grohmanni* Dahlbom. *Chrysis grohmanni grohmanni* Dahlbom is limited to the western Europe and North Africa (from Morocco to Tunisia). Various sister species (treated as subspecies by Linsenmaier) occur in eastern Europe, North Africa, Near East to central Asia. It belongs to the *Chrysis succincta* group.

**Plate 39.**
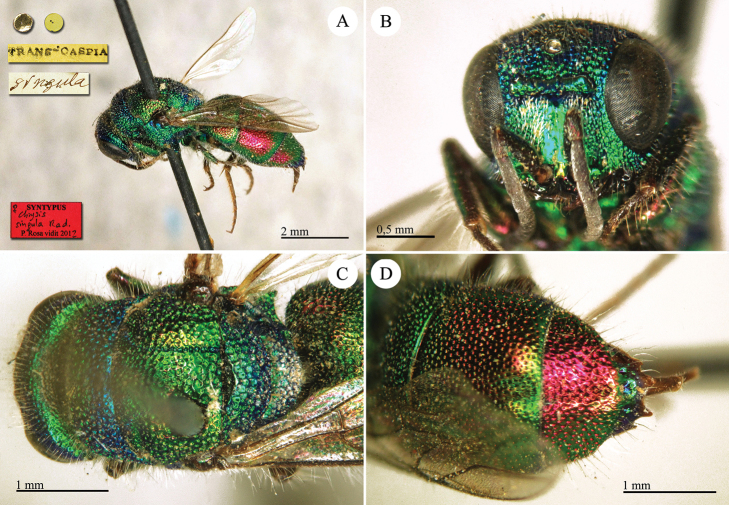
*Chrysis singula* Radoszkowski, 1891, syntype. **A** Habitus, dorso-lateral view **B** head, frontal view **C** mesosoma, dorsal view **D** second and third metasomal tergites, dorso-lateral view.

##### Current status.

*Chrysis singula* Radoszkowski, 1891.

#### Chrysis spinidens

Taxon classificationAnimaliaHymenopteraChrysididae

Mocsáry, 1887

Plate 40

Chrysis (Tetrachrysis) spinidens Mocsáry (inédite) Radoszkowski 1887: 48.

##### Type locality.

“Zaïdam (Mongolia)”.

##### Holotype

♂ [box 61]: golden rounded label // Caidom Przewal [printed] [yellow label] // spinidens Mocs [handwritten by Radoszkowski] // S [handwritten by Radoszkowski] // 125 [printed] 77.

##### Remarks.

*Chrysis spinidens* belongs to the *Chrysis ignita* group. It could be also synonymous with *Chrysis carnifex* Mocsáry, 1889 (V. Soon pers. comm.).

**Plate 40.**
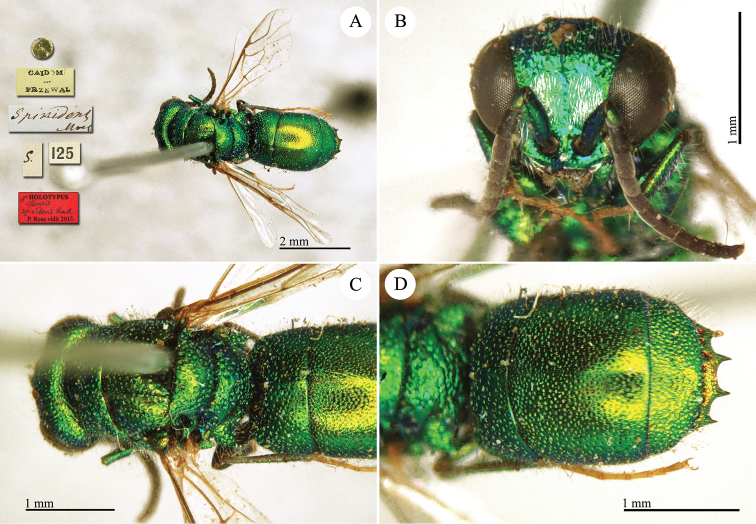
*Chrysis spinidens* Mocsáry, 1887, holotype. **A** Habitus, dorsal view **B** head, frontal view **C** mesosoma, dorsal view **D** metasoma, dorsal view.

##### Current status.

*Chrysis spinidens* Mocsáry, 1887.

#### Chrysis splendidula var. unica

Taxon classificationAnimaliaHymenopteraChrysididae

Radoszkowski, 1891

Chrysis splendidula var. unica Radoszkowski 1891a: 189.

##### Type locality.

“Ashabad”.

##### Syntype

1♂ [box 61]: Trans-Caspia // var. *unica* [handwritten by Radoszkowski].

##### Syntype

1♂ [box 61]: Trans-Caspia // var. *unica* ♂ [handwritten by Radoszkowski] // label with genitalia.

##### Syntype

1♀ [box 61]: Trans-Caspia // *unica* ♀ [handwritten by Radoszkowski].

##### Remarks.

As in other cases of species described in 1891 (e.g. *nova*, *simulatrix*), the specimens considered as syntypes bear the label “Trans-Caspia” and not “Ashabad”. Another female syntype is housed in HNHM bearing the labels: Trans-Kaspia / splendidula var. unica <handwritten by Radoszkovski> / Chrysis splendidula v. unica Rad. det. Mocsáry / id nr. 115606 HNHM Hym. coll. These syntypes are closely related to *Chrysis chlorisans* du Buysson (in André) by the colouration and sculpture of the body. It belongs to the *Chrysis splendidula* group.

##### Current status.

*Chrysis splendidula* Rossi, 1790 (synonymised by Kimsey and Bohart 1991: 465).

#### Chrysis subaurata

Taxon classificationAnimaliaHymenopteraChrysididae

Radoszkowski, 1891

Figure 7

Chrysis subaurata Radoszkowski 1891a: 192.

##### Type locality.

“Ashabad”.

##### Holotype

♂ [box 61]: golden rounded label // Asmabad [printed, sic] [yellow label] //*subaurata* [handwritten by Radoszkowski].

##### Remarks.

*Chrysis subaurata* is the green form of *Chrysis splendidula* Rossi, 1790. It was considered as a variation of *Chrysis splendidula* by du Buysson (in André) (1895: 534), Bischoff (1913: 60), Trautmann (1927: 170) and Linsenmaier (1951: 106). Kimsey and Bohart (1991) listed it as a valid species without type examination. It belongs to the *Chrysis splendidula* group.

**Figure 7.**
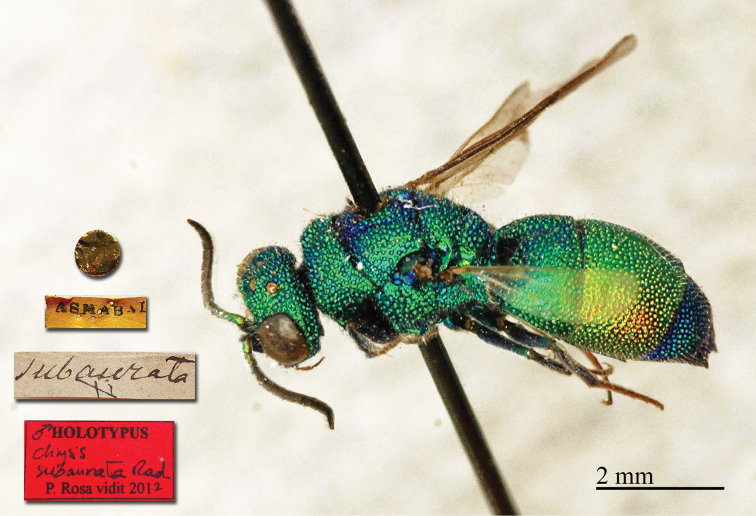
*Chrysis subaurata* Radoszkowski, 1891, holotype, habitus, dorso-lateral view.

##### Current status.

*Chrysis splendidula* Rossi, 1790 (synonymised by Linsenmaier 1951: 106).

#### Chrysis subcoerulea

Taxon classificationAnimaliaHymenopteraChrysididae

Radoszkowski, 1891

Chrysis subcoerulea Radoszkowski 1891a: 191.

##### Type locality.

“Ashabad”.

##### Possible Syntypes

11♂♂♀♀ [box 61]: Tr-Cap Saraks.

##### Remarks.

Under the name *Chrysis subcoerulea* R. there are eleven specimens collected at Saraks. One male was sent in loan [Mus-PAN Krakow] but it is the only specimen different from the others and not belonging to the current interpretation of the species. One female is labelled as *Chrysis subcoerulea* by Radoszkowski and it bears a golden rounded label indicating a type specimen in the Radoszkowski collection. It is not easy to state whether they are truly syntypes, since the collecting locality is different: Saraks instead of Ashabad. As in other cases related to the same publication, it is possible that Ashabad is a locality in error: the two localities are close to each other and the great part of the specimens collected in Turkmenistan come from these two localities. In many other cases the localities given by Radoszkowski in 1891 did not match the locality labels found under the specimens. Another similar specimen is found in MNHN. *Chrysis subcoerulea* is related with *Chrysis viridissima* Klug, 1845 and belongs to the same species group. Kimsey and Bohart (1991: 467) placed *Chrysis subcoerulea* in the *Chrysis comparata* *s. str.* group, without type examination.

After the examination of the male type of *Chrysis chlorochrysa* Mocsáry we propose the new synonym: *Chrysis subcoerulea* Radoszkowski, 1891 = *Chrysis chlorochrysa* Mocsáry, 1889. Du Buysson (1895: 500) already considered *Chrysis subcoerula* as the female of *chlorochrysa*, but curiously without synonymizing it (*Obs. - Le female décrit par M. le général O. Radoszkowsky appartient à la* Chrysis chlorochrysa *Mocs., d’après le spécimen que l’auteur a eu l’amabilité de m’envoyer.*).

##### Current status.

*Chrysis chlorochrysa* Mocsáry, 1889.

#### Chrysis succincta var. sparsepunctata

Taxon classificationAnimaliaHymenopteraChrysididae

du Buysson, 1895

Plate 41

Chrysis succincta var. sparsepunctata du Buysson (in André) 1895: 422.

##### Type locality.

“Patrie: Province transcaspienne: Saraks (Radoszkowsky)”.

##### Holotype

♀ [box 60]: Tr-Cap Saraks [printed] [yellow label] // Chrysis succincta L. var. sparsepunctata Buyss. v. nov. [handwritten by du Buysson].

##### Remarks.

This species belongs to the *Chrysis succincta* group. It was placed in the synonymic list of *Chrysis succincta* Linnaeus, 1767 by Kimsey and Bohart (1991) because it was described as one of its variations. The type of *Chrysis frivaldszkyi* Mocsáry was checked. *Chrysis frivaldszkyi* and relative species and subspecies are discussed in Rosa (2005) and Rosa and Xu (2015).

**Plate 41.**
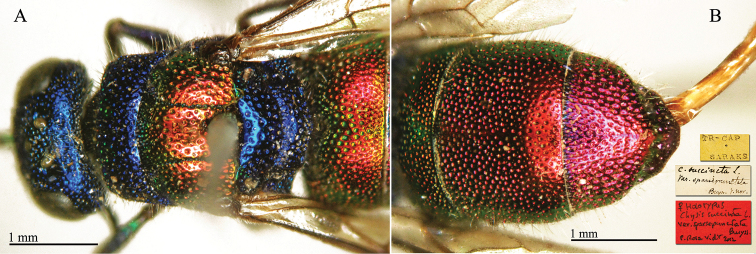
Chrysis succincta var. sparsepunctata du Buysson, 1892, holotype. **A** Head and mesosoma, dorsal view **B** metasoma, dorsal view.

##### Current status.

*Chrysis frivaldszkyi sparsepunctata* du Buysson (in André), 1895 (transferred by Linsenmaier 1959: 114).

#### Chrysis sznabli

Taxon classificationAnimaliaHymenopteraChrysididae

Radoszkowski, 1891

Chrysis Sznabli Radoszkowski 1891a: 196.

##### Type locality.

“Saraks”.

##### Holotype

♂ [box 62]: golden rounded label // Tr-Cap Saraks [printed] [yellow label] // *sznabli* [handwritten by Radoszkowski].

##### Remarks.

*Chrysis sznabli* belongs to the *Chrysis viridula* group.

##### Current status.

*Chrysis sznabli* Radoszkowski, 1891.

#### Chrysis taczanovskii

Taxon classificationAnimaliaHymenopteraChrysididae

Radoszkowsky, 1877

Plate 42

Chrysis Taczanovskii Radoszkowsky 1877 (1876): 146.

##### Type locality.

“Egypte et Abyssinie” [written in the introduction].

##### Holotype

♀ [box 62]: golden rounded label // Egyptus. [printed] [blue label] // Taczano [printed] // *Taczanovsk* [handwritten by Radoszkowski] // 220 [printed] // *taczanowski* [handwritten by Radoszkowski].

##### Remarks.

The type is seriously damaged, without forewings and right hind wing; it has no left hind-leg and tarsi of the left mid-leg and right hind-legs; it lacks the sternites and the internal segments. The African form has shorter F-I and narrower scapal basin compared with specimens from Middle East and Caucasian countries. It belongs to the *Chrysis taczanovskii* group.

**Plate 42.**
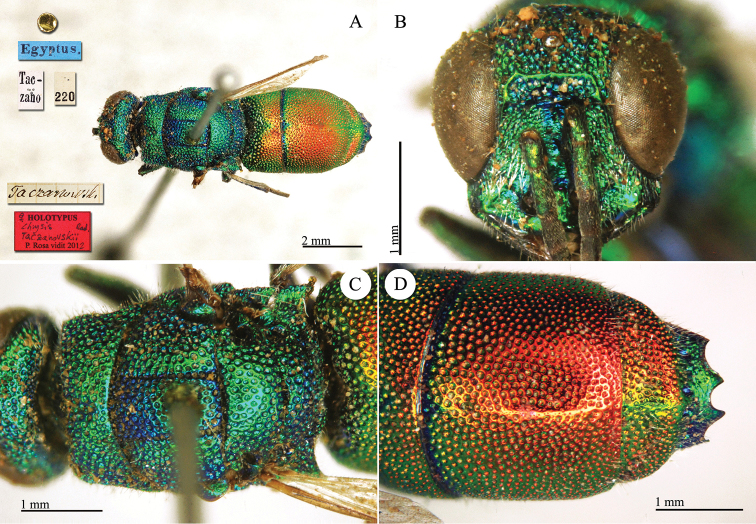
*Chrysis taczanovskii* Radoszkowsky, 1877, holotype. **A** Habitus, dorsal view **B** head, frontal view **C** mesosoma, dorsal view **D** metasoma, dorsal view.

##### Current status.

*Chrysis taczanovskii* Radoszkowsky, 1877.

#### Chrysis tasmanica

Taxon classificationAnimaliaHymenopteraChrysididae

Mocsáry, 1889

Chrysis (Hexachrysis) tasmanica Mocsáry 1889: 563.

##### Type locality.

“Tasmania (Coll. Rad.)”.

##### Holotype

♂ [box 62]: golden rounded label // 137 [printed] // Tasman. [printed] // *tasmanica* Mocs [handwritten by Radoszkowski].

##### Remarks.

*Chrysis tasmanica* belongs to the *Chrysis smaragdula* group.

##### Current status.

*Chrysis tasmanica* Mocsáry, 1889.

#### Chrysis taurica

Taxon classificationAnimaliaHymenopteraChrysididae

Mocsáry, 1889

Plate 43

Chrysis (Tetrachrysis) taurica Mocsáry 1889: 345.

##### Type locality.

“Patria: Tauria [Krim] (Coll. Rad.)”.

##### Holotype

♀ [box 62]: golden rounded label // Tauria [printed] // *taurica* [handwritten by Radoszkowski] // 131 [printed].

##### Remarks.

*Chrysis taurica* belongs to the *Chrysis varidens-ragusae* group.

**Plate 43.**
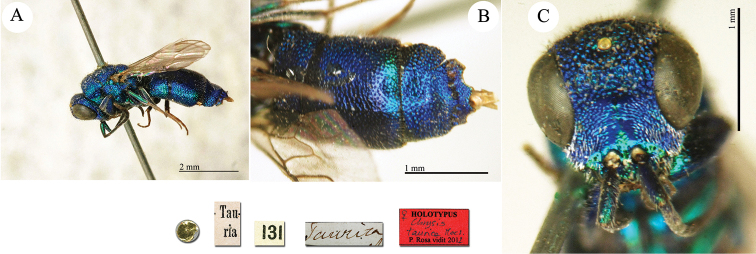
*Chrysis taurica* Mocsáry, 1889, holotype. **A** Habitus, lateral view **B** metasoma, dorso-lateral view **C** head, frontal view.

##### Current status.

*Chrysis ragusae* De Stefani, 1888 (synonymised by Trautmann 1927: 143).

#### Chrysis tenella

Taxon classificationAnimaliaHymenopteraChrysididae

Mocsáry, 1889

Plate 44

Chrysis (Olochrysis) tenella Mocsáry 1889: 197.

##### Type locality.

“Caucasus (Coll. Rad.)”.

##### Holotype

♂ [not ♀] [box 60]: Caucasus [printed] // *unicolor* ? [handwritten by Radoszkowski] // 273 [handwritten by Mocsáry] // *tenella* Moc [handwritten by Radoszkowski].

##### Remarks.

The type lacks two segments of the left antenna, right wings, tibia and tarsi of the left fore-leg as well as tarsi of the right hind-leg. It is closely related to *Chrysis chalcophana* Mocsáry; the main difference is found only in the third tergite, particularly in the shape of the pit-row. It belongs to the *Chrysis millenaris* group.

**Plate 44.**
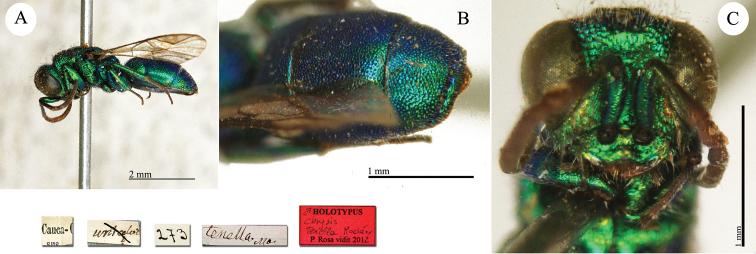
*Chrysis tenella* Mocsáry, 1889, holotype. **A** Habitus, lateral view **B** metasoma, dorso-lateral view **C** head, frontal view.

##### Current status.

*Chrysis tenella* Mocsáry, 1889.

#### Chrysis therates

Taxon classificationAnimaliaHymenopteraChrysididae

Mocsáry, 1889

Plate 45

Chrysis (Hexachrysis) therates Mocsáry 1889: 555.

##### Type locality.

“Senegalia (Coll. Rad.)”.

##### Holotype

♀ [box 62]: golden rounded label // Senegal. [printed] [green label] // 134 [printed] // *modica* [handwritten by Radoszkowski] // *therates* Moc [handwritten by Radoszkowski].

##### Remarks.

Kimsey and Bohart (1991: 438) synonymised *Chrysis therates* with *Chrysis mediocris* Dahlbom, 1845 without type examination. *Chrysis therates* is cleary separated from *Chrysis mediocris* even though it belongs to the *Chrysis smaragdula* group. The type perfectly matches Mocsáry’s description, but this specimen seems to be collected in another biogeographical region. Shape and colour pattern are typical of the Oriental Region. We identify this species as *Chrysis principalis* Smith. We did not examined the type of *Chrysis principalis* yet, however this specimen matches all the specimens of *Chrysis principalis* observed in different collections, including those in Linsenmaier’s collection, who examined Smith types (pers. comm. based on unpublished manuscripts found in NML). Very likely, Mocsáry described *Chrysis therates* as a new species because bearing the label “Senegal”, and no other African species shares similar characteristics. The locality label of *Chrysis therates* could be in error or this specimen could be accidentally introduced into Senegal by commerce. In fact, Senegal was on the commercial way from South Asia to Europe, and the specimen could be present on any ship along this route. Therefore, we propose the new synonym: *Chrysis therates* Mocsáry, 1889 = *Chrysis principalis* Smith, 1874.

**Plate 45.**
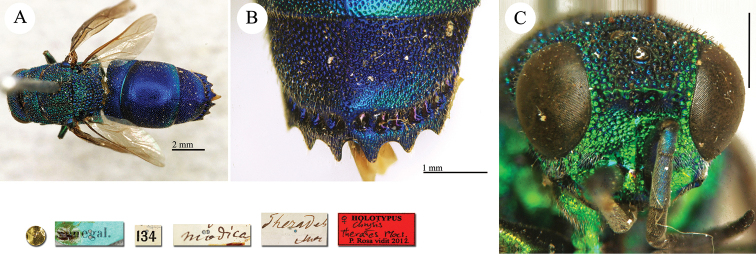
*Chrysis therates* Mocsáry, 1889, holotype. **A** Habitus, lateral view **B** third metasomal tergite, dorsal view **C** head, frontal view.

##### Current status.

*Chrysis principalis* Smith, 1874.

#### Chrysis tolteca

Taxon classificationAnimaliaHymenopteraChrysididae

Mocsáry, 1889

Chrysis (Tetrachrysis) tolteca Mocsáry 1889: 341.

##### Type locality.

“Patria: Mexico (Coll. Rad.)”.

##### Holotype

♀ [box 62]: golden rounded label // Mexico. [printed] [green label] // *Toldeca* [handwritten by Radoszkowski] // 138 [printed].

##### Current status.

*Exochrysis tolteca* (Mocsáry, 1889) (transferred by Kimsey and Bohart 1991: 503).

#### Chrysis transcaspica

Taxon classificationAnimaliaHymenopteraChrysididae

Mocsáry, 1889

Plate 46

Chrysis (Gonochrysis) transcaspica Mocsáry 1889: 306.

##### Type locality.

“Patria: Territorium transcaspicum (Coll. Rad.)”.

##### Holotype

♀ [box 60]: Trans-Caspia [printed] [yellow label] // *transcaspica* Moc [handwritten by Radoszkowski] // 274 [handwritten by Mocsáry].

##### Remarks.

In Kimsey and Bohart (1991: 407), *Chrysis transcaspica* was placed in synonymy with *Chrysis elegans* Brullé, 1833. Rosa et al. (2013: 32) revalidated the species based on the different shape of the anal teeth, colour and punctuation. It belongs to the *Chrysis elegans* group.

**Plate 46.**
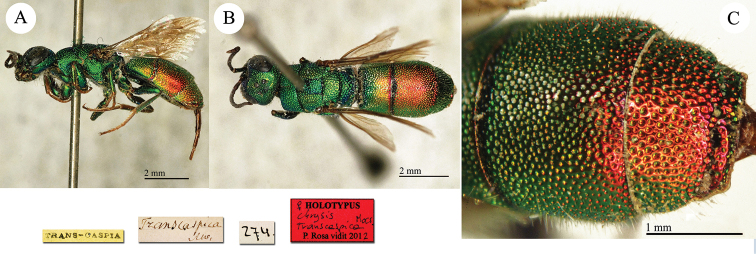
*Chrysis transcaspica* Mocsáry, 1889, holotype. **A** Habitus, lateral view **B** habitus, dorsal view **C** metasoma, second and third metasomal tergites, dorsal view.

##### Current status.

*Chrysis transcaspica* Mocsáry, 1889.

#### Chrysis transcaspica var. nostra

Taxon classificationAnimaliaHymenopteraChrysididae

Radoszkowski, 1891

Plate 47

Chrysis transcaspica var. nostra Radoszkowski 1891a: 184.

##### Type locality.

“Gedzen”.

##### Holotype

♀ [box 60]: Gedzen [handwritten] [yellow label] // var *nostra* [handwritten by Radoszkowski] // transcaspica var nostra [handwritten by Radoszkowski].

##### Remarks.

Chrysis transcaspica var. nostra was described by Radoszkowski (1891) mainly based on colours: “*Premier article des antennes cuivré; premier segment abdominal feu-doré, deuxième et troisième d’un rouge carminé.*”. Kimsey and Bohart (1991: 407) synonymised *Chrysis transcaspica* Mocsáry, 1889 and Chrysis transcaspica var. nostra with *Chrysis elegans* Lepeletier, 1806. It belongs to the *Chrysis elegans* group.

**Plate 47.**
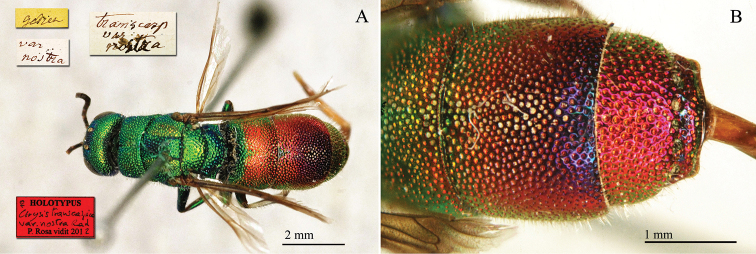
Chrysis transcaspica var. nostra Radoszkowski, 1891, holotype **A** habitus, dorsal view **B** metasoma, dorsal view.

##### Current status.

*Chrysis transcaspica* Mocsáry, 1889.

#### Chrysis trisinuata

Taxon classificationAnimaliaHymenopteraChrysididae

Mocsáry, 1889

Plate 48

Chrysis (Gonochrysis) trisinuata Mocsáry 1889: 288.

##### Type locality.

“Patria: Turkestania (Taschkend, Coll. Rad.)”.

##### Holotype

♀ [box 60]: golden rounded label // Tachkend [printed] // 117 [printed] // [unreadeable] [handwritten] // *trisinuata* Moc. [handwritten by Radoszkowski].

##### Remarks.

The taxonomic position of this species is not clear. The very short malar space (less than 1 MOD); feeble transverse frontal carina joined to the upper margin of the scapal basin; micropunctuated scapal basin; prolonged and teethless anal margin suggest that *Chrysis trisinuata* could belong to the genus *Chrysidea* Bischoff. However the general habitus, large dimensions (about 7 mm) and the complete closed cells on the wings place it in the genus *Chrysis* Linnaeus.

**Plate 48.**
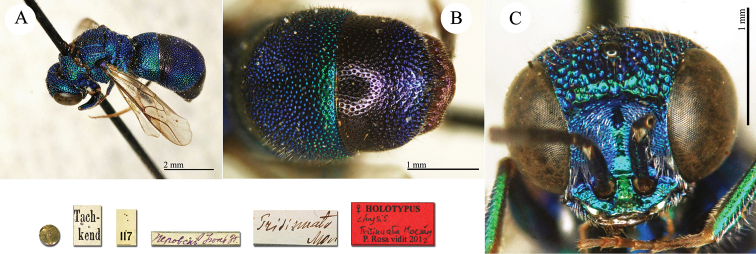
*Chrysis trisinuata* Mocsáry, 1889, holotype. **A** Habitus, dorso-lateral view **B** second and third metasomal tergites, dorsal view **C** head, frontal view.

##### Current status.

*Chrysis trisinuata* Mocsáry, 1889.

#### Chrysis uljanini

Taxon classificationAnimaliaHymenopteraChrysididae

Radoszkowski, 1877

Plate 49

Chrysis Uljanini Radoszkowski 1877: 22.

##### Type locality.

“Habitat in valle Sarafschan et in desertis prope Taschkent”, “Видъ этотъ пойманъ 19 и 27 мая 1869 г. въ Заравшанской долинѣ, 19 мая 1871 г. въ степи между Сыръ-дарьей и Ташкентомъ” [This species was collected on the 19^th^ and 27^th^ of May 1869 in the Zaravshan Valley and the 19^th^ May 1871 in steppe between Syr-Darya and Tashkent].

##### Paralectotype

1♂ [box 62]: golden rounded label // Ϲтепь м. Ϲ. д. и Т. [printed] // 19. [printed] [pink label with red line].

##### Paralectotype

1♀ [box 61]: golden rounded label // Верхн. Заравш. // *ulianini* [handwritten by Radoszkowski] // 27. [printed] [pink label] // 52 [printed] // *sarafschana* Mocs. [handwritten by Mocsáry].

##### Remarks.

Bohart (in Kimsey and Bohart 1991: 473) designated the lectotype at MMU. In Radoszkowski’s collection, under the name *Uljanini* R. there are three male specimens belonging to the *Chrysis cerastes* group, with very short F-I and F-II: one male paralectotype of *Chrysis uljanini*, and other two specimens with the same particular colour and thoracic punctuation. The first specimen is the syntype (currently paralectotype) listed by Radoszkowski as: Ϲтепь м. Ϲ. д. и Т. [printed] // 19. [= 19 мая 1871 г. въ степи между Ϲыръ-Дарьей и Ташкентомъ” in the Russian description]. The other two specimens were collected at: “Iskender [Iskenderund?] 20.jul.1870” and “Kizilkum 30 Aug 1870” and they cannot be considered as paralectotypes of *Chrysis uljanini*. The specimen collected at Kizilkum was dissected by Radoszkowski, who drew the genitalia in his revision (1889: tab.IV, fig. 51). For further remarks see under *Chrysis uljanini* Radoszkowski & Mocsáry and *Chrysis sarafschana* Mocsáry. The second paralectotype is the female selected by Mocsáry as the holotype of *Chrysis sarafschana* Mocsáry, 1889. It belongs to the *Chrysis ignita* group.

**Plate 49.**
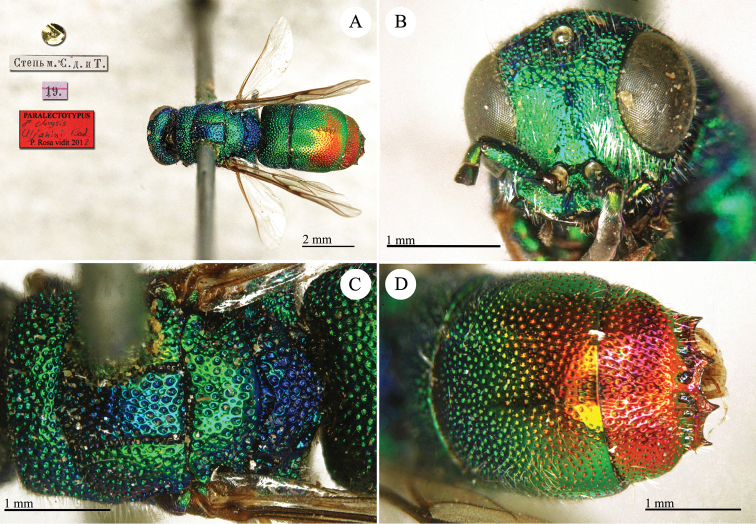
*Chrysis uljanini* Radoszkowski, 1877, paralectotype. **A** Habitus, dorsal view **B** head, frontal view **C** mesosoma, dorsal view **D** second and third metasomal tergites, dorsal view.

##### Current status.

*Chrysis uljanini* Radoszkowski, 1877.

#### Chrysis uljanini

Taxon classificationAnimaliaHymenopteraChrysididae

Radoszkowski & Mocsáry, 1889

Chrysis Uljanini Radoszkowski and Mocsáry 1889: 436 *nec* Radoszkowski, 1877.

##### Type locality.

“Turkestania (Kisil-kum. Coll. Rad.)”.

##### Holotype

♂ [box 62]: Kizilkum 30 Aug 1870 / label with genitalia / *Uljanini* Rad. Mocs / *Chrysis kizilkumiana* Rosa det. P. Rosa 2012

##### Remarks.

Mocsáry (1889) studied two specimens of *Chrysis uljanini* lent by Radoszkowski. The female was the syntype collected at Zaravshan on the 27^th^ May 1896, while the male was erroneously considered as syntype. In fact, the male specimen was collected at Kizilkum on the 30^th^ of August 1870 and was not listed in the original description by Radoszkowki (1877), therefore it cannot be considered as a syntype. Radoszkowki (1889: fig. 51) drew the genitalia of this specimen in the revision of the genial capsulae of the Chrysididae. The male belongs to a different species and is conspecific with the male paralectotype of *Chrysis uljanini* Radoszkowki collected at Tashkent and housed in ISEA-PAN.

Mocsáry (1889) understood that the two specimens of *Chrysis uljanini* belong to two different species: *Clariss.*[imo] *Auctor* [Radoszkowski] *sub nomine* Chrysis Uljanini, *secundum specimina typica, duas descripserat species bene distinctas et ego denominationem solum ad marem, etiam depictum, restringo et feminam distinguendae esse censeo* [based on the type specimens, Radoszkowski described two well distinct species under the name *Chrysis Uljanini* and I limit this name only to the male, also depicted, and the female has to be separate].

Therefore Mocsáry (1889) considered the male from Kizilkum as *Chrysis uljanini*, but redescribed it under the name *Chrysis uljanini* Radoszkowski & Mocsáry. He also described the female syntype of *Chrysis uljanini* as *Chrysis sarafashana* Mocsáry (1889).

The following authors followed Mocsáry’s (1889) interpretation and considered *Chrysis uljanini* in the *Chrysis cerastes* group (*sensu* Linsenmaier 1959) and *Chrysis sarafashana* in the *Chrysis ignita* group (Radoszkowki 1891: 190): Dalla Torre (1892: 92, 104), du Buysson (in André) (1895: 506, 512), Bischoff (1913: 58, 61), Tsuneki (1953: 27), Linsenmaier (1959: 159). The same identifications can be found in the most important European collections of Linsenmaier (NMLS), Zimmermann (NHMW) and Semenov-Tian-Shanskij (ZIN). Only Nikol’skaja (in Semenov-Tian-Shanskij and Nikol’skaya 1954: 130) gave the name *Chrysis uljanini* to specimens belonging to the *Chrysis ignita* group.

At the beginning of 1990 the situation was clear: there were two species (*Chrysis uljanini* and *Chrysis sarafashana*) belonging to two different species groups (*Chrysis cerastes* and *Chrysis ignita* groups), but the lectotype of *Chrysis uljanini* should be still designated, based on the male syntype, housed in Krakow and collected at Tashkent, to fix the current interpretation of the two species.

Bohart (in Kimsey and Bohart 1991) designated one female syntype found in MMU and belonging to the *Chrysis ignita* group as the lectotype of *Chrysis uljanini*. Thus, the name *Chrysis sarafashana* fallen in synonymy with *Chrysis uljanini* and the males belonging to the *Chrysis cerastes* group, till then known as *Chrysis uljanini*, remained without any name.

To clarify the situation, we consider the name *Chrysis uljanini* Radoszkowski & Mocsáry, 1889 as a junior primary homonym of *Chrysis uljanini* Radoszkwski, 1877. In fact, Mocsáry did not study nor redescribe the syntype male of *Chrysis uljanini*, but a different specimen collected at Kizilkum. The evidence of the description of a new species can be found in Mocsáry’s text (1889: 436) and in the index (1889: 633): Mocsáry considered this taxon as *Chrysis uljanini* Radoszkowki & Mocsáry, 1889 and not as *Chrysis uljanini* Radoszkowki, 1877. This is the only case in which Mocsáry added his name after the original author name. Therefore all the citations of *Chrysis uljanini* published from 1889 until Kimsey and Bohart’s monograph (1991) (excluding Semenov-Tian-Shanskij and Nikol’skaja 1954) should be referred to this taxon.

Since *Chrysis uljanini* Radoszkowski & Mocsáry, 1889 has to be considered as a primary homonym of *Chrysis uljanini* Radoszkowki, 1877, we replace it with *Chrysis kizilkumiana* Rosa, **new name.** The etymology of this name refers to the collecting place. The holotype of this species is the male studied by Mocsáry and bearing the following labels: “Kizilkum 30 Aug 1870” and “*Uljanini* Rad. Mocs” [handwritten by Mocsáry]. The accurate description of this taxon is given by Mocsáry (1889: 436) and the drawing of the genital capsule is given by Radoszkowki (1889: tab. IV, fig. 51). The type is conspecific with the paralectotype male of *Chrysis uljanini* Radoszkowski, whose figures can be found in this article (Plate 49). It belongs to the *Chrysis cerastes* group.

##### Current status.

*Chrysis kizilkumiana* Rosa, replacement name for *Chrysis uljanini* Radoszkowski & Mocsáry, 1889 *nec* Radoszkowski, 1877.

#### Chrysis vagans

Taxon classificationAnimaliaHymenopteraChrysididae

Radoszkowski, 1877

Plate 50

Chrysis vagans Radoszkowski 1877: 11.

##### Type locality.

“Habitat in valle Sarafschan et in monte Karak”, “Пойманъ 13 мая 1869 г. въ Джамскомъ ущельи и 6 мая 1871 г. на горѣ Каракъ” [collected on the 13^th^ of May 1869 in the Canyon Djamsk, and on the 6^th^ of May 1871 on the Karak mountain].

##### Lectotype

(here designated) 1♂ [not ♀] [box 60]: golden rounded label // Каракъ [printed] // *vagans* [handwritten by Radoszkowski] // Spinthr. [handwritten by Radoszkowski] // 6 [pink label with red line] // 46 [printed].

##### Remarks.

One male paralectotype is housed in MMU (Kimsey and Bohart 1991: 558). *Chrysis vagans* is the type species of the genus *Spintharina* Semenow. We here designate the lectotype on the specimen housed in ISEA-PAN because the specimen in the Fedtschenko collection in MMU does not belong to the same species. The latter belongs to a similar species with different face in frontal view, without distinct TFC and characteristic antero-basal lobe on the third tergite. It belongs to the *Spintharina vagans* group.

**Plate 50.**
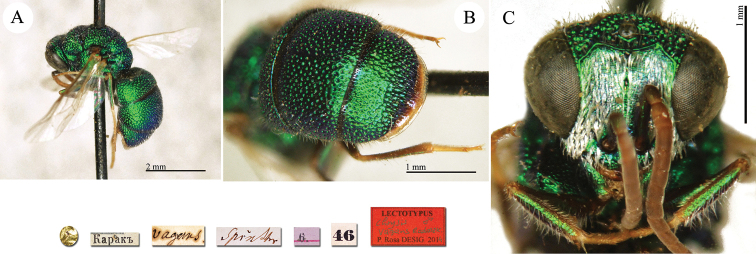
*Chrysis vagans* Radoszkowski, 1877, lectotype. **A** Habitus, dorso-lateral view **B** second and third metasomal tergites, dorsal view **C** head, frontal view.

##### Current status.

*Spintharina vagans* (Radoszkowski, 1877) (transferred by Bohart 1987: 93).

#### Chrysis viridans

Taxon classificationAnimaliaHymenopteraChrysididae

Radoszkowski, 1891

Plate 51

Chrysis viridans Radoszkowski 1891a: 192.

##### Type locality.

“Ashabad”.

##### Holotype

♀ [box 62]: golden rounded label // Ashabad [printed] [yellow label] // *viridicans* [sic! handwritten by Radoszkowski].

##### Remarks.

Kimsey and Bohart (1991: 396) synonymised *Chrysis viridans* with *Chrysis chrysochlora* Mocsáry, 1889. In this paper we place *Chrysis chrysochlora* in synonymy with *Chrysis keriensis*. Therefore, *Chrysis viridans* can be considered as a synonym of *Chrysis keriensis*. Another specimen identified by Radoszkowski is housed in MNHN (general collection box 41). It belongs to the *Chrysis ignita* group.

**Plate 51.**
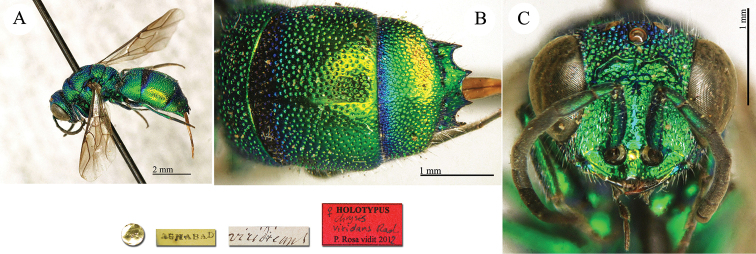
*Chrysis viridans* Radoszkowski, 1891, holotype. **A** Habitus, dorso-lateral view **B** second and third metasomal tergites, dorsal view **C** head, frontal view.

##### Current status.

*Chrysis keriensis* Radoszkowski, 1887.

#### Cleptes morawitzi

Taxon classificationAnimaliaHymenopteraChrysididae

Radoszkowski, 1877

Plate 52

Cleptes Morawitzi Radoszkowski 1877: 1.

##### Type locality.

“Habitat prope Maracandam, Taschkent et Tschardara”, “Пойманъ 5, 12, 13 и 19 апрѣля 1869 г. въ Самаркандѣ; 3, 5, 8 и 25 апрѣля 1871 г. въ Ташкентѣ и Чардарѣ” [collected on the 5^th^, 12^th^, 13^th^ and 19^th^ of April 1869 at Samarkand; on the 3^rd^, 5^th^, 8^th^ and 25^th^ of April 1871 at Tashkent and Tschardara].

##### Paralectotype

1♀ [box 59]: 3 [green label with red line] // Taschkent [printed in cyrillic].

##### Paralectotype

1♀ [box 59]: 5 [green label with red line] // Taschkent [printed in cyrillic].

##### Paralectotype

1♂ [box 59]: 8 [green label with red line] // label with genitalia // Taschkent [printed in cyrillic].

##### Remarks.

Móczár (1997: 39) designated the lectotype of *Cleptes morawitzi* in MNHU. Three paralectotypes are housed in Kraków. The male bears a label with only part of the dissected genitalia. Radoszkowski (1889: 6, tab. I) delineated it in his revision on the genital capsules of the Chrysididae (fig. 4a, 4b, 4c). In the description, Radoszkowski did not mention the number of specimens examined, but in the type series there were males and females, and the number of specimens examined was not less than eight (comparing the dates of collection), thus we consider the specimens in the Kraków collection as paralectotypes. They match the original description and the lectotype in MNHU. Five paralectotypes are also housed in Fedtschenko’s collection in MMU, one paralectotype is deposited in MSNG (Rosa 2009).

**Plate 52.**
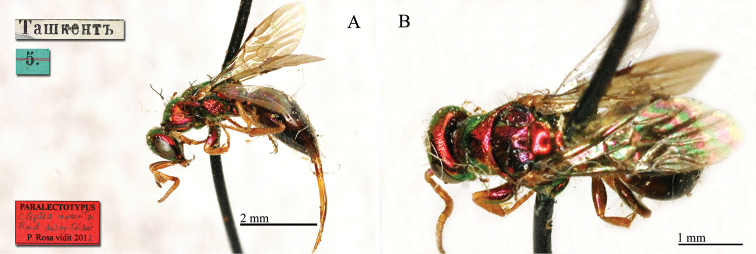
*Cleptes morawitzi* Radoszkowski, 1877, paralectotype. **A** Habitus, lateral view **B** habitus, dorsal view.

##### Current status.

*Cleptes morawitzi* Radoszkowski, 1877.

#### Cleptes radoszkowskii

Taxon classificationAnimaliaHymenopteraChrysididae

Mocsáry, 1889

Plate 53

Cleptes Radoszkowskii Mocsáry (Inédite) (in Radoszkowski) 1889: 7.

##### Type locality.

“Caucase (Mlokosewitz)”.

##### Lectotype

♀ [box 59]: Caucas Nlokos [printed, sic!] // Lectotypus [printed] Cleptes ♀ radoszkowskii Rad. des. Móczár 997 [handwritten] [red label].

##### Paralectotype

1♀ [box 59]: Caucas Mlok [printed] // golden rounded label // *Radoszkovsk* Moc [handwritten] // 104 [printed] // Paralectotypus [printed] Cleptes ♀ radoszkowskii Rad. des. Móczár 1997 [handwritten] [red label].

##### Paralectotype

1♂ [box 59]: a-Cauc [printed] // Paralectotypus *Cleptes radoszkowskii* Rad. des. Móczár 1996 [handwritten] [red label] // *Cleptes femoralis* [handwritten] det. L. Móczár, 1996.

##### Remarks.

Kimsey and Bohart (1991: 63) included *Cleptes radoszkowskii* in the *Cleptes semiauratus* group. Móczár (1997: 32) placed *Cleptes radoszkowskii* in the subgenus *Holcocleptes*, *Cleptes aerosus* group, after type examination. Móczár (1998: 338) added detailed informations, keys, description of the male and the lectotype designation. He found that the male paralectotype of *Cleptes radoszkowskii* belongs to a different species: *Cleptes femoralis* Mocsáry, 1889.

**Plate 53.**
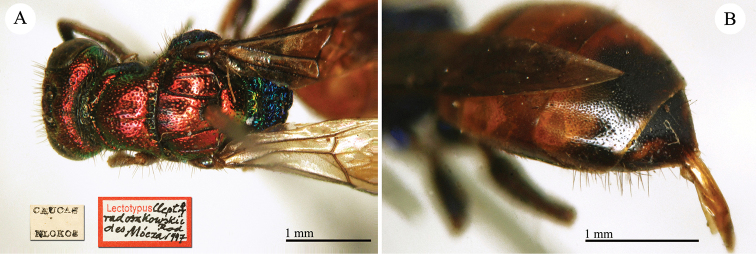
*Cleptes radoszkowskii* Mocsáry, 1889, lectotype. **A** Head and mesosoma, dorsal view **B** metasoma, dorso-lateral view.

##### Current status.

*Cleptes radoszkowskii* Mocsáry, 1889.

#### Elampus ambiguus

Taxon classificationAnimaliaHymenopteraChrysididae

Eversmann, 1857

Plate 54

Elampus ambiguus Eversmann 1857: 549 *nec* Dahlbom, 1854.

##### Type locality.

“Cepi in provincia Saratoviensi”.

##### Holotype

♂ [box 59]: scutellum armatum [hadwritten by Eversmann] // red rounded label // blue rounded label // *Elampus ambiguus* Dlbm [handwritten by Eversmann] // Sarat. [handwritten] // golden rounded label // *Evermanni* Mocs. [handwritten by Radoszkowski] // 105 [printed].

##### Remarks.

The description provided by Linsenmaier (1959: 23) is mostly accurate. The main difference is in the colour of the specimen. The type is not entirely black-violet, but rather blue, with a few light blue metallic reflections on legs and on the lateral sides of mesonotum. Only the metanotum and the metanotal projection appear dark blue to dull black.

**Plate 54.**
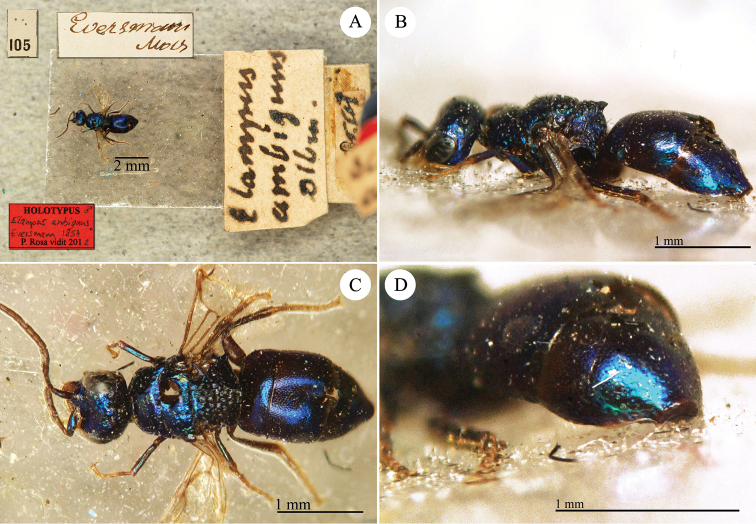
*Elampus ambiguus* Eversmann, 1857, holotype. **A** Habitus, dorsal view **B** habitus, lateral view **C** habitus, dorsal view **D** third metasomal tergite, posterior view.

##### Current status.

*Elampus eversmanni* (Mocsáry, 1889), replacement name for *Elampus ambiguus* Eversmann, 1857.

#### Elampus bidentatus

Taxon classificationAnimaliaHymenopteraChrysididae

Eversmann, 1857

Elampus bidentatus Eversmann 1857: 548.

##### Type locality.

“Cepi in promont. Uralensib.”.

##### Holotype

♀ [box 59]: Spa... Juni? [handwriting only partly readable] // *Elampus bidentulus* Kl. Dlbm. [handwritten by Eversmann] // brawn rounded label // red rounded label.

##### Remarks.

The examination of the type confirms that the name *Elampus bidentatus* is merely an incorrect spelling of *Elampus bidentulus* (Lepeletier, 1806). Eversmann listed this species as “*Elampus bidentatus* Klug, Dalbm”. But the identification label attached to the type specimen reads “*Elampus bidentulus* Kl. Dlbm” in Eversmann’s handwriting. Dahlbom (1854) listed *Elampus bidentulus* Klug [and not *bidentulus* Lepeletier] in the dichotomous key (pagg.: 38, 39), in the index (pag. 406), and in the list of examined specimens (pag. 40). Eversmann was confused by this mistake. Dahlbom did not examine Lepeletier’s type and based his keys and description on a single specimen labelled by Klug as *Elampus bidentulus* in MNHU. The specimen matches the current interpretation of *Philoctetes bidentulus* (Lepeletier) (Rosa 2006a; Rosa and Xu 2015) and the name can simply be considered an incorrect subsequent spelling.

##### Current status.

*Philoctetes bidentulus* (Lepeletier, 1806) (synonymised by Mocsary 1889: 85; transferred by Niehuis 2000).

#### Elampus femoralis

Taxon classificationAnimaliaHymenopteraChrysididae

Eversmann, 1857

Elampus femoralis Eversmann 1857: 547.

##### Type locality.

“Cepi in prov. Casanensi”.

##### Holotype

♀ [box 59]: golden rounded label // red rounded label // *Elampus femoralis* Evm [handwritten by Eversmann] // Kas. [handwritten by Radoszkowski] // type D. Evers. [handwritten] // 80 [printed].

##### Remarks.

The specimen was originally pinned, and later glued on a plastic label. The right fore-leg is glued apart; it lacks some tarsi in all the legs, with the exception of the right fore-leg and the left hind-leg.

##### Current status.

*Elampus bidens* (Förster, 1853) (synonymised by Mocsáry 1889: 73).

#### Elampus mocsaryi

Taxon classificationAnimaliaHymenopteraChrysididae

Radoszkowski, 1887

Plate 55

Elampus Mocsari (!) Radoszkowski 1887: 45.

##### Type locality.

“Zaïdam”.

##### Holotype

♀ [box 59]: Gaidam Przewal [printed] [yellow label] // *Mocsaryi* Rad [handwritten by Mocsáry] // golden rectangular label // 193 [printed].

##### Remarks.

The holotype is badly damaged. It lacks the right flagellum and pedicellus; nine flagellomeres of the left antenna; both fore-legs and the right hind-leg; the metasoma is glued to the locality label.

**Plate 55.**
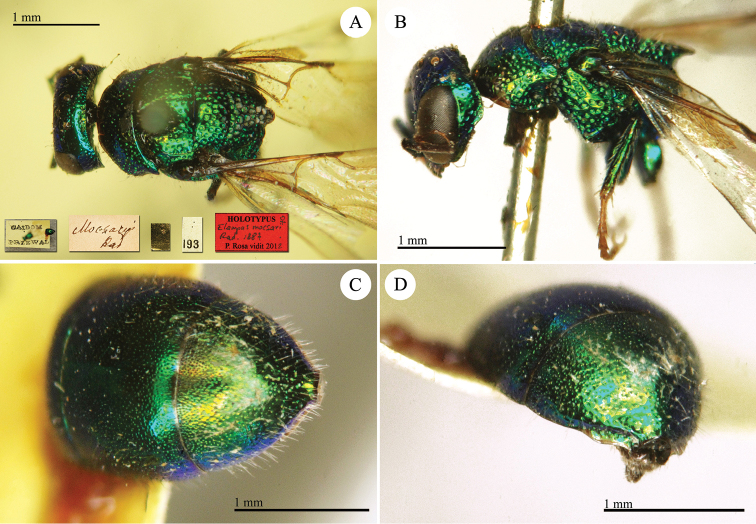
*Elampus mocsaryi* Radoszkowski, 1887, holotype. **A** Head and mesosoma, dorsal view **B** head and mesosoma, lateral view **C** metasoma, dorsal view **D** third metasomal tergite, posterior view.

##### Current status.

*Elampus mocsaryi* Radoszkowski, 1887 (emendated by Mocsáry 1889: 80).

#### Ellampus araraticus

Taxon classificationAnimaliaHymenopteraChrysididae

Radoszkowski, 1890

Plate 56

Ellampus araraticus Radoszkowski 1890 (1889): 508.

##### Type locality.

“Ararat, entre Sardar-Abadu et Sarabandy (13,000’)” [given in the introduction].

##### Syntype

♀ [box 59]: golden rounded label // Ararat [printed] [yellow label] // *Ellampus araraticus* Rad. [handwritten by Mocsáry].

##### Remarks.

Kimsey and Bohart (1991: 224) placed *Ellampus araraticus* in the genus *Holophris*, without type examination. Based on the type analysis, *Ellampus araraticus* belongs to the genus *Philoctetes* *sensu* Kimsey and Bohart (1991). Another possible syntype is housed in MNHN (general collection box 5). The type is in perfect condition.

**Plate 56.**
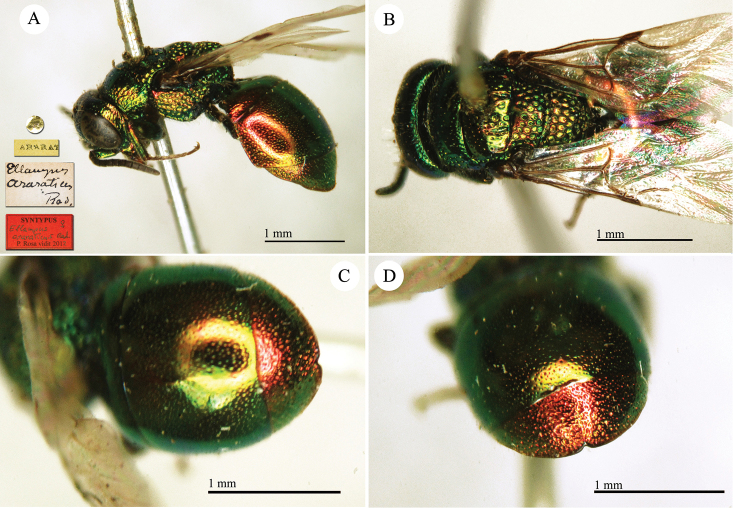
*Ellampus araraticus* Radoszkowski, 1890, syntype. **A** Habitus, lateral view **B** habitus, dorsal view **C** metasoma, dorso-lateral view **D** third metasomal tergite, posterior view.

##### Current status.

*Philoctetes araraticus* Radoszkowski, 1890, **comb. n.**

#### Ellampus hypocrita

Taxon classificationAnimaliaHymenopteraChrysididae

du Buysson, 1893

Plate 57

Ellampus hypocrita du Buysson 1893: 246.

##### Type locality.

“Mongolie: Kansu-Ielisyn-Kuse (Radoszkowsky); Perse: mer Caspienne occidentale”.

##### Paralectotype

1♀ [box 59]: golden rounded label // *Ellampus hypocrites* n.sp. *Buyss*. [handwritten by du Buysson] // Kansu Jelisyn Kuse 20/VII [handwritten].

##### Remarks.

The species was described on two specimens conserved in MNHN and Kraków. Kimsey and Bohart (1991) included *Ellampus hypocrita* in the genus *Omalus*, but the punctuation on the mesonotum and the shape of the mesopleuron are typical characteristics of the genus *Pseudomalus*, therefore we propose the new combination *Pseudomalus hypocrita* (du Buysson, 1893). This specimen is close to *Pseudomalus turkestanicus* (Mocsáry, 1889), but the margin of the third tergite has a distinct transparent rim, deeply incised in the middle, with two evident undulations similar to teeth at the notch base. Kimsey and Bohart (1991: 248) designated the lectotype by inference of "holotype" (ICZN art. 74.6).

**Plate 57.**
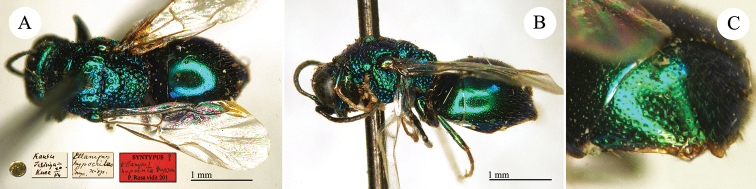
*Ellampus hypocrita* du Buysson, 1893, paralectotype. **A** Habitus, dorsal view **B** habitus, lateral view **C** third metasomal tergite, posterior view.

##### Current status.

*Pseudomalus hypocrita* (du Buysson, 1893), **comb. n.**

#### Ellampus montanus

Taxon classificationAnimaliaHymenopteraChrysididae

Mocsáry, 1890

Plate 58

Ellampus (Notozus) montanus Mocsáry 1890: 49.

##### Type locality.

“Patria: Montes Ararat Armeniae, ibidem a Cl. Dom. Mlokosewitz detectus (Coll. Radoszkovszkyi)”.

##### Holotype

♂ [box 59]: golden rounded label // Ararat [printed] [yellow label] // Ellampus n.sp. montanus Mocs [handwritten by Mocsáry].

##### Remarks.

The type lacks the right flagellum.

**Plate 58.**
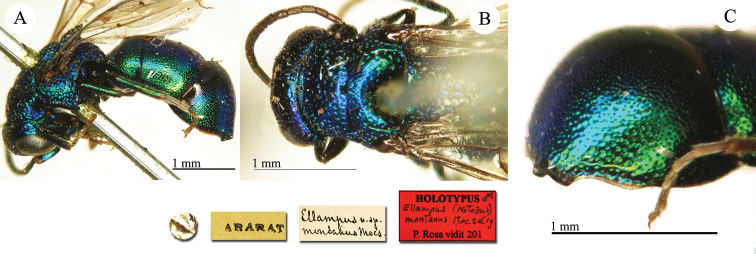
Ellampus (Notozus) montanus Mocsáry, 1890, holotype. **A** Habitus, lateral view **B** mesosoma, dorsal view **C** third metasomal tergite, dorso-lateral view.

##### Current status.

*Elampus montanus* (Mocsáry, 1890) (transferred by Kimsey and Bohart 1991: 168).

#### Ellampus obesus

Taxon classificationAnimaliaHymenopteraChrysididae

Mocsáry, 1890

Plate 59

Ellampus (Notozus) obesus Mocsáry 1890: 48.

##### Type locality.

“Patria: territorium Transcaspicum (Turcomania), a Dom. E. König detectus (Coll. Radoszkovszkyi)”.

##### Holotype

♂ [box 59]: Frans-Caspi G. Turcmenien E. König. [printed] // golden rounded label // Ellampus n.sp. obesus Mocs. [handwritten by Mocsáry].

##### Remarks.

Radoszkowski (1893) described the female of *Ellampus obesus* with the name *Ellampus komarowi*.

**Plate 59.**
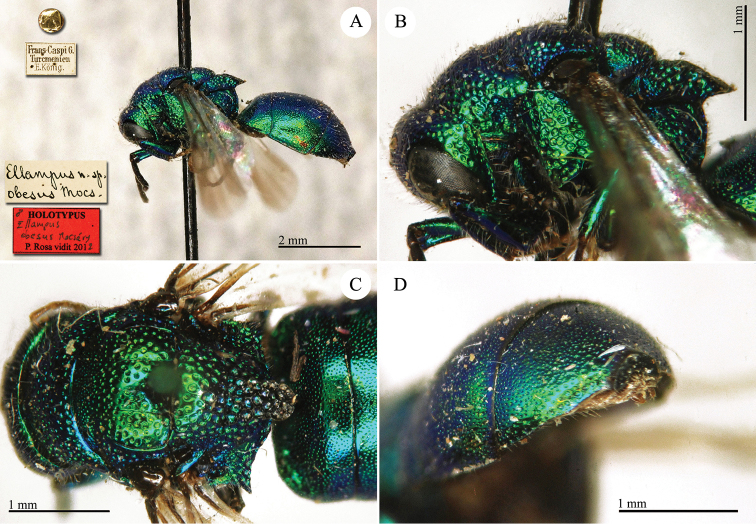
Ellampus (Notozus) obesus Mocsáry, 1890, holotype. **A** Habitus, lateral view **B** head and mesosoma, lateral view **C** mesosoma, dorsal view **D** third metasomal tergite, posterior view.

##### Current status.

*Elampus obesus* (Mocsáry, 1890).

#### Ellampus spinipes

Taxon classificationAnimaliaHymenopteraChrysididae

Mocsáry, 1890

Plate 60

Ellampus (Notozus) spinipes Mocsáry 1890: 49.

##### Type locality.

“Patria: Mongolia meridionalis (Ta-wan), a Cl. G. N. Potanin detectus (Coll. Radoszkovszkyi)”.

##### Holotype

♀ [box 59]: golden rounded label // Mongol. mer. Ta-wan 13/VII [handwritten] // Ellampus n.sp. spinipes Mocs [handwritten by Mocsáry].

##### Remarks.

The type lacks the left mid-leg.

**Plate 60.**
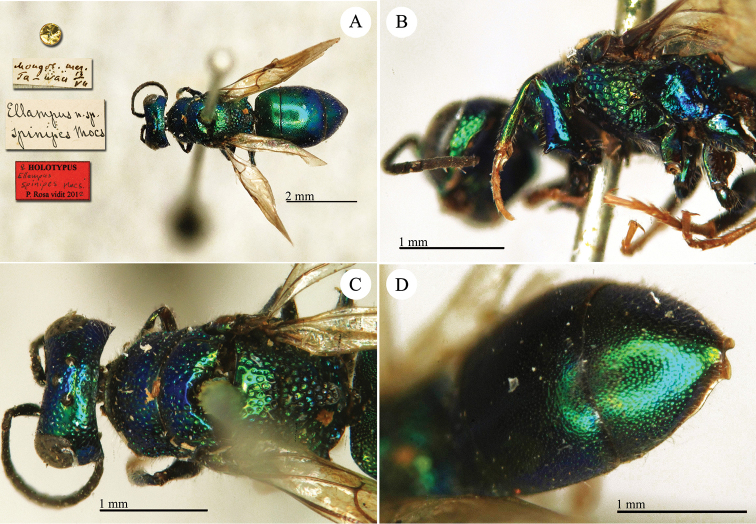
Ellampus (Notozus) spinipes Mocsáry, 1890, holotype. **A** Habitus, dorsal view **B** fore-leg, spine on femur **C** head and mesosoma, dorsal view **D** third metasomal tergite, dorso-lateral view.

##### Current status.

*Elampus spinipes* (Mocsáry, 1890) (transferred by Kimsey and Bohart 1991: 171).

#### Ellampus turkestanicus

Taxon classificationAnimaliaHymenopteraChrysididae

Mocsáry, 1889

Ellampus Turkestanicus Mocsáry 1889: 101.

##### Type locality.

“Turkestania, Taschkend (Coll. Rad.)”.

##### Holotype

[sex unknown] [box 59]: 30 [printed] [light blue label] // Пейшамбе [printed] // Tachkent [handwritten by Radoszkowski] // *Ellampus Turkestanicus* Mocs [handwritten by Radoszkowski] // 194 [printed] [yellow label].

##### Remarks.

The type is seriously damaged. It lacks both antennae after the scapus, all the legs, wings and the metasoma. Based on the mesosoma punctuation it belongs to the genus *Pseudomalus*. In the Mocsáry collection (HNHM) there are six specimens labelled as autotypes (from type n° 134857 to type n° 134862), which are not part of the type series, but they were collected in “Turkestan”, after the description.

##### Current status.

*Pseudomalus turkestanicus* (Mocsáry, 1889) (transferred by Kimsey and Bohart 1991: 270).

#### Ellampus violascens

Taxon classificationAnimaliaHymenopteraChrysididae

Mocsáry, 1889

Plate 61

Ellampus (Notozus) violascens Mocsáry 1889: 81.

##### Type locality.

“Patria: Turkestania (Taschkend, Coll. Rad.)”.

##### Holotype

♀ [box 59]: golden rounded label // Ϲъіръ-Дарья [printed] // 106// Ellampus n.sp. violascens Mocs [handwritten by Radoszkowski].

##### Remarks.

The type lacks the left antenna, the left mid-leg and the metasoma.

**Plate 61.**
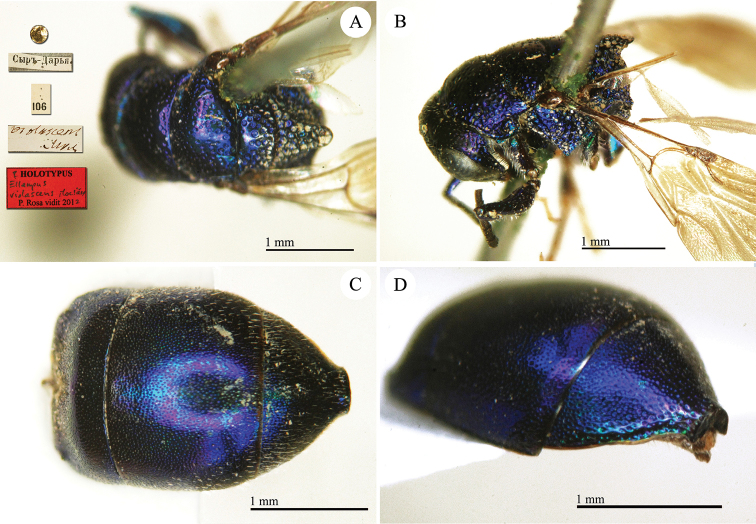
Ellampus (Notozus) violascens Mocsáry, 1889, holotype. **A** Mesosoma, dorsal view **B** mesosoma, lateral view **C** metasoma, dorsal view **D** third metasomal tergite, dorso-lateral view.

##### Current status.

*Elampus violascens* (Mocsáry, 1889) (transferred by Kimsey and Bohart 1991: 172).

#### Euchroeus amabilis

Taxon classificationAnimaliaHymenopteraChrysididae

Mocsáry, 1889

Euchraeus [sic!] amabilis Mocsáry (Inédite) (in Radoszkowski) 1889: 36.

##### Type locality.

“Senegal”.

##### Syntype

1♂ [box 62]: golden rounded label // Seneg. [printed] [green label] // *amabilis* Mocs [handwritten by Radoszkowski].

##### Syntype

1♀ [box 62]: Seneg. [printed] [green label] // label with genitalia.

##### Current status.

*Euchroeus candens* Dahlbom, 1854 (synonymised by Madl and Rosa 2012: 85).

#### Hedychrum callosum

Taxon classificationAnimaliaHymenopteraChrysididae

Radoszkovsky, 1877

Figure 8

Hedychrum callosum Radoszkovsky 1877 (1876): 108.

##### Type locality.

“Syra”.

##### Holotype

♂ [box 59]: golden rounded label // Syra [handwritten] // *Holopyga ahenea* Dhlb. (*Hedychrum callosum* Rad.) [handwritten by Mocsáry] // 84 [printed].

##### Remarks.

*Hedychrum callosum* Radoszkovsky, 1877 was considered a synonym of *Hedychridium aheneum* (Dahlbom, 1854) by Mocsáry (1887: 13; 1889: 146), Dalla Torre (1892: 20), and Bischoff (1913: 14). Trautmann (1927: 56) considered *Hedychridium aheneum* as a variety of *Hedychridium incrassatum* (Dahlbom, 1854), and therefore placed *Hedychrum callosum* in the synonymic list of *Hedychridium incrassatum*. The following authors (e.g. Linsenmaier 1951: 98; Kimsey and Bohart 1991: 196) re-evaluated *Hedychridium aheneum*, but *Hedychrum callosum* remained in synonym with *Hedychridium incrassatum*. *Hedychridium incrassatum* is found only in the western Mediterranean countries, while *Hedychridium aheneum* is spread in the eastern Mediterranean countries, Middle East and central Asia (Linsenmaier 1959, 1968). In southern Italy both *Hedychridium aheneum* and *Hedychridium incrassatum* (described from Sicily) are present (Strumia 1995).

**Figure 8.**
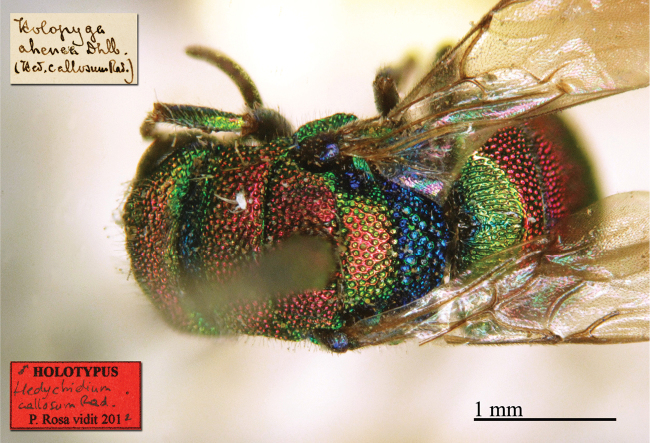
*Hedychrum callosum* Radoszkovsky, 1877, holotype, habitus, dorsal view.

##### Current status.

*Hedychridium aheneum* (Dahlbom, 1854) (synonymised by Mocsáry 1887).

#### Hedychrum cyaneum

Taxon classificationAnimaliaHymenopteraChrysididae

Mocsáry, 1889

Hedychrum cyaneum Mocsáry (in Radoszkowski) 1889: 10 *nec* Brullé, 1846.

##### Type locality.

“Sibérie orientale”.

##### Paralectotype

1♂ [box 59]: golden rounded label // Siberie Orient. [printed] // 109 // *cyaneum* Mocs. [handwritten by Mocsáry].

##### Remarks.

Mocsáry described *Hedychrum cyaneum* (later replaced by *Hedychrum simile*) based on a type series [♂♀]. In the original description (Mocsáry (in Radoszkowski) 1889), the only type locality is Siberia orientalis. But in his monograph (Mocsáry 1889: 158, under the replacement name *Hedychrum simile*), he specified: Siberia orientalis (Coll. Rad.! et Mus. Hung.); China borealis (Ta-tschian-sy, Mus. Hung.). The lectotype designated by French (in Bohart and French 1986), from China, is the female listed by Mocsáry.

##### Current status.

*Hedychrum simile* Mocsáry, 1889, replacement name for *Hedychrum cyaneum* Mocsáry 1889 (Mocsáry 1889: 158).

#### Hedychrum flavipes

Taxon classificationAnimaliaHymenopteraChrysididae

Eversmann, 1857

Figure 9

Hedychrum flavipes Eversmann 1857: 552.

##### Type locality.

“Hab. in campis orientalibus et in promontoriis Uralensibus”.

##### Syntype

1♀ [box 59]: golden rounded label // Hedychrum n. sp. flavipes Evm. [handwritten by Eversmann] // [handwriting not readable] // 79 [printed].

##### Remarks.

The type was originally pinned and later glued on a plastic label with extended ovipositor. The type is partially damaged: the left antenna is broken, glued on the label, and it lacks the tarsus of left mid-leg, as well as two terminal tarsal segments of the right mid-leg.

Semenov-Tian-Shanskij (1954) described the genus *Colpopyga* based on *Hedychrum flavipes* Eversmann. This species has the metasomal external segments morphologically modified. Noskiewicz and Lorencowa (1963) demonstrated that also the internal segments have a deep modified shape. Future molecular analysis may clarify the systematic position of this taxon.

**Figure 9.**
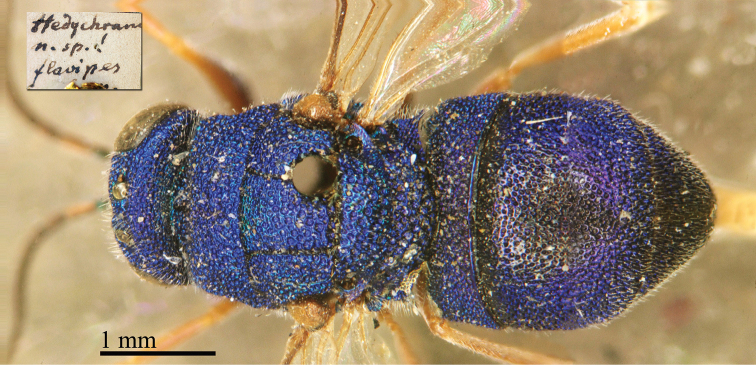
*Hedychrum flavipes* Eversmann, 1857, syntype, habitus, dorsal view.

##### Current status.

*Hedychridium flavipes* (Eversmann, 1857) (transferred by du Buysson (in André) 1891: 182).

#### Hedychrum mlokosiewitzi

Taxon classificationAnimaliaHymenopteraChrysididae

Radoszkovsky, 1877

Figure 10

Hedychrum Mlokosiewitzi Radoszkovsky 1877 (1876): 109.

##### Type locality.

“Envoyé du Caucase par Mr. Mlokosiewitz”.

##### Syntypes

6♂♂ and 2♀♀ [box 59]: Cauca Mlokos [some with label: Caucas Mloko] [printed].

##### Remarks.

One syntype was dissected and the genital capsule was glued on a label; this specimen bears a supplementary label: “21” [handwritten]; another male bears a square golden label. One additional syntype is housed in MNHN (general collection, box 11), in MNHU (box 143.8) and in MSNG (Rosa 2009). In Radoszkowski (1889) the name is cited as *Hedychrum Mlokosewitzi* Rad., which is an incorrect subsequent spelling.

**Figure 10.**
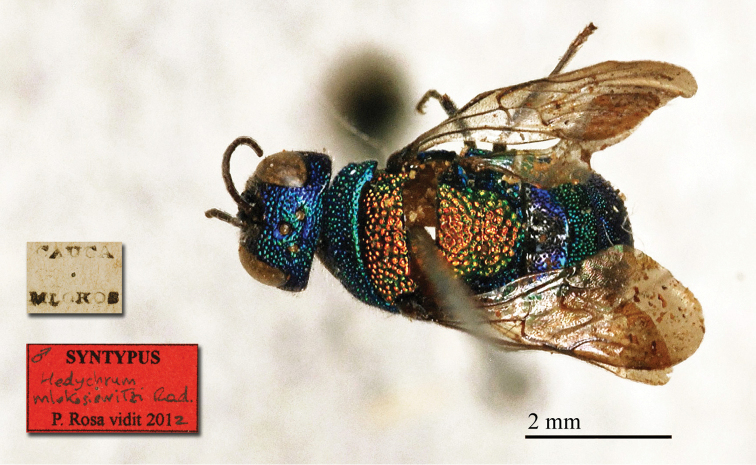
*Hedychrum mlokosiewitzi* Radoszkovsky, 1877, syntype, habitus, dorsal view.

##### Current status.

*Holopyga mlokosiewitzi* (Radoszkovsky, 1877) (transferred by Radoszkovsky 1889: 9).

#### Hedychrum radoszkowskyi

Taxon classificationAnimaliaHymenopteraChrysididae

du Buysson, 1893

Figure 11

Hedychrum radoszkowskyi du Buysson (in André) 1893: 213.

##### Type locality.

“Algérie (Radoszkowsky)”.

##### Holotype

♂ [box 59]: Africa [printed] [light blue label] // plastic label with left fore wing glued on it // *Hedychrum Radoszkowskyi* Buyss. n. sp! [handwritten by Buysson].

##### Remarks.

The type is partially damaged: it lacks the right antenna and left flagellum. The type locality is different from the one given by du Buysson: Africa instead of Algeria. This information is particularly important, because the species has a typical sub-saharan aspect, and it does not belong to the Palaearctic fauna, as supposed by all the authors. Linsenmaier (1999: 45) in his last revision on the northern African species considered *Hedychrum radoszkowskyi* as belonging to the Egyptian fauna (sic!), known only from the type “*Mir nicht in Natura bekannt*”.

**Figure 11.**
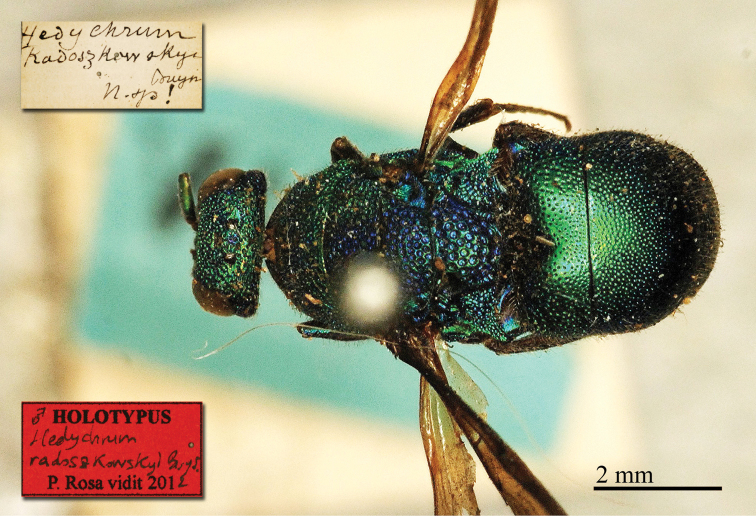
*Hedychrum radoszkowskyi* du Buysson, 1893, holotype, habitus, dorsal view.

##### Current status.

*Hedychrum radoszkowskyi* du Buysson, 1893.

#### Hedychrum solsky

Taxon classificationAnimaliaHymenopteraChrysididae

Radoszkowski, 1877

Plate 62

Hedychrum Solsky Radoszkowski 1877: 7.

##### Type locality.

“Habitat in desertis Kisil-kum, in Bairacum et in Ferghana”, “Видъ этотъ пойман въ 1871 г. 1 мая въ Кизилъ-кумахъ, 3, 17 и 19 мая въ Байракумѣ, 29 и 30 іюня въ Сохѣ.” [This species was collected on the 1^st^ of May 1871 at Kisilkumah, on the 3^rd^, 17^th^, and 19^th^ of May in Bairacum, on the 29^th^ and 30^th^ of June at Sokha].

##### Syntype

1♂ [box 59]: 30 [printed] [blue-green label with red line] // Кизилъкумъ // *solsky* [handwritten by Radoszkowski] // label with genital capsula.

##### Syntype

1♂ [box 59]: 30 [printed] [blue-green label with red line] // Кизилъкумъ.

##### Syntype

1♀ [box 59]: golden rounded label // Кизилъкумъ // 14 [printed] [pink label with red line] // 78 [printed].

##### Remarks.

The collecting dates do not match the localities given in the original description. However, the same situation was found in the other four specimens housed in the Fedtschenko collection in MMU. Therefore a case of *lapsus calami* must be happened and we consider these specimens and those in MMU as syntypes. Another possible male syntype collected at Kizil-kum by Fedtschenko was also found in Gribodo collection (MSNG, not listed in Rosa 2009).

Mocsáry (1889: 116) introduced the correct emendation *Hedychrum solskyi*. In Radoszkowski (1889) and Kimsey and Bohart (1991: 235) it was listed as *Hedychrum solskii*.

**Plate 62.**
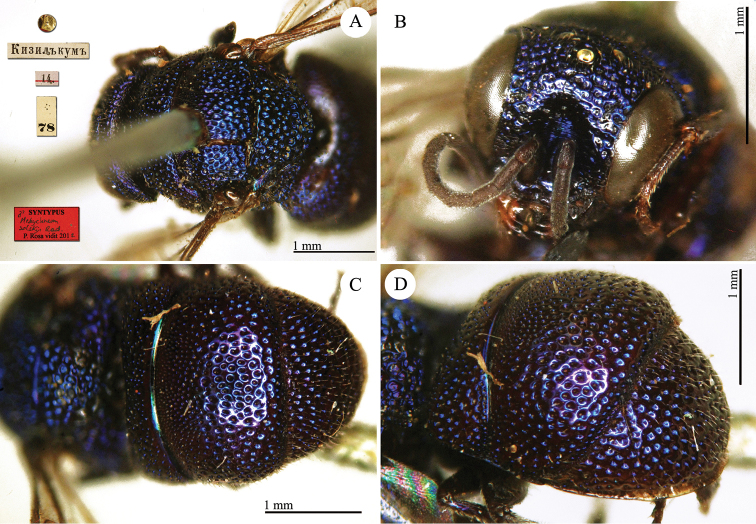
*Hedychrum solsky* Radoszkowski, 1877, syntype. **A** Mesosoma, dorsal view **B** head, frontal view **C** metasoma, dorsal view **D** metasoma, dorso-lateral view.

##### Current status.

*Holopyga solskyi* (Radoszkowski, 1877) (transferred by Radoszkowski 1889: 7).

#### Holopyga caspica

Taxon classificationAnimaliaHymenopteraChrysididae

Mocsáry, 1890

Plate 63

Holopyga (Hedychridium) caspica Mocsáry 1890: 53.

##### Type locality.

“Patria: Territorium Maris Caspii (Coll. Radoszkovszkyi)”.

##### Holotype

♀ [box 59]: golden rounded label // M. Casp. occ. [printed] [light red label] // Holopyga n.sp. caspica Mocs. [handwritten by Mocsáry].

##### Remarks.

The type lacks the last two flagellomeres of the left antenna, the left fore wing, and two terminal tarsal segments of the left hind-leg.

**Plate 63.**
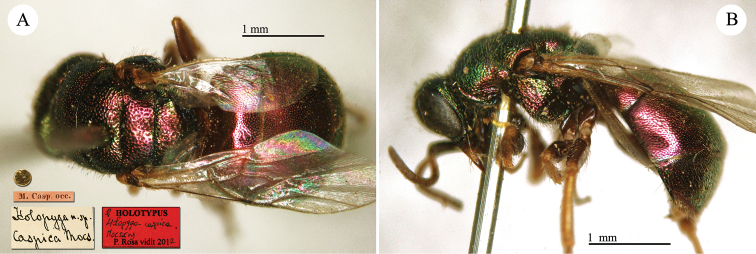
Holopyga (Hedychridium) caspica Mocsáry, 1890, holotype. **A** Habitus, dorsal view **B** habitus, lateral view.

##### Current status.

*Hedychridium caspicum* (Mocsáry, 1890) (transferred by du Buysson (in André) 1891: 189).

#### Notozus komarowi

Taxon classificationAnimaliaHymenopteraChrysididae

Radoszkowski, 1893

Plate 64

Notozus Komarowi Radoszkowski 1893b (1892): 79.

##### Type locality.

“Merw”.

##### Holotype

♀ [box 59]: golden rounded label // Semsau Merw [printed] [yellow label] // *Komarowi* Rd [handwritten by Radoszkowski].

##### Remarks.

The type is damaged, missing antennae and femora of the fore- and mid-legs. The metasoma is glued on a label. It corresponds to the female of *Elampus obesus* (Mocsáry, 1890). Here it is proposed as new synonym of *Elampus komarowi* (Radoszkowski, 1893) = *Elampus obesus* (Mocsáry, 1890).

**Plate 64.**
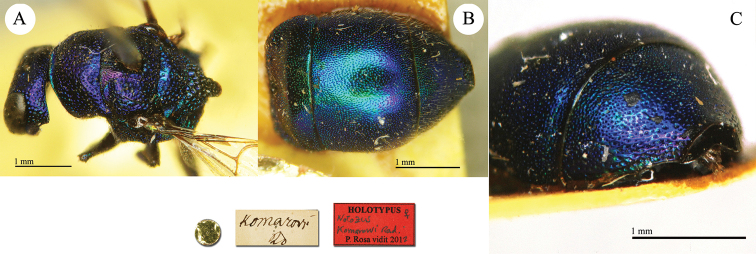
*Notozus komarowi* Radoszkowski, 1893, holotype. **A** Mesosoma, dorso-lateral view **B** metasoma, dorsal view **C** third metasomal tergite, posterior view.

##### Current status.

*Elampus obesus* (Mocsáry, 1890).

#### Notozus productus var. vulgatus

Taxon classificationAnimaliaHymenopteraChrysididae

du Buysson, 1892

Notozus productus var. vulgatus du Buysson (in André) 1892: 100.

##### Type locality.

“France, Belgique, Allemagne, Russie, Suisse, Grèce, Turkestan.”.

##### Syntype

1♀ [box 59]: Atrek [handwritten by Radoszkowski] [yellow label] // [undreadble] [handwritten] // Notozus productus Dahlb. var. vulgatus Buyss. [handwritten by du Buysson].

##### Remarks.

This syntype lacks the head. This syntype belongs to or is closely related to *Elampus constrictus* (Förster) *sensu* Móczár (1964a).

##### Current status.

*Elampus spina* (Lepeletier, 1806) (synonymised and transferred by Kimsey and Bohart 1991: 171).

#### Olochrysis eldari

Taxon classificationAnimaliaHymenopteraChrysididae

Radoszkowski, 1893

Plate 65

Olochrysis Eldari Radoszkowski 1893a: 242.

##### Type locality.

“Caucase, Eldar”.

##### Holotype

♀ [box 60]: Eldar Caucas [printed] // *Eldari* [handwritten by Radoszkowski?].

##### Remarks.

Kimsey and Bohart (1991: 489) placed *Olochrysis eldari* in the genus *Chrysura* Dahlbom, *Chrysis radians* group, without type examination. It belongs to the genus *Chrysis*, *elegans* group. It is the first available name for Chrysis angustifrons var. ignicollis Trautmann, 1926. Chrysis angustifrons var. ignicollis was described as a variety of *Chrysis angustifrons* and it was elevated to species rank by Linsenmaier (1959). Linsenmaier (1959, 1968, 1987) did not list *Chrysis eldari* in his revisions. We propose the new combination: *Chrysis eldari* (Radoszkowski 1893), and the new synonym: Chrysis angustifrons var. ignicollis Trautmann, 1926 = *Chrysis eldari* (Radoszkowski, 1893). In HNHM, we examined male and female specimens of *Chrysis rubricollis* du Buysson, 1900 collected at Burnabat (Turkey) and identified by du Buysson and Mocsáry. They all match the original description and belong to *Chrysis eldari*. Therefore we also propose the new synonym: *Chrysis rubricollis* du Buysson, 1900 = *Chrysis eldari* (Radoszkowski, 1893).

**Plate 65.**
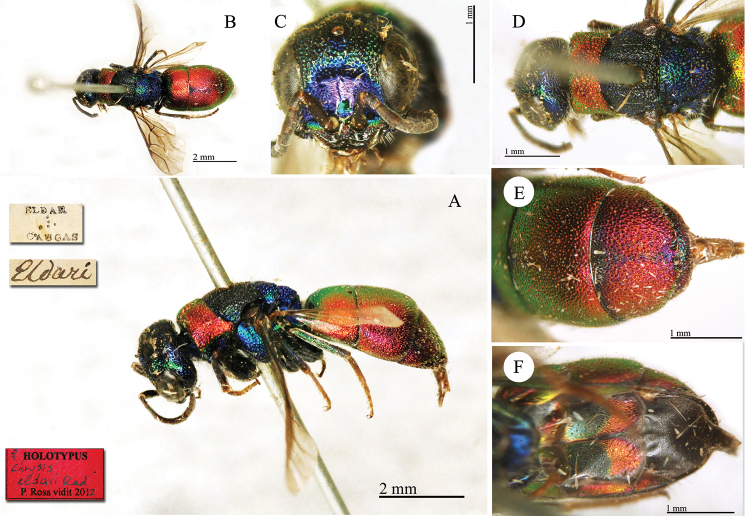
*Olochrysis eldari* Radoszkowski, 1893, holotype. **A** Habitus, lateral view **B** habitus, dorsal view **C** head, frontal view **D** mesosoma, dorsal view **E** third metasomal tergite in dorsal view **F** metasoma, ventral view.

##### Current status.

*Chrysis eldari* (Radoszkowski, 1893), **comb. n.**.

#### Parnopes popovii

Taxon classificationAnimaliaHymenopteraChrysididae

Eversmann, 1857

Figure 12

Panorpes [sic!] popovii Eversmann 1857: 615.

##### Type locality.

“Hab. in campis orientalibus”.

##### Holotype

♀ [box 62]: golden rounded label // red rounded label // *Panorpes popovii* Evm. [handwritten by Eversmann] // [unreadable].

**Figure 12.**
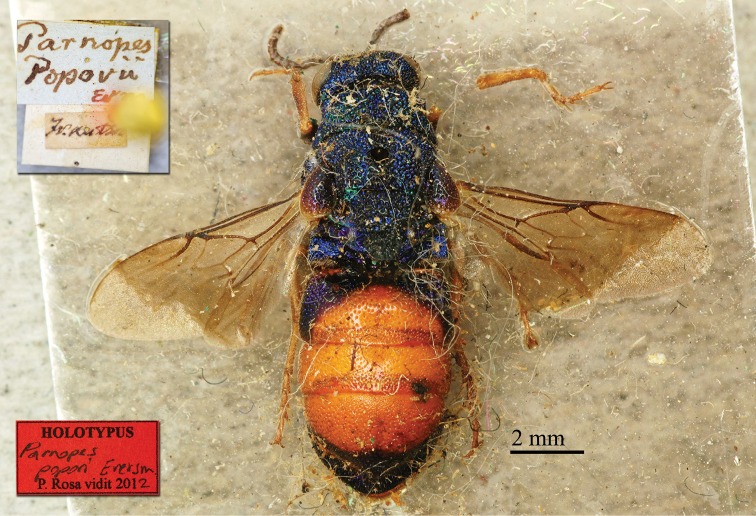
*Parnopes popovii* Eversmann, 1857, holotype, habitus, dorsal view.

##### Current status.

*Parnopes popovii* Eversmann, 1857.

#### Spintharis mocsaryi

Taxon classificationAnimaliaHymenopteraChrysididae

Radoszkowski, 1890

Spintharis Mocsaryi Radoszkowski 1890: 508.

##### Type locality.

unknown. “Ararat, entre Sardar-Abadu et Sarabandy (13,000’)” [given in the introduction].

##### Holotype

♂ [box 60]: golden rounded label // Caucas Mlok [printed] // *Mocsary* [handwritten by Radoszkowski].

##### Remarks.

The specimen found in the Radoszkowski collection under the name *Spintharis mocsaryi* was collected by Mlokosewicz in “Caucasus”. In the original description, the locality is not given. The specimen matches the original description. The most important authors (Mocsáry 1889, Dalla Torre 1892, Bischoff 1913, du Buysson (in André) 1896, Zimmermann 1927, Balthasar 1953, and lastly Linsenmaier 1968) considered *Spintharis mocsaryi* as a valid species. It is a synonym of *Spintharina vagans* (Radoszkowski). Bohart (1987) did not list *Spintharis mocsaryi* in the key to the genus *Spintharis*, probably he already considered *Spintharis mocsaryi* as a synonym of *Spintharina vagans*.

##### Current status.

*Spintharina vagans* (Radoszkowski, 1877) (synonymised by Kimsey and Bohart 1991: 558).

#### Stilbum splendidum var. caspicum

Taxon classificationAnimaliaHymenopteraChrysididae

du Buysson, 1896

Stilbum splendidum var. caspicum du Buysson (in André) 1896: 680.

##### Type locality.

“Patrie: Province Transcaspienne: Otrek (Radoszkowsky); Abyssinie (J. de Gaulle)”.

##### Syntype

1♂ [box 60]: Atrek [handwritten] [yellow label] // Stilbum spledidum var. caspicum Buyss. [handwritten by du Buysson].

##### Remarks.

*Stilbum splendidum caspicum* is one of the colour variations of *Stilbum cyanurum* (Forster, 1771).

##### Current status.

*Stilbum cyanurum* (Forster, 1771).

### Notes on other specimens in the Radoszkowski collection

#### Chrysis imperatrix

Taxon classificationAnimaliaHymenopteraChrysididae

du Buysson, 1887

Chrysis imperatrix du Buysson 1887: 190.

##### Remarks.

The holotype is housed in MNHN (general collection, box 47). The rest of the original series is in Kraków: one male (Ctenb m. d u t [printed], 19 [printed, pink label], label with genitalia; box 61) and one female (TR-Cap Saraks [printed]). However the two specimens must be excluded from the type series because the author based his description on a single specimen, which must be considered as holotype by monotypy: “*Je possède un spécimen qui m’a été envoyé de Russie par M. le général O. Radoszkowsky, bien connu par ses écrits hyménoptérogiques*.” It belongs to the *comparata* group.

#### Chrysis radoszkowskyi

Taxon classificationAnimaliaHymenopteraChrysididae

Gribodo, 1879

Chrysis Radoszkowskyi Gribodo 1879: 358.

##### Remarks.

In the original description Gribodo listed only two specimens: one in his collection (Rosa 2009) and one in the Drewsen collection. In Radoszkowski’s collection, there are other specimens from the original series: four specimens collected in Australia, two females and two males [box 60]. The males are marked with golden labels - one rounded and one square and they belong to two different species; the specimen with the square one bears a label with the name *Radoszkowsky* [handwritten by Gribodo?]; these specimens cannot be considered as syntypes, since they were not included in the original series and there is any evidence to state that Gribodo examined them.

##### Current status.

*Primeuchroeus radoszkowskyi* (Gribodo, 1879) (transferred by Kimsey and Bohart 1991: 542).

#### Chrysis speciosa

Taxon classificationAnimaliaHymenopteraChrysididae

Radoszkowski, 1877

Chrysis speciosa Radoszkowski 1877: 17.

##### Type locality.

“Habitat in deserto prope Taschkent”, “Пойманы 19 мая 1871 г. въ степи между Сыръ-дарьей и Ташкентомъ” [collected on the 19^th^ of May 1871 in steppe between Syr-Darya and Tashkent].

##### Paralectotype

[?] 1♂ [box 61]: golden rounded label // Ворухъ [printed] // 19. [printed] [blue-green label with red line] // *speciosa* [hadwritten by Radoszkowski].

##### Remarks.

Bohart (in Kimsey and Bohart 1991: 464) designated the lectotype in MMU and placed *Chrysis speciosa* in the *maculicornis* group. Radoszkowski indicated only the female in the description of *Chrysis speciosa* on pag. 17, but he gave no further informations or type locality for the var. β described at pag. 18. The specimen was collected at Ворухъ [= Vorukh], locality not mentioned in the description. The specimen housed in Kraków could be referred to the β variety but belongs to the *comparata* group.

##### Current status.

*Chrysis speciosa* Radoszkowski, 1877.

#### Chrysis varicornis

Taxon classificationAnimaliaHymenopteraChrysididae

Radoszkowski, 1877 nec Spinola, 1838

Chrysis varicornis Radoszkowski 1877: 11.

##### Remarks.

Five specimens were placed under the name *Chrysis picticornis* Mocs. [box 60]. Three of them were collected in Caucasus and cannot be considered as syntypes, because the original type locality is Zaravshan Valley. Two females were collected at Taschkent. Usually specimens collected in Turkestan (Radoszkowski 1877) bear labels in Cyrillic. In this case, localities are all written in Latin and for this reason they could not be considered as types. The first specimen bears the handwritten label by Radoszkowski: *picticornis* Mocs. It belongs to the *radians* group.

##### Current status.

*Chrysura sulcata* Dahlbom, 1845 (synonymised by Linsenmaier 1951: 106; transferred by Kimsey and Bohart 1991: 496).

#### Hedychrum lama

Taxon classificationAnimaliaHymenopteraChrysididae

du Buysson, 1891

Hedychrum lama du Buysson 1891a: 31.

##### Remarks.

The type locality is “Mongolie: Kansu-Kobden-Owatu”. Kimsey (in Kimsey and Bohart 1991: 215) designated the lectotype at MNHN. In MNHN (box 17 in the general collection) under the name *Hedychrum lama* there are two specimens: one from Mongolia, labelled by du Buysson as “type”, and bearing a red label “Type” pinned by someone else; the other specimen from Quetta, Pakistan (Baluchistan, leg. Nurse, 1904), was labelled by du Buysson as “type”. None bears Kimsey’s lectotype label. The specimen from Pakistan is not a syntype, since it is not mentioned in the type series, and it was collected or received after the date of description. The female housed in Kraków (label: *Hedychrum lama* Buyss. ♀ [handwritten by du Buysson] // Kansu Taitong-Che 1/V 1886 [handwritten]) was later cited by du Buysson (1893: 247) and it is not a type. The specimen from Mongolia in MNHN must be considered as a holotype by monotypy.

### Types not found in the Radoszkowski collection

Kimsey and Bohart (1991) listed nine other taxa described by different authors whose types should be housed in Kraków, but there is no other published evidence that they were placed in Kraków. They could be housed in other collections (e.g. MNHN, HNHM, MNHU and so on); they could also be hidden in the Radoszkowski collection and we were not able to recognize them.

The nine types not found in the collection are: *Chrysis alexandri* du Buysson (currently *Spintharina*), *Chrysis angolensis* Radoszkovsky, *Chrysis baeri* Radoszkovsky (currently *Chrysura*), *Chrysis diacantha* Mocsáry, Chrysis humboldti var. minor Mocsáry (currently *Pseudospinolia*), *Chrysis olivierii* Radoszkowski, *Chrysis pyrocoelia* Mocsáry (currently *Chrysura*), *Chrysis undulata* Radoszkovsky, and Parnopes grandior var. caspicus Radoszkowski.

## Conclusions

Radoszkowski is considered as a pioneer in the study of Chrysididae as he described a large number of species, he was the first author to study the Central Asiatic chrysidis, and because he was the first who recognised the taxonomic importance of male genitalia in this family. However, the types and other specimens included in his collection were not available during the major revisional works (Trautmann 1927; Balthasar 1953; Linsenmaier 1959, 1968; Kimsey and Bohart 1991). Also Semenov-Tian-Shanskij and Nikol’skaja could not study Radoszkowski specimens housed in Kraków and examined only the types preserved in the Fedtschenko collection in MMU (Semenov-Tian-Shanskij 1932). Since there are no published images of the types housed in Radoszkowski collection and because some type specimens had been misinterpreted in the past, the catalogue is illustrated with images to facilitate future identifications.

In the present paper, we arrange for the first time eleven species in species-groups and we change species group for seven species; we confirm that ninety-three primary types by du Buysson, Eversmann, Mocsáry and Radoszkowski are preserved in the Radoszkowski collection. In Kimsey and Bohart (1991) they were placed in Kraków doubtfully. Moreover we found that the types of other three species (*Chrysis asiatica* Radoszkowski, 1889, Chrysis indigotea var. daghestanica Mocsáry, 1889, and *Chrysis octavii* du Buysson, 1895) are here deposited; lastly we found that types of seven taxa (*Brugmoia pellucida* Radoszkowski, 1877, *Chrysis barrei* Radoszkowski, 1891, *Chrysis sabulosa* Radoszkowski, 1877, *Ellampus hypocrita* du Buysson, 1893, *Hedychrum solskyi* Radoszkowski, 1877, Notozus productus var. vulgatus du Buysson, 1892, Stilbum splendidum var. caspicum du Buysson, 1896) are housed in the Radoszkowski collection and not only in MNHU, MNHN or MMU.
